# NMJ-related diseases beyond the congenital myasthenic syndromes

**DOI:** 10.3389/fcell.2023.1216726

**Published:** 2023-08-04

**Authors:** Alejandra Navarro-Martínez, Cristina Vicente-García, Jaime J. Carvajal

**Affiliations:** Centro Andaluz de Biología del Desarrollo, CSIC-UPO-JA, Universidad Pablo de Olavide, Sevilla, Spain

**Keywords:** neuromuscular junction, synaptic transmission, human disease, mouse models, comparative analysis

## Abstract

Neuromuscular junctions (NMJs) are a special type of chemical synapse that transmits electrical stimuli from motor neurons (MNs) to their innervating skeletal muscle to induce a motor response. They are an ideal model for the study of synapses, given their manageable size and easy accessibility. Alterations in their morphology or function lead to neuromuscular disorders, such as the congenital myasthenic syndromes, which are caused by mutations in proteins located in the NMJ. In this review, we highlight novel potential candidate genes that may cause or modify NMJs-related pathologies in humans by exploring the phenotypes of hundreds of mouse models available in the literature. We also underscore the fact that NMJs may differ between species, muscles or even sexes. Hence the importance of choosing a good model organism for the study of NMJ-related diseases: only taking into account the specific features of the mammalian NMJ, experimental results would be efficiently translated to the clinic.

## 1 Introduction

Neuromuscular junctions (NMJs) have been the subject of study since the early 19th century and are the ideal model for understanding synapses. Their main function is the transmission of signals between the motor neuron (MN) and the skeletal muscle fiber to induce muscle contraction and movement (Kuhne, 1863; Dale, 1914; Couteaux, 1947; Fatt and Katz, 1951; Fatt and Katz, 1952; Birks et al., 1960).

The position of the NMJ in the muscle fiber, its morphology, or degree of development of the postsynaptic region may vary between muscles or species; but despite these particularities, all NMJs contain these main components: 1) a non-myelinating terminal Schwann cell (tSC) that forms a “cap” over the part of the nerve terminal that does not face the postsynaptic region and that is important for synaptogenesis, synaptic transmission, and NMJ maintenance and repair (Santosa et al., 2018); 2) a nerve terminal filled with neurotransmitter vesicles; 3) the synaptic cleft in between the MN and its innervating muscle; 4) the postsynaptic membrane containing neurotransmitter receptors; and 5) the muscle sarcoplasm that provides structural and metabolic support (Engel, 2008). Acetylcholine (ACh) is the neurotransmitter involved in synaptic transmission in vertebrate NMJs. After ACh is released from the presynaptic nerve terminal into the synaptic cleft, it binds to nicotinic ACh receptors (AChR) in the postsynaptic muscle region triggering a response in the muscle (Engel, 2008).

Mutations in proteins located at the NMJ cause the so-called congenital myasthenic syndromes, inherited neuromuscular disorders characterized by abnormal NMJ morphology and/or signal transmission at the motor endplate (Engel, 2020). In this review we aim at highlighting potential novel candidate genes causative or modifiers of NMJ-related disorders based on data from already-published mouse models. Further, we seek to draw attention on very important factors that need to be taken into account in the diagnosis and treatment of NMJ-related disorders. Indeed, while the mouse is the model organism of choice in the study of human diseases, it may not be the most informative in the case of NMJ-related diseases. Further, only a few muscle types, and normally only in males, are usually analyzed. Thus, caution should be taken when translating to the clinic findings derived from studies that disregard these factors.

## 2 Novel candidate genes potentially playing a role in human NMJ disorders

In 1991, the first printed version of the Gene Table of Neuromuscular Disorders was published (Dubowitz, 1991). It contained a total of 7 nuclear genes and 16 mapped loci, together with their associated neuromuscular disease. This table has been annually updated ever since, and besides de printed version, a computerized online version that allows interactivity is also available at www.musclegenetable.fr. This database currently hosts 641 unique genes and 1,129 diseases grouped into 16 distinctive categories (Cohen et al., 2021). Group 11 contains the congenital myasthenic syndromes, with a total of 42 such syndromes caused by mutations in 34 unique genes; most of them being AChR subunits or genes involved in AChR clustering or ACh metabolism (Engel, 2020). Remarkably, genes associated with other diseases also affect the NMJ, especially genes related with MN diseases, but also with “pure” muscular dystrophies or myopathies. That is the case of STAC3, a component of the excitation-contraction coupling machinery (Polster et al., 2016) whose mutations can be found in patients with Bailey-Block congenital myopathy (Horstick et al., 2013; Online Mendelian Inheritance in Man, OMIM^®^. Johns Hopkins University, Baltimore, MD. MIM Number: 255995: 10/03/2023. World Wide Web URL: https://omim.org/). Also known as Native American myopathy because it was first described in the Lumbee Indian tribe in North Carolina (Bailey and Bloch, 1987). This autosomal recessive disorder is characterized by congenital weakness, arthrogryposis and myopathic facies, among multiple other symptoms. KO mice for the gene show perinatal lethality due to their inability to breathe, as well as a wide array of muscular defects (Nelson et al., 2013; Reinholt et al., 2013). While the NMJs of these mutants are correctly formed, they occupy a broader space, presenting an increase in the frequency of miniature endplate potentials, likely as a secondary consequence of the muscle defects (Nelson et al., 2013), but underscoring the interdependence of MN and muscle development for correct morphology and synaptic transmission at the NMJ. However, only in a few neuromuscular disorders, potential phenotypes of the NMJ have been directly addressed in humans. This can be probably explained by the limited availability of samples, the difficulty in accurately assessing NMJ function in humans or simply due to the fact that the multitude of other neural or muscle-specific phenotypes are of higher priority because of prevalence or clinical significance. In any case, there exists the possibility that genes involved in already-described diseases, also alter the NMJ, potentially impacting on the pathophysiological mechanisms or disease treatments.

This observation prompted us to review the literature in search of mouse gene models that have already shown any abnormality related with NMJ morphology or function. All these genes could be potential modifiers of some diseases or even the cause of the few for which the gene has not been mapped yet, as is the case of autosomal recessive distal spinal muscular atrophy motor neuron disease (Online Mendelian Inheritance in Man, OMIM^®^. Johns Hopkins University, Baltimore, MD. MIM Number: 607088: 16/08/2019. World Wide Web URL: https://omim.org/).

The Mouse Genome Informatics MGI database (http://www.informatics.jax.org) currently harbors 258 single or multigenic mouse models in different genetic backgrounds, with annotations associated with abnormal NMJ structure or function. Specifically, the terms used to retrieve these results were: “abnormal neuromuscular synapse morphology”, “failure of neuromuscular synapse presynaptic differentiation”, “failure of neuromuscular synapse postsynaptic differentiation”, “abnormal endplate potential” and “abnormal miniature endplate potential”. These 258 models (Supplementary Table S1) are composed of 132 unique loci, 64 of which have not been associated with any human neuromuscular disease registered at the full gene table database (Supplementary Table S2), even if some of them are well-known players in NMJ formation, maintenance and function (Tables 1–9 and Supplementary Table S3). Possible explanations include the fact that most of these models are constitutive knock-outs (cKOs) that lead to embryonic or early postnatal lethality, usually related with the impossibility of inflating the lungs due to a lack of neural input to the diaphragm muscle (Tables 1–9 and Supplementary Table S3). Deficiencies in these genes would probably cause abortions or still-births in humans as well. However, they can still be candidates to take into account for potential single nucleotide polymorphisms (SNPs) as causative or modifiers of specific diseases.

**TABLE 1:** Novel candidate genes for NMJ-related diseases in humans associated with motor neuron identity, function and survival. All models are constitutive unless otherwise stated. Lethal phenotypes can have complete or incomplete penetrance. cKO, constitutive KO; MN, motor neuron; NMJ, neuromuscular junction; PM, point-mutation.

Gene	Mouse model	Lethality^a^	References
Pre-synaptic terminal			
*Adarb1/Adar2*	MN-specific KO (*VAChT-Cre*)	PD	Hideyama et al. (2010)
*Clp1*	Kinase-dead isoform	SD: V, NL or PD	Hanada et al. (2013)
*Gphn/Geph*	cKO	SD: V or NL	Feng et al. (1998), Banks et al. (2005)
*Mnx1/Hb9*	cKO	PNL	Arber et al. (1999), Thaler et al. (1999)
*Pex10*	Chemically induced PM affecting protein function	PNL	Hanson et al. (2014)
*Ret*	cKO and cranial MN-specific KO (*Nkx6.2-Cre*)	PNL (cKO)	Baudet et al. (2008), Zahavi et al. (2015)
*Slc6a5/Glyt2*	cKO by spontaneous retrotransposon insertion	PL	Gomeza et al. (2003), Bogdanik et al. (2012)

^a^ Key: AD, allele or model-dependent; EL, embryonic lethality; NL, neonatal lethality; PD, premature death; PNL, perinatal lethality; PL, postnatal lethality; SD, strain-dependent; V, viability.

**TABLE 2:** Novel candidate genes for NMJ-related diseases in humans associated with channels. All models are constitutive unless otherwise stated. Lethal phenotypes can have complete or incomplete penetrance. cKO, constitutive KO.

Gene	Mouse model	Lethality^a^	References
Pre-synaptic terminal			
*Cacna2d2*	Spontaneous mutation leading to a shorter transcript (*Ducky* mice)	SD: V or PD	Snell (1955), Barclay et al. (2001), Kaja et al. (2007)
*Cacng2*	Hypomorph by spontaneous transposon insertion (*Stargazer* mice)	—	Noebels et al. (1990), Letts et al. (1998), Kaja et al. (2007)
Synaptic cleft			
*Mmp3*	cKO	—	VanSaun et al. (2003)
Post-synaptic terminal			
*Csnk2b*	Skeletal muscle-specific KO (*ACTA1-Cre*)	—	Cheusova et al. (2006)
*Nes*	cKO	—	Mohseni et al. (2011), Yang et al. (2011), Lindqvist et al. (2017)
*Prkcq/PKCθ*	cKO	—	Li et al. (2004), Lanuza et al. (2010)
*Rer1*	Heterozygous cKO	- (EL cKO)	Valkova et al. (2011)

^a^ Key: AD, allele or model-dependent; EL, embryonic lethality; NL, neonatal lethality; PD, premature death; PNL, perinatal lethality; PL, postnatal lethality; SD, strain-dependent; V, viability.

**TABLE 3:** Novel candidate genes for NMJ-related diseases in humans associated with the regulation of gene expression, as well as with the processes of proteostasis and autophagy. All models are constitutive unless otherwise stated. Lethal phenotypes can have complete or incomplete penetrance. cKO, constitutive KO; MN, motor neuron; PM, point-mutation.

Gene	Mouse model	Lethality^a^	References
Pre-synaptic terminal			
*Borcs5*	cKO	PNL	de Pace et al. (2020)
*Dnmt3a*	Nervous system-specific KO (*Nes-Cre*)	PD	Nguyen et al. (2007)
*Eef1a2*	cKO by spontaneous PM (*Wasted* mice)	PD	Shultz et al. (1982), Chambers et al. (1998), Khalyfa et al. (2001), Newbery et al. (2005), Doig et al. (2013)
*Nemf*	cKO and chemically induced PM affecting protein function	AD: V or PD	Martin et al. (2020)
*miR-218–1/2*	cKO	NL	Amin et al. (2015)
*Plaa*	cKO and G23V PM recapitulating PLAA-associated neurodevelopmental disorder	AD: PD or PNL	Hall et al. (2017)
*Psmf1/PI31*	MN-specific KO (*Mnx1-Cre*)^b^	- (EL cKO)	Liu K et al. (2019), Minis et al. (2019)
*Rab18*	cKO	PNL	Carpanini et al. (2014)
*Tmem106b*	cKO	—	Lüningschrör et al. (2020)
*Tmem184b*	cKO	—	Bhattacharya et al. (2016)
*Usp14*	Hypomorphs by chemically induced PM (*nmf375* mice) and by spontaneous PM (*Ataxia* mice)	SD: V, PNL or PD	D’Amato and Hicks (1965), Wilson et al. (2002), Chen et al. (2009), Chen et al. (2011), Bhattacharyya et al. (2012), Marshall et al. (2013), Vaden et al. (2015)
Post-synaptic terminal			
*Mbnl1* and *Mbnl2*	*Mbnl1* ^*−/−*^ */Mbnl2* ^*+/−*^	PD (EL double KO)	Lee et al. (2013)
*miR-1/206/133*	cKO	PD	Williams et al. (2009), Liu et al. (2012), Klockner et al. (2022)
*Spin1*	Skeletal muscle-specific KO (*Myf5-Cre*)	NL	Greschik et al. (2017)

^a^ Key: AD, allele or model-dependent; EL, embryonic lethality; NL, neonatal lethality; PD, premature death; PNL, perinatal lethality; PL, postnatal lethality; SD, strain-dependent; V, viability.

^b^ See section *2.10. Other considerations* for further information regarding this line.

**TABLE 4:** Novel candidate genes for NMJ-related diseases in humans associated with cytoskeleton dynamics. All models are constitutive unless otherwise stated. Lethal phenotypes can have complete or incomplete penetrance. cKO, constitutive KO; MN, motor neuron; PM, point-mutation.

Gene	Mouse model	Lethality^a^	References
Pre-synaptic terminal			
*Basp1/Cap23*	cKO	PL	Frey et al. (2000)
*Clip3/CLIPR59*	cKO	PNL	Couesnon et al. (2013)
*Cpeb4*	Dominant negative truncated protein	NL	Shin et al. (2016)
*Fbxo45*	cKO	NL	Saiga et al. (2009)
*Phr1/Mycbp2*	cKO, MN-specific deletion (*Mnx1-Cre*)^2^, C-terminal truncated protein (*Magellan* mice), mutant lacking exons 8–9, and PM affecting protein function	AD: V, PNL or NL	Burgess et al. (2004), Bloom et al. (2007), Lewcock et al. (2007)
*Rnd3/RhoE*	cKO	PD	Mocholí et al. (2011)

^a^ Key: AD, allele or model-dependent; EL, embryonic lethality; NL, neonatal lethality; PD, premature death; PNL, perinatal lethality; PL, postnatal lethality; SD, strain-dependent; V, viability.

**TABLE 5:** Novel candidate genes for NMJ-related diseases in humans associated with defective signalling and synaptic transmission. All models are constitutive unless otherwise stated. Lethal phenotypes can have complete or incomplete penetrance. cKO, constitutive KO; PM, point-mutation.

Gene	Mouse model	Lethality^a^	References
Pre-synaptic terminal			
*Nrcam*	cKO by spontaneous B2 insertion; compound *Nrcam/Gars* and *Nrcam/Sh3tc2* mutants	PD (*Nrcam/Sh3tc2* KOs)	Morelli et al. (2017)
*Scn8a/Nav1.6*	cKO by spontaneous LINE1 insertion (*Med* mice); cKO by spontaneous PM (*m10J* mice); compound *Scn8a/Gars* mutants	SD and AD: V or PL	Searle (1962), Musarella et al. (2006), Caillol et al. (2012)
Synaptic cleft			
*Fgfbp1*	cKO	-	Taetzsch et al. (2017)
Post-synaptic terminal			
*Erbb2 and Erbb4*	Skeletal muscle-specific single *Erbb2* or double *Erbb2/Erbb4* KOs (*ACTA1-Cre*)	-	Leu et al. (2003), Escher et al. (2005)
*Gabpa*	Skeletal muscle-specific KO (*ACTA1-Cre*)	-	Jaworski et al. (2007), O’Leary et al. (2007)
*Nrg1/ARIA*	Heterozygous cKO	- (EL cKO)	Sandrock et al. (1997)

^a^ Key: AD, allele or model-dependent; EL, embryonic lethality; NL, neonatal lethality; PD, premature death; PNL, perinatal lethality; PL, postnatal lethality; SD, strain-dependent; V, viability.

**TABLE 6:** Novel candidate genes for NMJ-related diseases in humans associated with endocytosis, synaptic vesicle formation and neurotransmitter release. All models are constitutive unless otherwise stated. Lethal phenotypes can have complete or incomplete penetrance. cKO, constitutive KO; SMA, spinal muscle atrophy.

Gene	Mouse model	Lethality^a^	References
Pre-synaptic terminal			
*Dnajc5/Csp-⍺*	cKO	PD	Fernández-Chacón et al. (2004), Chandra et al. (2005), Ruiz et al. (2008), Rozas et al. (2012), Sharma et al. (2012), Zhang et al. (2012)
*Madd/Rab3 GEP*	cKO	SD: V or NL	Tanaka et al. (2001)
*Nbea*	cKO by random integration that in addition disturbs GH secretion causing dwarfism; cKO by gene trap	NL	Su et al. (2004), Medrihan et al. (2009), Nuytens et al. (2014)
*Pls3*	Overexpression in SMA models (Smn1^−/−^/Tg (SMN2))	PD	Ackermann et al. (2013), McGovern et al. (2015), Hosseinibarkooie et al. (2016)
*Rims1* and *Rims2*	cKO	NL	Schoch et al. (2002), Schoch et al. (2006)
*Stxbp5l/Tom2*	cKO	SD: V or PL	Geerts et al. (2015)

^a^ Key: AD, allele or model-dependent; EL, embryonic lethality; NL, neonatal lethality; PD, premature death; PNL, perinatal lethality; PL, postnatal lethality; SD, strain-dependent; V, viability.

**TABLE 7:** Novel candidate genes for NMJ-related diseases in humans associated with adhesion. All models are constitutive unless otherwise stated. Lethal phenotypes can have complete or incomplete penetrance. cKO, constitutive KO; MN, motor neuron; SMA, spinal muscle atrophy.

Gene	Mouse model	Lethality^a^	References
Synaptic cleft			
*Aplp2* and *App*	Skeletal muscle- (*Ckmm-Cre*), nervous system- (*Nes-Cre*), neuron-specific (*ChAT-Cre*) or constitutive *App* deletion in a *Aplp2* cKO background; humanized mutated and truncated *App* transgene in a *Aplp2* cKO background; constitutive deletion of the *Lrp4* transmembrane domain in an *App* cKO background	AD: V, PNL, PL or PD	Wang et al. (2005), Wang et al. (2007), Wang et al. (2009), Li et al. (2010), Choi et al. (2013)
*Fat1*	Skeletal muscle-specific (*Pax3-Cre*) hypomorph; skeletal muscle- (*Pax3-Cre*), MN- (*Olig2-Cre*), mesenchymal- (except cranial; *Prx1-Cre*), craniofacial mesenchymal-specific (*Wnt1-Cre*) or constitutive mutant lacking the transmembrane domain	AD: V, NL or PD	Caruso et al. (2013), Helmbacher (2018)
*Itgb1*	Neural crest- (*PLAT-Cre*), nervous system- (*Nes-Cre*) or skeletal muscle-specific (*ACTA1-Cre*) KOs	PD	Pietri et al. (2004), Schwander et al. (2004)

^a^ Key: AD, allele or model-dependent; EL, embryonic lethality; NL, neonatal lethality; PD, premature death; PNL, perinatal lethality; PL, postnatal lethality; SD, strain-dependent; V, viability.

**TABLE 8:** Novel candidate genes for NMJ-related diseases in humans associated with the DGC complex. All models are constitutive unless otherwise stated. Lethal phenotypes can have complete or incomplete penetrance. cKO, constitutive KO.

Gene	Mouse model	Lethality^a^	References
Post-synaptic terminal			
*Stnb1* and *Stnb2*	Single *Snta1*, *Sntb1*, or *Sntb2*, double *Snta1/Sntb2* or triple *Snta1/Sntb1/Sntb2* cKOs	-	Adams et al. (2000), Adams et al. (2004), Kim et al. (2019)
*Utrn*	Single *Utrn*, double *Utrn/Dmd* or triple *Utrn/Dmd/Dnta* cKOs	AD: V or PD	Deconinck et al. (1997a), Grady et al. (1997a), Deconinck et al. (1997b), Grady et al. (1997b), Grady et al. (1999), Grady et al. (2000)

^a^ Key: AD, allele or model-dependent; EL, embryonic lethality; NL, neonatal lethality; PD, premature death; PNL, perinatal lethality; PL, postnatal lethality; SD, strain-dependent; V, viability.

**TABLE 9:** Novel candidate genes for NMJ-related diseases in humans with unknown function. All models are constitutive unless otherwise stated. Lethal phenotypes can have complete or incomplete penetrance. cKO, constitutive KO.

Gene	Mouse model	Lethality^a^	References
Pre-synaptic terminal			
*Slc16a3/Mct4*	cKO	—	Bisetto et al. (2019)
*Snca*	Constitutive overexpression of mouse WT or human PD-associated A53T mutant form	AD: V or PD	van der Putten et al. (2000), Rieker et al. (2011)
Post-synaptic terminal?			
*Kalrn*	cKO or nervous system-specific KO (*Nes-Cre*)	SD: V or PD	Mandela et al. (2012)
Unknown			
*Gas7*	Truncated, lowly expressed and unstable hypomorph	-	Huang et al. (2012)
*Skor2*	cKO	PNL	Wang et al. (2011)

^a^ Key: AD, allele or model-dependent; EL, embryonic lethality; NL, neonatal lethality; PD, premature death; PNL,perinatal lethality; PL, postnatal lethality; SD, strain-dependent; V, viability.

**FIGURE 1:**
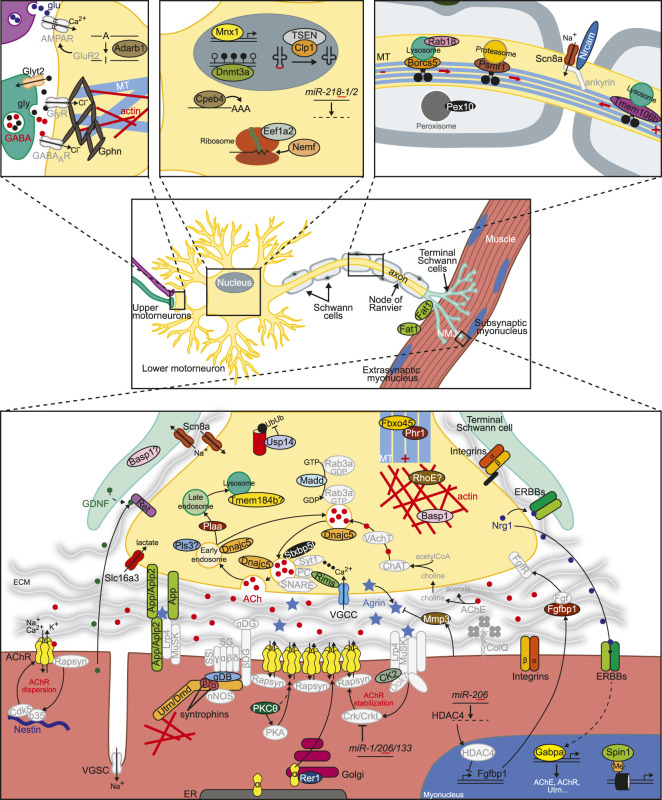
Factors involved in the regulation of NMJ formation, maintenance or function. Novel proteins or miRNAs whose dysfunction disturbs NMJ structure or function as described in this work appear in color. See Tables 1–9, Supplementary Table S3 and text for further details. Additional important factors need for normal NMJ biology are also included in grey. The basic NMJ template was obtained from Motifolio drawing toolkits (Motifolio Inc, Ellicott City, MD, USA). ECM, extracellular matrix; ER, endoplasmic reticulum; DG, dystroglycan; NMJ, neuromuscular junction; PC, priming complex (Munc13, Rab3/27); SG, sarcoglycan; SS, sarcospan; VGSC/VGCC, voltage-gated sodium/calcium channel.

We sought to characterize these 64 loci following the same classification used in the congenital myasthenic syndromes, which have been classically subdivided according to the specific location of the mutant protein as presynaptic, synaptic or postsynaptic (Tables 1–9, Supplementary Table S3 and Figure 1). In the next sections, we will briefly discuss the molecular mechanisms affected when these genes are not normally expressed. Of note, even if we originally aimed at finding genes that have a direct impact on NMJ morphology or function, our search has also uncovered genes whose malfunction lead to indirect defects in the NMJ, particularly when they affect motor neuron survival. In any case, the results obtained here help to expand the repertoire of genes to take into consideration when studying human pathologies.

### 2.1 Motor neuron identity, function and survival

Many of the novel candidates that could be indirectly associated with NMJ diseases are essential for MN development, function or viability (Table 1, Supplementary Table S3 and Figure 1). If MNs fail to reach their target muscles, NMJs obviously fail to form, while if innervation is correctly established but MNs later die, NMJs degenerate. Therefore, defects in the NMJ in the models described in this section are just secondary to defects in MNs. For example, in mice lacking the transcription factor Mnx1, MNs are generated, but their identity is not correctly specified because the interneuron identity program is not efficiently repressed (Arber et al., 1999; Thaler et al., 1999). This causes problems in axon projections and pathfinding, ultimately leading to the lack of innervation, and thus of NMJs, of certain muscles like the diaphragm (Arber et al., 1999). Thus, *Mnx1* KO mice are perinatal lethal due to their inability to breath. MNs are also born in *Slc6a5/Glyt2* mutant mice (Gomeza et al., 2003; Bogdanik et al., 2012). However, in the absence of this glycine transporter, essential to sustain prolonged glycinergic inhibitory transmission in the spinal cord, MNs exhibit increased excitability, that is, uncoordinated and excessive firing, together with an acceleration in postnatal NMJ maturation (Bogdanik et al., 2012). These molecular events result in a neuromotor deficiency that resembles human hyperekplexia, but unlike in humans, who can compensate the impairment in glycinergic transmission with increased GABAergic inhibition, *Slc6a5* mutant mice only survive for 2 weeks (Gomeza et al., 2003; Bogdanik et al., 2012). Glycinergic synaptic activity is also disturbed in *Gphn/Geph* mutant animals (Feng et al., 1998) given that this tubulin-binding protein anchors all glycine and several GABA_A_ receptor types to the microtubule network in the presynaptic MN. In these animals, MN survival and innervation patterns are affected during development, albeit differentially depending on the target muscle (Banks et al., 2005).

Reduced MN integrity and survival is additionally observed in mutant mice for *Adarb1/Adar2*, *Ret*, *Clp1* and *Pex10* for different reasons. In the absence Adarb1/Adar2, the mRNA editing needed for the expression of the correct isoform of the AMPA receptor subunit GluR2 does not take place, thus disturbing the properties of this channel and, ultimately, MN viability (Hideyama et al., 2010). Similarly, if the GDNF receptor Ret is missing, MNs cannot integrate GDNF inputs, which interferes with the proper organization and maturation of the presynaptic terminals and NMJs during development and regeneration, resulting in increased MN death (Baudet et al., 2008). The exact molecular mechanism is unknown, but it has been hypothesized that GDNF could activate Ret eliciting a signaling cascade within MNs, or it could be incorporated in the MN in a Ret-dependent manner and exert specific actions there (Baudet et al., 2008; Zahavi et al., 2015). In parallel, impaired function of Clp1, a component of the tSEN complex involved in RNA maturation, causes the accumulation of small aberrant RNA species that trigger p53-dependent apoptosis of MNs (Hanada et al., 2013). Finally, when the activity of the peroxisome-associated Pex10 protein is disturbed in SCs, these cells die (Hanson et al., 2014); not only do they accumulate toxic molecules, but they fail to synthesize essential molecules like plasmalogens, which are very abundant in myelin. Eventually, the lack of support from SCs has a negative impact in MN integrity and synaptic transmission (Hanson et al., 2014). Indeed, the role of SCs in NMJ development has proven fundamental. In the absence of SCs, even if axons appear defasciculated, MNs reach the target muscles and NMJs are initially formed. However, nerve terminals eventually retract and NMJs thus fail to be maintained (Lin et al., 2000). Curiously, NMJs can be preserved if muscle activity is blocked (Liu Y et al., 2019). This finding suggests that SCs counteract the presumable destabilizing activity muscle exerts in the developing NMJ, and which causes MN retraction (Liu K et al., 2019).

### 2.2 Channel-related

*Ducky* (Snell, 1955) and *Stargazer* (Noebels et al., 1990) mice are spontaneous mutants for the Cacna2d2 (Barclay et al., 2001) and Cacng2 (Letts et al., 1998) subunits of the voltage-gated calcium channels (VGCCs) required to conduct the presynaptic calcium influx along MN axons necessary for neurotransmitter release at the terminals (Table 2, Supplementary Table S3 and Figure 1). Both are epilepsy models that show spike-wave seizures and ataxia, among other features (Noebels et al., 1990; Barclay et al., 2001). In addition, mutations in these genes cause very mild phenotypes observed at the NMJ level, probably reflecting the existence of redundancy with other accessory channel subunits (Kaja et al., 2007).

The rest of the novel candidate genes causative or modifiers of NMJ-related disorders in this section are associated to various extents to postsynaptic AChRs (Table 2, Supplementary Table S3 and Figure 1). Here, we will briefly describe NMJ formation [recently reviewed in (Rodríguez Cruz et al., 2020)], highlighting the role of the candidate genes found in our search. During NMJ formation, small AChR clusters appear in the center of the muscle fibers in a nerve-independent process but that is mediated by Lrp4/MuSK/Dok7 and rapsyn at the muscle terminal (Lin et al., 2001; Yang et al., 2001; Okada et al., 2006; Weatherbee et al., 2006). Then, MNs innervate some of the prepatterned clusters, that get stabilized via neural-derived agrin signaling on Lrp4/MuSK/Dok7 at the postsynaptic terminal (McMahan, 1990; Glass et al., 1996; Okada et al., 2006; Kim et al., 2008; Zhang et al., 2008). Of note, MNs can organize *de novo* AChR clusters in the absence of prepatterned clusters (Lin et al., 2008; Liu et al., 2008). After agrin-induced stimulation, a series of events take place before AChR cluster stabilization, including phosphorylation of multiple MuSK residues, which function as docking sites for downstream signaling. Casein kinase II (CK2) holoenzyme is responsible for phosphorylating MuSK at its kinase inert domain (Cheusova et al., 2006). In the absence of the CK2 regulatory subunit Csnk2b, MuSK phosphorylation is disturbed, not only causing a negative impact on AChR cluster stabilization and postsynaptic structure, but also on synaptic transmission (Cheusova et al., 2006), leading to embryonic lethality in constitutive null mice (Buchou et al., 2003). AChR clustering and stability is controlled by phosphorylation of its different subunits as well. In this regard, agrin-induced tyrosine phosphorylation of the AChRβ subunit contributes to rapsyn association and receptor clustering (Borges et al., 2008). In parallel, PKCθ would be involved in the regulation of PKA-mediated phosphorylation of the AChRε subunit, and in the direct phosphorylation of AChRδ, two events that produce opposing effects: AChR stability or loss, respectively (Lanuza et al., 2010). This would explain the delay in postsynaptic maturation present in PKCθ deficient mice (Lanuza et al., 2010), which also show a delay in the early postnatal synapse elimination process to achieve monoinnervation by an unknown mechanism (Li et al., 2004). Whether the absence of PKCθ activity at the presynaptic terminal contributes to any of these two phenotypes cannot be discarded (Lanuza et al., 2010).

Agrin signaling terminates with its removal from the synaptic cleft. Mmp3 is a metalloprotease secreted by muscle that degrades the major components of the extracellular matrix and other proteins including agrin (VanSaun and Werle, 2000). In *Mmp3* deficient mice, agrin accumulates at the synaptic basal lamina, and further, it also appears abnormally located outside the central endplate area where AChR should only cluster (VanSaun et al., 2003). As a consequence, the structure of the postsynaptic membrane is greatly disturbed (VanSaun et al., 2003).

Not all prepatterned AChR clusters get stabilized. Those receptors that had integrated in the muscle membrane extrasynaptically do not receive agrin input and are dispersed through a Cdk5-dependent mechanism triggered by ACh (Lin et al., 2005). The intermediate filament protein nestin has been shown to play a role in this process (Mohseni et al., 2011; Yang et al., 2011). The model poses that unphosphorylated nestin and Cdk5 constitutively interact at the postsynaptic terminal. Upon ACh stimulation, p35 is recruited and activates Cdk5, which in turn phosphorylates nestin. This results in destabilization of the intermediate filament network, the release of active Cdk5 and AChR cluster dispersal (Yang et al., 2011). Thus, in *nestin* KO mice, Cdk5 activity is impaired, hindering extrasynaptic AChR dispersal (Mohseni et al., 2011; Yang et al., 2011), as well as disturbing proliferation in muscle, causing frequent spontaneous regeneration (Lindqvist et al., 2017). Of note, Cdk5 can be activated by a second mechanism that involves rapsyn and calpain (Chen et al., 2007).

Finally, the correct assembly of the AChR complexes at the membrane is undoubtedly essential for proper function. Rer1 is a Golgi-associated protein part of the quality control mechanism that retains unassembled or immature membrane protein complexes in the endoplasmic reticulum, ensuring only proper mature complexes are discharged [e.g., (Kaether et al., 2007)]. When Rer1 activity is compromised, unassembled AChR⍺ subunits -and no other subunit type-escape this quality control mechanism and accumulate in the postsynaptic membrane, or are bound for lysosome-mediated degradation, reducing the number of fully assembled AChRs at the muscle membrane (Valkova et al., 2011).

### 2.3 Regulation of gene expression at the transcript and protein levels. Proteostasis and autophagy

Dnmt3a is required for *de novo* DNA methylation, and is essential for the establishment of correct DNA methylation patterns during development. When this gene is removed specifically in the nervous system by means of *Nestin-Cre*-mediated recombination, NMJs are highly fragmented. Mutant mice exhibit problems in neuromuscular function and motor coordination in the absence of gross anatomical alterations in the cerebellum (Nguyen et al., 2007). The exact molecular mechanism is unknown but probably related with abnormal gene expression in MNs during development through disturbed methylation of specific targets (Nguyen et al., 2007). Indeed, even if spinal cord MNs appear normal in the mutants, *in vitro* it has been shown that Dnmt3a is required for MN differentiation (Ziller et al., 2018) (Table 3, Supplementary Table S3 and Figure 1). The potential contribution of skeletal muscle, considering *nestin* is also expressed in this tissue, cannot be discarded [(Mohseni et al., 2011; Yang et al., 2011; Lindqvist et al., 2017), see section *2.10* below].

In mice harboring a skeletal muscle-specific deletion of the chromatin reader *Spin1*, the expression of bHLH transcription factors involved in myogenesis, and by extension of their target genes, is altered during development and after birth (Greschik et al., 2017). Many of these targets are associated with the NMJ, such as Dok7, AChE, Colq and several AChR subunits. This not only results in severe morphological alterations of the postsynaptic muscle terminal, but also in defects in the presynaptic MN, including a reduced number of synaptic vesicles and the presence of vacuoles (Greschik et al., 2017) (Table 3, Supplementary Table S3 and Figure 1).

At the post-transcriptional regulation level, at least two micro-RNA (miRNA) families are associated with NMJ development (Table 3, Supplementary Table S3 and Figure 1). Being the most abundant MN miRNAs (Amin et al., 2015), the *miR-218* family is particularly important. In the absence of the *miR-218-1* and *miR-218-2* paralogs, hundreds of neuronal enriched genes from multiple molecular pathways are deregulated (Amin et al., 2015). This massive alteration in the transcriptome does not impact on MN development, but rather on MN maintenance and survival, as well as on NMJ formation (Amin et al., 2015). Of note, *miR-218* gene variants have recently been associated with amyotrophic lateral sclerosis (ALS) susceptibility (Reichenstein et al., 2019). The second miRNA family, *miR-1/206/133*, is involved in NMJ formation from the muscle side. The lack of these species alleviates the repression of their target gene *CRX*, which acts downstream the Lrp4/MuSK/Dok7 cascade and is involved in agrin-induced stabilization of forming AChR clusters (Rodríguez Cruz et al., 2020) through the recruitment of, at least, the Rho GTPase RAC1 (Klockner et al., 2022). Intriguingly, *miR-1/206/133* are dispensable for skeletal muscle development since only NMJ formation is altered in the triple KOs (Klockner et al., 2022). In contrast, if only *miR-206* is removed, NMJ development remains unperturbed, presumably due to compensation by other members of the family (Williams et al., 2009). Still, its target *Hdac4* is overexpressed, causing a negative effect on retrograde FGF signaling from muscle to its innervating neuron, where abnormalities are evident (Williams et al., 2009).

Further, Mbnl1 and Mbnl2 are RNA binding proteins involved in multiple aspects of RNA biology (Nakka et al., 2018). In double *Mbnl1* ^*−/−*^ */Mbnl2* ^*+/−*^ mutants, NMJs are greatly compromised presumably as a secondary effect to the severe defects observed in muscles, which develop a myotonic dystrophy (DM)-like phenotype (Lee et al., 2013). The mechanism by which NMJ deteriorate is probably related with a defect in the processing of one or multiple transcripts involved in NMJ development, maintenance and/or function. In particular, changes in alternative splicing and polyadenylation events are observed in the compound mutants (Lee et al., 2013) (Table 3, Supplementary Table S3 and Figure 1). In this sense, it is important to mention that alternative splicing has been shown to be a crucial layer in the regulation of NMJ development. For example, only neurons produce an alternatively spliced isoform of agrin called Z^+^ agrin in mammals, which is up to 1,000-fold more potent in AChR clustering than the Z^−^ isoforms (Ferns et al., 1993). Nova1 and Nova2 neuron-specific splicing factors regulate Z+ agrin splicing and thus, double mutant mice show defective morphology and function of the NMJ, and are paralyzed (Ruggiu et al., 2008). Remarkably, rescuing Z^+^ agrin expression restores some morphological defects, but physiologic MN activity remains impaired (Ruggiu et al., 2008). This would indicate that Nova factors target additional undefined transcripts that are essential for synaptic transmission, highlighting the importance of post-transcriptional regulatory mechanisms during NMJ development and function.

At the protein level, impaired translation in MNs seems to lie at the root of the denervation and neurodegeneration defects observed in *Eef1a2* (Shultz et al., 1982; Chambers et al., 1998; Khalyfa et al., 2001; Newbery et al., 2005; Doig et al., 2013) and *Nemf* (Martin et al., 2020) deficient animals, presumably due to the inability of these cells to maintain proper homeostasis (Table 3, Supplementary Table S3 and Figure 1). Remarkably, the core translation machinery component Eef1a2 is spontaneously mutated in the so-called *Wasted* mice (Shultz et al., 1982; Chambers et al., 1998), which develop an aggressive, early-onset form of neurodegeneration that can be prevented in constitutive heterozygotes (Griffiths et al., 2012), but not if reintroducing the gene specifically in muscle (Doig et al., 2013). In parallel, a KO and two-point mutation alleles that interfere with protein function have been described for *Nemf* (Martin et al., 2020). This rescue factor is part of the Ribosome-associated Quality Control RQC complex that resolves stalled ribosomes during translation (Martin et al., 2020). All alleles exhibit marked motor defects, with the KO being the most severe and showing complete preweaning lethality (Martin et al., 2020).

A number of novel candidates are involved in the degradation and recycling of proteins and organelles in the presynaptic MN terminal, either mediated by the endosomal-lysosomal system or the proteasome (Table 3, Supplementary Table S3 and Figure 1). Some of these genes play a role in the axonal transport of lysosomes and proteasomes through the microtubule network. That is the case of *Borcs5* (de Pace et al., 2020; Lüningschrör et al., 2020) and *Tmem106b* (Lüningschrör et al., 2020), in charge of the anterograde and retrograde transport of lysosomes (or lysosome-like particles), respectively. Mice defective for either gene show a reduction in the number of lysosomes in their axon terminals, causing impaired proteolysis and autophagy, and thus affecting the structure and function of axons (de Pace et al., 2020; Lüningschrör et al., 2020). In this same line, *in vitro* studies suggest that the absence of the small GTPase Rab18 could be associated with impaired lysosomal trafficking and autophagy in neurons (Nian et al., 2019). This would explain the *in vivo* phenotypes of *Rab18* KO animals, which accumulate neurofilament and microtubule proteins at presynaptic terminals (Carpanini et al., 2014). In parallel, when the adapter protein *Psmf1* is absent, the anterograde transport of proteasomes to MN terminals is impaired, eliciting proteotoxic stress followed by axonal degeneration and motor dysfunction (Liu Y et al., 2019; Minis et al., 2019).

When the process of endosomal-lysosomal-mediated degradation itself is altered in the MN terminal, problems in the NMJ arise as well (Table 3, Supplementary Table S3 and Figure 1). For example, the phospholipase A2 activator Plaa contributes to the sorting of ubiquitinated cargos from early to late endosomes or multivesicular bodies for their eventual degradation in lysosomes (Hall et al., 2017). When Plaa function is compromised, waste products accumulate at the NMJs and synaptic vesicle recycling -and neurotransmission by extension-is impaired (Hall et al., 2017). Presumably, Tmem184b would also participate in autophagosomal structure or clearance of cellular debris (Bhattacharya et al., 2016). Therefore, Tmem184b KO animals exhibit neurodegeneration due to the aberrant accumulation of recycled cargos at terminal synapses (Bhattacharya et al., 2016). Interestingly, via the same impaired mechanism, the absence of this protein has a neuroprotective role after injury since axon degeneration is delayed (Bhattacharya et al., 2016).

*Ataxia* mice or *axJ* mice, characterized by severe tremor and hindlimb paralysis, arose in 1965 (D’Amato and Hicks, 1965) as a spontaneous mutation in the *Usp14* gene (Wilson et al., 2002). Phenotypic analyses of this and other *Usp14* mutant models indicate that the lack of this deubiquitinating enzyme causes a defect in ubiquitin homeostasis at the presynaptic terminal with two immediate consequences (Table 3, Supplementary Table S3 and Figure 1). Firstly, proteasomal-mediated degradation of synaptic proteins involved in neurotransmitter release and NMJ shaping during postnatal development is impaired. Secondly, MNs are unable to recycle ubiquitin for additional protein targeting, on the other (Wilson et al., 2002; Chen et al., 2009; Chen et al., 2011; Bhattacharyya et al., 2012; Marshall et al., 2013). Proteasome-independent ubiquitin signaling through JNK (Vaden et al., 2015) is also affected in the mutants. All in all, these molecular malfunctions result in a wide array of phenotypic alterations that depend on the mouse strain and specific mutant allele. In all cases, though, NMJs are damaged.

### 2.4 Cytoskeleton dynamics

The neuronal RNA binding protein Cpeb4 is involved in mRNA polyadenylation, central in cellular metabolism. Cpeb4 null mice are mostly normal (Shin et al., 2016). However, a truncated version of the protein that only contains an unstructured low complexity domain has a dominant toxic effect in mice, triggering abnormal ribosomal biogenesis and function, as well as elevated levels of stress response genes, including *Drr1* (Shin et al., 2016). This gene encodes for an actin-bundling protein that interferes with neurite outgrowth (Schmidt M et al., 2011) and as a consequence, with the proper formation of NMJs due to the disruption of actin organization (Shin et al., 2016) (Table 4, Supplementary Table S3 and Figure 1).

Similarly, the calmodulin binding protein Basp1 would regulate actin dynamics at the presynaptic terminal, presumably by promoting subplasmalemmal actin accumulation (Frey et al., 2000). It would also control tSC-mediated synaptic growth and strength by an unknown mechanism (Frey et al., 2000). In any case, its function is very important for the organism since null mice exhibit a high postnatal mortality rate (Frey et al., 2000) (Table 4, Supplementary Table S3 and Figure 1).

Rnd proteins like Rnd3/RhoE are constitutively active members of the Rho GTPase family, and participate in multiple cellular functions, such as in the control of actin cytoskeleton dynamics (Guasch et al., 1998). In the absence of Rnd3/RhoE, mice display severe growth retardation, abnormal posture, motor impairments, and convulsions among other phenotypes (Mocholí et al., 2011). It has been proposed that Rnd3/RhoE could activate the RhoA/ROCK pathway in MNs, leading to the inactivation of cofilin, involved in actin depolymerization (Bamburg et al., 2021). This would generate MN axon growth or migration problems, as in the case of RhoE-defective hippocampal neurons (Peris et al., 2012). Alternatively, RhoE has been associated with skeletal muscle maturation, so a delay in myogenesis could have a negative impact on MN survival and/or establishment due to the lack of trophic factors (Mocholí et al., 2011) (Table 4, Supplementary Table S3 and Figure 1).

Phr1 is an atypical E3 ubiquitin-protein ligase that mediates ubiquitination of non-lysine residues on target proteins (Mabbitt et al., 2020). Its function is essential to stabilize the microtubule network in developing MNs, which in *Phr1* null mice present an abnormal organization with disorganized microtubules not oriented in the direction of axon growth and extending past the actin-rich growth cone at the tips (Lewcock et al., 2007). Molecularly, it has been hypothesized that Phr1 might function via regulation of MAPK signaling (Lewcock et al., 2007) and/or impaired ubiquitination of specific protein targets (Mabbitt et al., 2020), possibly in complex with Fbxo45 (Saiga et al., 2009) (Table 4, Supplementary Table S3 and Figure 1).

The last candidate in this section is Clip3/CLIPR-59, which is specifically expressed in MNs, where it localizes to the trans-Golgi network. When Clip3/CLIPR-59 activity is compromised, mice die perinatally due to altered innervation of the diaphragm, and consequently, to the inability to breath (Couesnon et al., 2013). While different muscle types are differentially affected, in general the number of nerve terminals is reduced in all of them, while no defects in axon guidance are observed in the mutants. This points to a specific role for Clip3/CLIPR-59 in NMJ maintenance and stability, probably related with protein/membrane trafficking or cytoskeleton remodeling (Couesnon et al., 2013). Given that this protein is involved in myoblast fusion *in vitro*, a potential role of the postsynaptic terminal in NMJ stabilization cannot be discarded (Sun et al., 2015) (Table 4, Supplementary Table S3 and Figure 1).

### 2.5 Defective signaling among tSCs, pre-, and post-synaptic terminals. Defective synaptic transmission

Throughout development and after birth, multiple signaling pathways are established between the different elements that compose the NMJ to ensure its proper formation, maintenance and function (Rodríguez Cruz et al., 2020) (Table 5, Supplementary Table S3 and Figure 1). One such pathway comprises the MN-secreted neuregulin1/Nrg1 signal, and its Erbb receptors located in skeletal muscle and tSCs (Belotti and Schaeffer, 2020). Not surprisingly, when this pathway is disturbed either under *Nrg1* haploinsufficiency (Sandrock et al., 1997) or by muscle-specific deletion of the Erbb2/Erbb4 receptors (Leu et al., 2003; Escher et al., 2005), postsynaptic integrity is compromised. Although controversial (Belotti and Schaeffer, 2020), defective Nrg1/Erbb signaling may affect the expression of subsynaptic genes like some AChR subunits via the GABP⍺ transcription factor, whose role in the process has also been questioned (Jaworski et al., 2007; O’Leary et al., 2007). Other possibilities include the destabilization of AChR clusters through impaired phosphorylation of the dystrophin-associated glycoprotein complex (DGC) component ⍺-dystrobrevin (Schmidt N et al., 2011), and the negative impact that impaired Nrg1/Erbb signaling would have on tSCs survival (Lin et al., 2000; Newbern and Birchmeier, 2010).

Additionally, FGF signaling from skeletal muscle to the innervating MN is essential for the formation and maintenance of NMJs (Rodríguez Cruz et al., 2020). For this reason, when the FGF carrier Fgfbp1 is not secreted to the synaptic cleft, NMJs mature slowly and deteriorate fast (Taetzsch et al., 2017). The exact FGF molecule and the presynaptic receptor that mediate in this process are unknown (Taetzsch et al., 2017). There is also the possibility that the lack of Fgfbp1 in mice has an indirect effect by eliciting other cellular changes that ultimately affect NMJ structural integrity (Taetzsch et al., 2017).

Scn8a/Nav1.6 is a voltage-gated sodium channel localized at presynaptic MNs. Several mutants have been described for this gene, with *Med* (*Motor Endplate Disease*) mice arising spontaneously in the 60s (Searle, 1962; Duchen et al., 1967; Burgess et al., 1995). These animals are characterized by a fatal form of progressive muscular weakness, and as early as in the 70s, it was already proposed that the disease was caused by progressive failure of neuromuscular transmission leading to muscle denervation (Duchen, 1970; Duchen and Stefani, 1971). Indeed, defective sodium currents in tSCs have been proposed to negatively affect the synthesis of growth factors necessary to support the development and function of NMJs (Musarella et al., 2006; Caillol et al., 2012). Defects in action potential generation in the presynaptic neuron can also contribute to the observed phenotypes, especially in the case of the *m10J* allele (Morelli et al., 2017). Mice carrying this mutation in heterozygosis do not show NMJ defects. However, they exhibit exacerbation of the phenotypes of *Gars* heterozygotes (Morelli et al., 2017), which constitute axonal models of the dominant axonal neuropathy Charcot-Marie-Tooth (CMT) disease and display defects in NMJ morphology and synaptic transmission on their own (Seburn et al., 2006; Achilli et al., 2009; Morelli et al., 2017). In these double *Gars/Scn8a* heterozygotes, reduced axon diameter and impaired sodium currents, presumably causing depolarization problems, synergize to produce more severe reductions in nerve conduction velocity and NMJ defects (Morelli et al., 2017). Similarly, mutants for the cell adhesion molecule Nrcam present normal NMJs, but the phenotypes of either demyelinating (*Sh3tc2* KO) or axonal (*Gars* heterozygotes) models of CMT are also exacerbated, possibly due to impaired generation of sodium currents at the presynaptic terminal (Morelli et al., 2017). It is important to mention that Nrcam binds ankyrins intracellularly, which in turn bind sodium channels like Scn8a, which appears mislocalized and functionally compromised in Nrcam mutants (Morelli et al., 2017).

### 2.6 Endocytosis, synaptic vesicle formation and neurotransmitter release

The release of ACh at the NMJ involves multiple steps such as ACh production, and synaptic vesicle formation, exocytosis and recycling. Defects in any of these steps could impair neurotransmission and cause motor problems (Table 6, Supplementary Table S3 and Figure 1). For example, the guanine nucleotide exchange factor Madd regulates the recycling between the GDP/GTP-bound forms of Rab3A, implicated in calcium-dependent neurotransmitter release (Tanaka et al., 2001). It is also probably required for the formation and trafficking of synaptic vesicles (Tanaka et al., 2001). Thus, in the absence of Madd, neurotransmission is severely impaired (Tanaka et al., 2001), as in the case of *Rims1/2* mutants (Schoch et al., 2006). Of note, these proteins act as scaffolds, interacting with several molecules at the active zone, including Rab3A, being essential for synaptic vesicle exocytosis (Schoch et al., 2002; Schoch et al., 2006). If Rims interact with the same molecules in central synapses and NMJs, then they would participate in the tethering of VGCCs to the active zones, enabling high calcium concentrations in the vicinity of synaptic vesicles to trigger exocytosis (Kiyonaka et al., 2007; Kaeser et al., 2011). Nbea is another scaffolding protein required for vesicle trafficking (Su et al., 2004). While synaptic transmission at the NMJ is blocked in *Nbea* null mice causing lethal paralysis in newborns (Su et al., 2004), the exact mechanism of action remains unknown: either the action potential that travels along MN axons fails to reach the presynaptic terminal, or it fails to trigger neurotransmitter release (Su et al., 2004). A possible contribution of impaired trafficking of specific proteins to the presynaptic terminal, as observed in neurons in the central nervous system, cannot be discarded (Medrihan et al., 2009). The role of Stxbp5l/Tom2 in NMJ biology is also not clear, especially in comparison with its paralog Stxbp5/Tom1, a well-known vesicle fusion inhibitor (Sakisaka et al., 2008). Due to its similarity, Stxbp5l/Tom2 may contribute to this function as well. In any case, mice deficient for this protein suggest that Stxbp5l/Tom2 may inhibit spontaneous ACh release to favor sustained secretion upon repetitive stimulation (Geerts et al., 2015).

In contrast, multiple studies have explored the function of Dnajc5/CSP-⍺ revealing that this chaperone impinges on many aspects of the neurotransmission process. Mice deficient for Dnajc5/CSP-⍺ develop a progressive sensorimotor disorder with progressive muscle weakness that leads to premature death (Fernández-Chacón et al., 2004). At the molecular level, these animals show an impairment in the maintenance of the synaptic release sites (Rozas et al., 2012), in the recycling process of the different components of the neurotransmitter release machinery (Fernández-Chacón et al., 2004; Rozas et al., 2012; Sharma et al., 2012; Zhang et al., 2012), as well as in the assembly of the SNARE complex (Chandra et al., 2005; Zhang et al., 2012), involved in vesicle release, proving Dnajc5/CSP-⍺ essential to maintain continued synaptic function (Fernández-Chacón et al., 2004; Chandra et al., 2005; Zhang et al., 2012). All these defects eventually cause activity-dependent neurodegeneration of the NMJ and defects in synaptic transmission (Fernández-Chacón et al., 2004; Ruiz et al., 2008). Reduced sensitivity to calcium-mediated neurotransmitter release may also contribute to the observed phenotypes (Ruiz et al., 2008).

Finally, whether the actin-bundling protein Pls3 participates significantly in neurotransmission remains a subject of debate. It was initially proposed that Pls3 overexpression had a significant beneficial effect upon the spinal muscle atrophy (SMA) phenotype (Oprea et al., 2008). However, findings in this regard from different studies are contradictory, surely because they are not homogeneous, the various laboratories using different overexpression models (tagged vs non-tagged Pls3 protein, random genomic insertion vs. targeted insertion at the ROSA26 locus) and mouse backgrounds (different strains; SMA model or not) (Ackermann et al., 2013; McGovern et al., 2015). All in all, it seems that if Pls3 does indeed have any beneficial role, the extent of protection would be related with the severity of the SMA model, with the most severe forms being irrecoverable due to multi-organ deficits (Ackermann et al., 2013). Protection would be through the rescue of endocytic processes that affect synaptic vesicle recycling in these models (Hosseinibarkooie et al., 2016).

### 2.7 Adhesion

The functions of the amyloid precursor protein APP go beyond its widely recognized association with Alzheimer’s disease. Indeed, the adhesion activities of APP and APP-like protein 2 (Aplp2) ensure the correct apposition between the pre- and postsynaptic terminals of the NMJ in the so-called transsynaptic APP interaction model (Wang et al., 2009). When this complex is disturbed in double *App/Aplp2* KO mice, the percentage of apposition is reduced and the presynaptic terminal morphology disturbed (Wang et al., 2005) (Table 7, Supplementary Table S3 and Figure 1). Also, APP regulates the presynaptic expression and activity of the high-affinity choline transporter CHT that mediates choline uptake from the synaptic cleft for ACh synthesis (Wang et al., 2007). These abnormalities are responsible for the defective synaptic transmission observed in the mutants (Wang et al., 2005). Further, the APP-Aplp2 complex interacts with Lrp4, likely to integrate agrin-induced AChR stabilization signals (Choi et al., 2013). The highly conserved APP C-terminal domain is indispensable for these functions (Li et al., 2010).

Adhesion plays a fundamental role during development as well. It has been shown that when integrin β1 is absent from tSCs, their migration along MN axons to the terminals is affected because this collagen receptor is required for the interaction between both cell types (Pietri et al., 2004) (Table 7, Supplementary Table S3 and Figure 1). Thus, tSC-specific functions during NMJ development are impeded (Pietri et al., 2004). If the expression is abolished in skeletal muscle, the interaction between the pre- and post-synaptic terminals is perturbed, impacting upon presynaptic differentiation and NMJ formation (Schwander et al., 2004). In contrast, no gross defects are observed in MN-specific mutants (Schwander et al., 2004).

Similarly, the concerted action of the protocadherin Fat1 in skeletal muscle, connective tissue and MNs during development, directly or indirectly controls the coordinated collective migration of myogenic precursors, as well as MN axonal growth and specification through gene expression regulation of MN-specific genes (Caruso et al., 2013; Helmbacher, 2018). When Fat1 activity is missing, the coupling of muscular and neuronal morphogenesis is altered, resulting in a wide array of abnormalities such as the misshaping and mispositioning of some muscle groups, poor motor performance, and defects in NMJ integrity (Caruso et al., 2013; Helmbacher, 2018) (Table 7, Supplementary Table S3 and Figure 1).

### 2.8 The dystrophin-associated glycoprotein complex DGC

The DGC is a multiprotein complex in charge of anchoring the cytoskeleton of muscle fibers to the extracellular matrix, thus being required for muscle stability (Ibraghimov-Beskrovnaya et al., 1992) (Table 8, Supplementary Table S3 and Figure 1). Utrophin (Utrn) and dystrophin (Dmd) are among its main components. While Utrn is located at the crests of the junctional folds where AChRs cluster, Dmd localizes at the base together with VGSCs (Flucher and Daniels, 1989; Sealock et al., 1991; Bewick et al., 1992). Not surprisingly, double *Utrn/Dmd* KOs present largely disassembled DGCs (Deconinck et al., 1997b; Grady et al., 1997b), unlike single mutants (Deconinck et al., 1997a; Grady et al., 1997a; Grady et al., 1997b) presumably due to the compensation between both proteins. As a consequence, AChR distribution is altered and postsynaptic folding almost inexistent, albeit with minimal effects in neurotransmission (Deconinck et al., 1997b; Grady et al., 1997b). ⍺-dystrobrevin (Dnta) is a soluble DGC component required to establish interactions with other proteins like the AChRs, so its absence in triple *Utrn/Dmd/Dnta* KOs worsens the phenotype (Grady et al., 1999; Grady et al., 2000). Syntrophins are also adapter proteins for the DGC, so in line with the previous models, single ⍺1-syntrophin (*Snta1*) (Adams et al., 2000) and, especially, double ⍺1-syntrophin/β2-syntrophin (*Snta1/Sntb2*) KOs (Adams et al., 2004) exhibit disturbed organization and stability of postsynaptic folds due to the disruption of the DGC. In contrast, single *Sntb1* or *Sntb2* (Adams et al., 2004) mutants have no overt phenotype, possibly because of compensation by ⍺1-syntrophin.

### 2.9 Unknown mechanisms

The pathogenic mechanisms that cause NMJ abnormalities in the last set of candidate genes are largely unknown and can only be hypothesized (Table 9, Supplementary Table S3 and Figure 1). For example, it has been shown that aged *Gas7*-deficient mice have fewer MNs than expected, which, in addition, show reduced terminal sprouting at NMJs (Huang et al., 2012). They also present changes in fiber type composition and size, but only in slow skeletal muscles (Huang et al., 2012). These abnormalities result in motor activity defects. Given its role in actin and tubulin polymerization *in vitro* (She et al., 2002; Akiyama et al., 2009), the lack of Gas7 could compromise the organization of cytoskeletal network of axon terminals. Alternatively, since it is also expressed in tSCs and skeletal muscle, where it localizes to the sarcomeres, NMJ abnormalities could result from a defect in tSCs supportive role on MNs, and/or to a muscle-autonomous problem in the contraction machinery (Huang et al., 2012).

Kalrn is a Rho GDP/GTP exchange factor that is expressed in multiple tissue-specific isoforms from the same gene, which complicates studies on its function. Null mice for most isoforms show marked abnormalities in the pre- and postsynaptic terminals of the NMJ, concomitant with reduced neuromuscular function (Mandela et al., 2012). These phenotypes are probably mostly related with the functions of the Kalrn9 and Kalrn12 isoforms in skeletal muscle given that defects are more pronounced in constitutive than in neuron-specific mutants (Mandela et al., 2012), where the Kalrn7 isoform is predominant (Penzes et al., 2000). Impaired clustering of receptors, channels and signaling proteins at the NMJ, or impaired sarcomeric formation in the muscle fibers may underlie the observed phenotypes (Mandela et al., 2012). Altered basal secretion of peptides from unspecified tissues could also contribute, while a role for Kalrn7 at the presynaptic terminal can also not be discarded (Mandela et al., 2012).

In one of the earliest publications regarding the function of the transcriptional co-repressor Skor2 in mammals (Wang et al., 2011), the authors mentioned that 90% of *Skor2* KO pups “die within 48 h of birth, probably owing to defective NMJ formation and respiration failure” but they did not elaborate further. This and follow-up publications revealed a role for Skor2 in the development of GABAergic neurons in the cerebellum (Wang et al., 2011; Nakatani et al., 2014) and midbrain (Kirjavainen et al., 2022), but the phenotypes in NMJs remain unexplored to date.

In contrast, a metabolic problem could explain the phenotypes of *Slc16a3/Mct4* deficient mice. This proton-linked transporter mediates the transport of metabolites like lactate or pyruvate across the plasma membrane. If its activity is disrupted in the presynaptic terminal, the motor unit is destabilized, and NMJs degenerate among other phenotypes (Bisetto et al., 2019). If metabolic pathways responsible for ATP production are disturbed in the absence of *Slc16a3/Mct4*, there would not be enough energy for neurotransmitter release and NMJ would eventually deteriorate (Bisetto et al., 2019). Surprisingly, even if mutant muscles accumulate high amounts of lactate, they are morphologically and functionally unperturbed (Bisetto et al., 2019).

Finally, the most predominant hallmark of Parkinson’s Disease (PD) is the presence of the so-called Lewy bodies, protein aggregates that contain ⍺-synuclein (Snca), among other proteins, and that accumulate within neurons disturbing cellular homeostasis and eventually causing cell death (Srinivasan et al., 2021). When trying to model PD in mice, several authors have overexpressed mouse *Snca* (Rieker et al., 2011) or a mutant form of human *SNCA* described in PD patients (van der Putten et al., 2000). In these models, NMJs degenerate and mice display motor abnormalities, being the human mutant overexpression model the most severe (van der Putten et al., 2000). They also manifest ubiquitin immunopathology, especially when overexpressing the mouse form Rieker et al. (2011). Since Snca has been shown to be involved in the regulation of synaptic vesicle trafficking [e.g., (Burré et al., 2010)], these defects could be related with disturbed recycling of synaptic vesicles and/or with the inhibition of the proteasome catalytic activity due to ubiquitination overload, thus causing a problem in synapse maintenance (van der Putten et al., 2000; Rieker et al., 2011).

### 2.10 Other considerations

*Lgals1* encodes for galectin-1, which binds numerous complex carbohydrates and, among other functions, is involved in the removal of nerve terminals at the NMJ after denervation (Plachta et al., 2007) directly via the Sema3a pathway (Quintá et al., 2014), and indirectly through the recruitment of macrophages (Gaudet et al., 2009). However, *Lgals1* constitutive KO mice do not have a defect in NMJ morphology despite having such annotation in the MGI database. Similarly, even if Utp14b, essential for spermatogenesis (Beamer et al., 1988) is associated with “failure in neuromuscular synapse postsynaptic differentiation” at MGI, the referenced paper is unrelated (Beamer et al., 1988), and we failed to find any other relationship with the NMJ in the literature.

Interestingly, Nmf67 mice present increased fragmentation of the postsynaptic terminal and premature death, among other defects in the heart and eyes. However, the gene altered in this chemically induced model is yet to be identified https://www.jax.org/strain/004472.

Caution should be taken when using compound models that involve previously generated mutant alleles even if they had extensively been used in the literature before. For example, the *Mnx1*-*Cre* line is a very common resource to drive Cre-mediated recombination in motor neurons (Yang et al., 2001). *Mnx1*-*Cre* heterozygotes are fully viable and fertile, but may have reduced endplate and muscle fiber sizes (Ackermann et al., 2013). Thus, *Mnx1* happloinsufficiency may have a phenotype on its own that should be taken into account especially if studying NMJs. Further, Cre-mediated recombination in lines driving tissue-specific *Cre* expression might not be very efficient or even inaccurate, as in the case of the *Nes*-*Cre* line used for generating neural-specific *Dnmt3a* KOs, in which recombination is observed in non-neural tissues as well (Nguyen et al., 2007). Indeed, *nestin* is also expressed in skeletal muscle where it would participate in the dispersal of extrasynaptic AChRs (Mohseni et al., 2011; Yang et al., 2011; Lindqvist et al., 2017), as mentioned above. In parallel, when random integration is used to generate transgenic animals, only in few occasions the exact genomic location where the transgene has integrated is determined. However, this could be an essential factor since the transgene might have inactivated an endogenous gene influencing the phenotypic outcome of the model, as shown for some of the most highly used transgenic mouse lines (Goodwin et al., 2019). For example, a profilin transgene containing a mutation associated with ALS disrupts the *Mdga2* gene, involved in cell-cell interactions, according to The Jackson Laboratory (https://www.jax.org/strain/030568). However, the authors of the model, which leads to abnormal NMJ morphology among other phenotypes, never stated this in the original publication (Fil et al., 2017) and thus, the potential contribution of Mdga2 to the observed phenotypes is currently unknown. Even if homologous recombination is used to target a specific locus, the potential scars or exogenous sequences introduced in the process could have unexpected consequences. For example, the three different mutant models available for the myogenic regulatory factor *Mrf4*, still harbor the *pgk-neomicine* resistance cassette that was used for homologous recombination in all cases (Olson et al., 1996). Surprisingly, these models exhibit diverse phenotypes that range from complete viability to lethality at birth. This variability is explained by the fact that the resistance cassette affects the expression of the neighboring *Myf5* gene (Vicente-García et al., *unpublished results*) to various extents, as hypothesized (Olson et al., 1996), making these models compound *Mrf4:Myf5* mutants. All in all, the above-mentioned factors should therefore always be taken into account when drawing conclusions from our experiments.

Finally, the list of novel candidate genes for NMJ-associated diseases described here is not exhaustive, as it relies on the manual curation of the MGI database that needs to continuously scan the increasingly vast literature. Also, the fact that we use very specific terms to retrieve models with deficient NMJs could also contribute to some essential genes being overlooked: the approach described here is thus highly selective at the cost of a potential high number of false negatives. For example, key genes known to have a direct impact in NMJ development, maintenance or function, like some genes of the Wnt signaling pathway (e.g., Vangl2: Boëx et al., 2022; Messéant et al., 2017; Wls; Shen et al., 2018), are missing from the search results. Further, null mice for the MuSK binding protein Biglycan present defects in synapse stability, which leads to morphological abnormalities (Amenta et al., 2012). However, this phenotype is still not included in the database. Likewise, while *Sam68* KO mice are included in the database, their only phenotypic annotations are associated with the role of this RNA-binding protein in bone marrow mesenchymal cell differentiation (Richard et al., 2005), while its function in maintaining NMJ integrity (de Paola et al., 2020) still awaits incorporation. In any case, our approach has indeed enabled the unveiling of mostly understudied genes in the context of NMJ biology in the expectation that they would be recognized in the field.

## 3 Factors to consider in the diagnosis and treatment of NMJ-related diseases

The mouse is the most commonly used model organism for the study of NMJ-related diseases. However, it must be taken into account that NMJs exhibit species-specific characteristics, with many differences existing between mice and humans. Thus, the mouse may not the most suitable model organism in all cases. Further, differences also exist between muscle types or even sexes, which are factors that need to be considered in the study of NMJ disorders as well.

### 3.1 Species-specific characteristics of the NMJ

The physiology of NMJs has been extensively studied in vertebrates and invertebrates (e.g., Couteaux, 1955 and reviewed in Clarac and Pearlstein, 2007). Although the invertebrate nervous system is considered “simple”, many of the findings obtained in invertebrates have been extrapolated to vertebrates. For example, studies on the crayfish (Wojtowicz et al., 1994) established the basis and principles for analyses in other invertebrates, such as *Drosophila* (Lnenicka, 2020), and later, in more complex organisms (Ackermann et al., 2015).

Even if mammalian NMJs have been more deeply characterized, little is known about the differences between the NMJs of different species even if these differences were already apparent in the 1950s (Couteaux, 1955), especially in comparison to humans. To date, the mouse is the main model organism used both for the study of NMJs and for the development of disease models that affect them. However, in many cases, the results obtained in these models have not been successfully translated to humans, as in ALS models (Clerc et al., 2016). This may be due to differences in the morphology or functionality of NMJs between both species. Indeed, human NMJs are much smaller than in the mouse, and they also have much thinner pre-synaptic axons with more rudimentary nerve endings and a different post-synaptic conformation (Jones et al., 2017). In addition, human NMJs vary from those in mice and other species in the so-called safety factor, originally defined as the excess of released ACh to ensure that neuromuscular transmission takes place (Wood and Slater, 2001). Precisely, morphological diversity may underlie differences in the safety factor between species. In general, it seems that the safety factor in humans is lower than in mice (Wood and Slater, 2001). Of note, variation or reduction in the safety factor is associated with different pathological conditions (Wood and Slater, 2001), such as congenital myasthenic syndromes (Engel et al., 2015), or myasthenia gravis (Plomp et al., 2017).

Thus, to better understand the form and function of the NMJ in human health and disease, it is important to identify animal models that more closely resemble human NMJs. Given the clear differences identified between humans and mice (Jones et al., 2017), larger animal models could provide more suitable, albeit probably more impractical, alternatives.

Boehm and others have recently compared the morphology of the NMJs of four different hindlimb muscles in six mammalian species (mouse, cat, dog, pig, sheep, and human) (Boehm et al., 2020). Using the NMJ-morph tool (Jones et al., 2016), they performed a deep qualitative and quantitative characterization. In the qualitative analysis, they observed that mouse NMJs have a characteristic, so-called “pretzel” morphology, while human NMJs were surprisingly small and nummular (coin-shaped) in morphology (Jones et al., 2017). Strikingly, the morphology of pig and sheep NMJs was very similar to each other, and the closest to that of humans.

In the quantitative analysis, a total of 21 pre- and post-synaptic variables were measured, including nerve terminal areas, the number terminal branches, AChR and endplate areas, and the degree of fragmentation of the endplate, among others (Table 10). Most of these variables were significantly larger in mice and dogs compared to humans. In contrast, these parameters attained the lowest values in cats. When considering overall size, cats and humans had strikingly small NMJs, while dogs presented the largest ones. As in the qualitative analysis, sheep and pig NMJs showed the highest similarity to human NMJs, especially sheep, with 19 of the 21 parameters analyzed showing no statistically significant differences (18/21 in the case of pigs) (Boehm et al., 2020).

**TABLE 10:** Characteristics of NMJs in different mammalian species taking the human as reference. Key: (−) lower, (+) greater, (++) two times larger, (+++) three or more times larger values than in humans. Significance levels: **p < 0.01, ****p < 0.0001. Original data from (Boehm et al., 2020).

Parameter	Mouse	Cat	Dog	Sheep	Pig
Presynaptic terminal complexity	+	+**	+**	+	+
Average area of postsynaptic AChR clusters	+++****	+	++****	-	+
NMJ fragmentation	-	-	-	+	-
Overlap between pre- and postsynaptic terminals	+	-	+	+	+
NMJ overall size	++****	-	++****	+	+

This study disproves the theory that NMJ size is inversely correlated with body mass, a theory proposed over 3 decades ago (Balice-Gordon and Lichtman, 1990). Indeed, it was hypothesized that the small size of human NMJs compared to mouse ones was due to the difference in size between both species. However, now according to Boehm et al. (2020), this is not the case, as rats or cats, larger animals than mice, have equal and smaller NMJs relative to humans, respectively.

To date, this is the only study that has carried out in-depth comparative analyses of NMJs in different mammalian species, highlighting the limitations of using the mouse as a model. Its main drawback, though, is that it only considers hindlimb muscles, so it could be possible that for other muscles, the animals with the greatest similarity to humans are not sheep. It would be therefore important to extend this work to more muscle types of diverse developmental origin.

In addition to differences in morphology and size of the pre- and post-synaptic components, differences can also be observed in tSCs (Santosa et al., 2018). In comparison with mice, despite having a similar number of tSCs per NMJ, human tSCs are significantly smaller, covering less AChR synaptic area, but extending extrasynaptically (Alhindi et al., 2021), at least in the *peroneus brevis* hindlimb muscle. These features, whereas being normal in human tSCs, have been considered as “pathological” in mice, as they have been described in mouse models of ALS and in denervation experiments (Kang et al., 2014; Carrasco et al., 2016). This highlights the importance of understanding the structure and function of all components of the NMJ in each species, in order to be able to efficiently translate scientific findings to humans.

Furthermore, the above-mentioned morphological differences among the NMJs of the diverse species are accompanied by distinct functionalities, as exemplified by their differential response to aging. Numerous studies based on work with mice suggest that mammalian NMJs are unstable with age (Valdez et al., 2010; Willadt et al., 2016), but little is known about what happens in humans. Indeed, during aging, mouse NMJs suffer post-synaptic fragmentation, pre-synaptic axon diameter decreases, partially innervated NMJs appear, and a reduction of AChR density takes place (Valdez et al., 2010). In humans, however, it has been shown that these events do not occur: there is only a slight increase in the diameter of pre-synaptic axons, with no signs of remodeling or fragmentation of the NMJs, which remain fairly stable throughout (Jones et al., 2017), in contrast to other mammalian species.

### 3.2 The impact of muscle properties and sex on NMJ morphology and functionality

For years it has been hypothesized that there was a positive correlation between NMJ size and muscle fiber diameter (Balice-Gordon and Lichtman, 1990). However, a recent study has failed to find any relationship between the morphology of the NMJ and the diameter of the fiber it innervates (Boehm et al., 2020), suggesting that fiber size is not a determining factor in NMJ morphology.

In contrast, fiber type does play a role: in general, NMJs innervating fast type II (a, x, and b) muscle fibers appear to be more elaborate, with a greater number of branches and a larger area than those innervating slow type I fibers (Sieck and Prakash, 1997; Mejia Maza et al., 2021). Further, NMJs innervating type II fibers show less overlap between the pre- and post-synaptic terminals, leading to increased susceptibility to neuromuscular failure and subsequent fatigue, while NMJs innervating type I fibers are more resistant to failure and less prone to fatigue (Sieck and Prakash, 1997). However, in the mouse this is not always the case. For example, in the hindlimb *soleus* muscle*,* slow type I fibers have larger NMJs than their fast Ia counterparts. This could be due to the fact that the *soleus* is a major postural muscle, thus subject to constant physical activity and therefore more neurotransmission, which could lead to the expansion of both pre- and post-synaptic structures. This adaptation would allow the NMJ to store more ACh and express a greater number of receptors, which would help delay the onset of neuromuscular failure and fatigue (Deschenes et al., 2013).

In addition to the differences associated with the type of fiber that make up the muscles, it appears that the physiological state of the muscle may influence the morphology of the NMJ that innervates it. Numerous studies have shown that aging produces morphological changes in NMJs (Deschenes et al., 2013; Willadt et al., 2016; Burke et al., 2021; Dobrowolny et al., 2021). For example, in mouse *extensor digitorum longus*, *gastrocnemius* and *adductor longus* limb muscles, there is an increase in endplate area as well as an increased number of AChR clusters at 34 months of age (Burke et al., 2021). Exercise and caloric restriction are additional factors that produce alterations in NMJs and they can even prevent their deterioration with aging (Valdez et al., 2010).

Furthermore, the function that a muscle performs could also influence NMJ morphology. For example, the *splenius capitis* cranial muscle in the rat has higher ratios of endplate size to muscle fiber volume than the *quadriceps* limb muscle, with which it has important functional and structural differences, even if both are considered fast muscles (Pfister and Zenker, 1984). Indeed, cranial muscles perform very different functions to other muscles in the body, such as the laryngeal muscles, which regulate the shape and tension of the vocal cords that mediate the control of phonation, vocalization, breathing, and swallowing. They have a fast shortening speed, high active tension, and resistance to fatigue. Their functional and structural differences with the muscles of the limbs is such that they remain intact or only slightly affected in some degenerative neuromuscular diseases: their specific characteristics may thus confer resistance to certain pathologies (Feng et al., 2012). Feng and others compared the structure and morphology of NMJs of laryngeal muscles with those of hindlimb muscles in rats (Feng et al., 2012). They analyzed 3 laryngeal muscles (*laryngeal thyroarytenoid* (TA), *cricothyroid* (CT), and *posterior cricoarytenoid* (PCA)) and 2 hindlimb muscles (*soleus* (SOL) and *extensor digitorum longus* (EDL)) and found significant differences between them. In particular, in laryngeal muscles, NMJs were more fragmented the higher the proportion of fast fibers in the muscle, with the PCA and TA showing the highest degree of fragmentation. This relationship does not occur in hindlimb muscles, which, in contrast, present the largest NMJs (Feng et al., 2012). Furthermore, they confirmed that, as in humans, polyinnervation is also present in rat laryngeal muscles, a finding that had only been previously observed in cranial muscles (Feng et al., 2012).

Given the remarkable differences among the NMJs of diverse muscles within the same organism, it stands to reason that there may also be differences between the NMJs of females and males of the same species. This would not be surprising considering the numerous studies that have shown sex-specific characteristics of skeletal muscle, including differential fiber type composition or size, and contraction strength (Staron et al., 2000; Davegårdh et al., 2019; O’Reilly et al., 2021). However, little is known about the possible morphological differences between NMJs of both sexes, since there are few works that directly addresses this issue, to the best of our knowledge. One such study found that in the case of rat EDL, SOL, and PL hindlimb muscles, there are no significant differences in the NMJs of females and males, although in all three muscles fiber sizes differ, being male fibers larger than those in females (Deschenes et al., 2013). This is in contrast to one of the laryngeal muscles, the TA, in which females have more fragmented NMJs than males (Feng et al., 2012), which might contribute to sexual dimorphism in the voice (Lenell and Johnson, 2017). These variations in NMJs could be the reason why certain muscle diseases affect both sexes differently, as in the case of spasmodic dysphonia, characterized by involuntary movements and inconsistent contraction of the laryngeal muscles, and mainly affecting females (Feng et al., 2012). However, further comparative studies are needed to determine whether differences in the NMJs of other muscles exist, which may allow us to understand the differential involvement of both sexes in some pathologies.

## 4 Conclusion

NMJs are complex structures responsible for the translation of nervous inputs into functional motor outcomes. Malfunction in any of its components -MNs, skeletal muscle or tSCs-result in defects in the development, maintenance, activity and integrity of the NMJ. In humans, 42 NMJ-specific disorders, the congenital myasthenic syndromes, have been described as indicated in the Gene Table of Neuromuscular Disorders (www.musclegenetable.fr), a specialized database that collects all known neuromuscular diseases and their causative genes, if known (Dubowitz, 1991). However, “pure” MN or skeletal muscle dystrophies can also lead to NMJ deficiencies. In addition, the multitude of mouse mutant lines generated by the scientific community has taught us that there are probably many more genes essential for NMJ biology. Several reasons could explain the fact that they have not been associated with a disease in humans. First, their function could be so essential that their absence is incompatible with life and thus, it is not possible to find patients carrying mutations in these genes. Second, the function of these genes may not be as essential in humans due to the existence of redundant or compensatory mechanisms, or simply because their functions in mice and humans have evolved divergently. Therefore, their malfunction would not cause significant phenotypes in humans. Third, many genes are pleiotropic and therefore control several traits even in different organs or at different developmental time points. Deficiencies in one such genes could cause earlier or more serious phenotypes than defects in the NMJ, especially if they do not affect gross motor skills. In this scenario, abnormalities in the NMJ would probably be missed unless specifically addressed. Or fourth, they have not been associated with a human disease to date. In any case, we provide here a list of novel candidate genes that should be considered in the study of the pathophysiological mechanisms of diseases that affect the NMJ and their potential treatments. This list is not exhaustive since it relies on manually curated data available at the MGI database. Thus, attention should be paid in the future for novel annotations and references that could expand the repertoire of potential candidate genes associated with NMJ-related diseases.

The mouse is the most commonly used model for the study of NMJ-related diseases, however, as described in this review, it may not always be the best choice. Numerous studies have shown that there are important differences between mouse and human NMJs, which may explain the failure to translate the results obtained with this animal to the clinic. In addition, we have seen how larger mammalian models, such as sheep or pigs, could provide a better model, closer to humans, for the study of these pathologies. Nevertheless, more studies are needed to gain a detailed understanding of the features of the NMJs of small and large mammals so that we have a model as close as possible to humans. It is also important to bear in mind that muscle properties and even biological sex can influence NMJ morphology and function. For example, it has been shown that the type of fiber that innervates the NMJ affects its size. However, comparative analyses that take into account these factors are very limited, especially in the case of sex-specific differences. Thus, the field would greatly benefit from more studies in this regard in order to develop efficient treatments for NMJ-related diseases in human.

## References

[B1] Achilli F. Bros-Facer V. Williams H. P. Banks G. T. AlQatari M. Chia R. (2009). An ENU-induced mutation in mouse glycyl-tRNA synthetase (GARS) causes peripheral sensory and motor phenotypes creating a model of Charcot-Marie-Tooth type 2D peripheral neuropathy. Dis. Model Mech. 2, 359–373. 10.1242/dmm.002527 19470612 PMC2707104

[B2] Ackermann B. Kröber S. Torres-Benito L. Borgmann A. Peters M. Hosseini Barkooie S. M. (2013). Plastin 3 ameliorates spinal muscular atrophy via delayed axon pruning and improves neuromuscular junction functionality. Hum. Mol. Genet. 22, 1328–1347. 10.1093/hmg/dds540 23263861

[B3] Ackermann F. Waites C. L. Garner C. C. (2015). Presynaptic active zones in invertebrates and vertebrates. EMBO Rep. 16, 923–938. 10.15252/embr.201540434 26160654 PMC4552486

[B4] Adams M. E. Kramarcy N. Fukuda T. Engel A. G. Sealock R. Froehner S. C. (2004). Structural abnormalities at neuromuscular synapses lacking multiple syntrophin isoforms. J. Neurosci. 24, 10302–10309. 10.1523/JNEUROSCI.3408-04.2004 15548643 PMC6730292

[B5] Adams M. E. Kramarcy N. Krall S. P. Rossi S. G. Rotundo R. L. Sealock R. (2000). Absence of alpha-syntrophin leads to structurally aberrant neuromuscular synapses deficient in utrophin. J. Cell Biol. 150, 1385–1398. 10.1083/jcb.150.6.1385 10995443 PMC2150701

[B6] Akiyama H. Gotoh A. Shin R.-W. Koga T. Ohashi T. Sakamoto W. (2009). A novel role for hGas7b in microtubular maintenance: Possible implication in tau-associated pathology in alzheimer disease. J. Biol. Chem. 284, 32695–32699. 10.1074/jbc.M109.035998 19801671 PMC2781685

[B7] Alhindi A. Boehm I. Forsythe R. O. Miller J. Skipworth R. J. E. Simpson H. (2021). Terminal Schwann cells at the human neuromuscular junction. Brain Commun. 3, fcab081. 10.1093/braincomms/fcab081 33977269 PMC8093923

[B8] Amenta A. R. Creely H. E. Mercado M. L. Hagiwara H. McKechnie B. A. Lechner B. E. (2012). Biglycan is an extracellular MuSK binding protein important for synapse stability. J. Neurosci. 32, 2324–2334. 10.1523/JNEUROSCI.4610-11.2012 22396407 PMC3313673

[B9] Amin N. D. Bai G. Klug J. R. Bonanomi D. Pankratz M. T. Gifford W. D. (2015). Loss of motoneuron-specific microRNA-218 causes systemic neuromuscular failure. Science 350, 1525–1529. 10.1126/science.aad2509 26680198 PMC4913787

[B10] Arber S. Han B. Mendelsohn M. Smith M. Jessell T. M. Sockanathan S. (1999). Requirement for the homeobox gene Hb9 in the consolidation of motor neuron identity. Neuron 23, 659–674. 10.1016/s0896-6273(01)80026-x 10482234

[B11] Bailey A. G. Bloch E. C. (1987). Malignant hyperthermia in a three-month-old American Indian infant. Anesth. Analg. 66, 1043–1045. 10.1213/00000539-198710000-00028 3631569

[B12] Balice-Gordon R. J. Lichtman J. W. (1990). *In vivo* visualization of the growth of pre- and postsynaptic elements of neuromuscular junctions in the mouse. J. Neurosci. 10, 894–908. 10.1523/JNEUROSCI.10-03-00894.1990 2156964 PMC6570117

[B13] Bamburg J. R. Minamide L. S. Wiggan O. Tahtamouni L. H. Kuhn T. B. (2021). Cofilin and actin dynamics: Multiple modes of regulation and their impacts in neuronal development and degeneration. Cells 10, 2726. 10.3390/cells10102726 34685706 PMC8534876

[B14] Banks G. B. Kanjhan R. Wiese S. Kneussel M. Wong L. M. O’Sullivan G. (2005). Glycinergic and GABAergic synaptic activity differentially regulate motoneuron survival and skeletal muscle innervation. J. Neurosci. 25, 1249–1259. 10.1523/JNEUROSCI.1786-04.2005 15689563 PMC6725962

[B15] Barclay J. Balaguero N. Mione M. Ackerman S. L. Letts V. A. Brodbeck J. (2001). Ducky mouse phenotype of epilepsy and ataxia is associated with mutations in the Cacna2d2 gene and decreased calcium channel current in cerebellar Purkinje cells. J. Neurosci. 21, 6095–6104. 10.1523/JNEUROSCI.21-16-06095.2001 11487633 PMC6763162

[B16] Baudet C. Pozas E. Adameyko I. Andersson E. Ericson J. Ernfors P. (2008). Retrograde signaling onto Ret during motor nerve terminal maturation. J. Neurosci. 28, 963–975. 10.1523/JNEUROSCI.4489-07.2008 18216204 PMC6671002

[B17] Beamer W. G. Cunliffe-Beamer T. L. Shultz K. L. Langley S. H. Roderick T. H. (1988). Juvenile spermatogonial depletion (jsd): A genetic defect of germ cell proliferation of male mice. Biol. Reprod. 38, 899–908. 10.1095/biolreprod38.4.899 3401545

[B18] Belotti E. Schaeffer L. (2020). Regulation of gene expression at the neuromuscular junction. Neurosci. Lett. 735, 135163. 10.1016/j.neulet.2020.135163 32553805

[B19] Bewick G. S. Nicholson L. Young C. O’Donnell E. Slater C. R. (1992). Different distributions of dystrophin and related proteins at nerve-muscle junctions. Neuroreport 3, 857–860. 10.1097/00001756-199210000-00009 1421088

[B20] Bhattacharya M. R. C. Geisler S. Pittman S. K. Doan R. A. Weihl C. C. Milbrandt J. (2016). TMEM184b promotes axon degeneration and neuromuscular junction maintenance. J. Neurosci. 36, 4681–4689. 10.1523/JNEUROSCI.2893-15.2016 27122027 PMC4846669

[B21] Bhattacharyya B. J. Wilson S. M. Jung H. Miller R. J. (2012). Altered neurotransmitter release machinery in mice deficient for the deubiquitinating enzyme Usp14. Am. J. Physiol. Cell Physiol. 302, C698–C708. 10.1152/ajpcell.00326.2010 22075695 PMC3287356

[B22] Birks R. Huxley H. E. Katz B. (1960). The fine structure of the neuromuscular junction of the frog. J. Physiol. 150, 134–144. 10.1113/jphysiol.1960.sp006378 13800900 PMC1363152

[B23] Bisetto S. Wright M. C. Nowak R. A. Lepore A. C. Khurana T. S. Loro E. (2019). New insights into the lactate shuttle: Role of MCT4 in the modulation of the exercise capacity. iScience 22, 507–518. 10.1016/j.isci.2019.11.041 31837519 PMC6920289

[B24] Bloom A. J. Miller B. R. Sanes J. R. DiAntonio A. (2007). The requirement for Phr1 in CNS axon tract formation reveals the corticostriatal boundary as a choice point for cortical axons. Genes Dev. 21, 2593–2606. 10.1101/gad.1592107 17901218 PMC2000324

[B25] Boehm I. Alhindi A. Leite A. S. Logie C. Gibbs A. Murray O. (2020). Comparative anatomy of the mammalian neuromuscular junction. J. Anat. 237, 827–836. 10.1111/joa.13260 32573802 PMC7542190

[B26] Boëx M. Cottin S. Halliez M. Bauché S. Buon C. Sans N. (2022). The cell polarity protein Vangl2 in the muscle shapes the neuromuscular synapse by binding to and regulating the tyrosine kinase MuSK. Sci. Signal 15, eabg4982. 10.1126/scisignal.abg4982 35580169

[B27] Bogdanik L. P. Chapman H. D. Miers K. E. Serreze D. v. Burgess R. W. (2012). A MusD retrotransposon insertion in the mouse Slc6a5 gene causes alterations in neuromuscular junction maturation and behavioral phenotypes. PLoS One 7, e30217. 10.1371/journal.pone.0030217 22272310 PMC3260239

[B28] Borges L. S. Yechikhov S. Lee Y. I. Rudell J. B. Friese M. B. Burden S. J. (2008). Identification of a motif in the acetylcholine receptor β subunit whose phosphorylation regulates rapsyn association and postsynaptic receptor localization. J. Neurosci. 28, 11468–11476. 10.1523/JNEUROSCI.2508-08.2008 18987183 PMC2606670

[B29] Buchou T. Vernet M. Blond O. Jensen H. H. Pointu H. Olsen B. B. (2003). Disruption of the regulatory beta subunit of protein kinase CK2 in mice leads to a cell-autonomous defect and early embryonic lethality. Mol. Cell Biol. 23, 908–915. 10.1128/MCB.23.3.908-915.2003 12529396 PMC140710

[B30] Burgess D. L. Kohrman D. C. Galt J. Plummer N. W. Jones J. M. Spear B. (1995). Mutation of a new sodium channel gene, Scn8a, in the mouse mutant “motor endplate disease”. Nat. Genet. 10, 461–465. –5. 10.1038/ng0895-461 7670495

[B31] Burgess R. W. Peterson K. A. Johnson M. J. Roix J. J. Welsh I. C. O’Brien T. P. (2004). Evidence for a conserved function in synapse formation reveals Phr1 as a candidate gene for respiratory failure in newborn mice. Mol. Cell Biol. 24, 1096–1105. 10.1128/MCB.24.3.1096-1105.2004 14729956 PMC321423

[B32] Burke S. K. Fenton A. I. Konokhova Y. Hepple R. T. (2021). Variation in muscle and neuromuscular junction morphology between atrophy-resistant and atrophy-prone muscles supports failed re-innervation in aging muscle atrophy. Exp. Gerontol. 156, 111613. 10.1016/j.exger.2021.111613 34740815

[B33] Burré J. Sharma M. Tsetsenis T. Buchman V. Etherton M. R. Südhof T. C. (2010). Alpha-synuclein promotes SNARE-complex assembly *in vivo* and *in vitro* . Science 329, 1663–1667. 10.1126/science.1195227 20798282 PMC3235365

[B34] Caillol G. Vacher H. Musarella M. Bellouze S. Dargent B. Autillo-Touati A. (2012). Motor endplate disease affects neuromuscular junction maturation. Eur. J. Neurosci. 36, 2400–2408. 10.1111/j.1460-9568.2012.08164.x 22642323

[B35] Carpanini S. M. McKie L. Thomson D. Wright A. K. Gordon S. L. Roche S. L. (2014). A novel mouse model of Warburg Micro syndrome reveals roles for RAB18 in eye development and organisation of the neuronal cytoskeleton. Dis. Model Mech. 7, 711–722. 10.1242/dmm.015222 24764192 PMC4036478

[B36] Carrasco D. I. Seburn K. L. Pinter M. J. (2016). Altered terminal Schwann cell morphology precedes denervation in SOD1 mice. Exp. Neurol. 275 (1), 172–181. 10.1016/j.expneurol.2015.09.014 26416261 PMC4768763

[B37] Caruso N. Herberth B. Bartoli M. Puppo F. Dumonceaux J. Zimmermann A. (2013). Deregulation of the protocadherin gene FAT1 alters muscle shapes: Implications for the pathogenesis of facioscapulohumeral dystrophy. PLoS Genet. 9, e1003550. 10.1371/journal.pgen.1003550 23785297 PMC3681729

[B38] Chambers D. M. Peters J. Abbott C. M. (1998). The lethal mutation of the mouse wasted (wst) is a deletion that abolishes expression of a tissue-specific isoform of translation elongation factor 1alpha, encoded by the Eef1a2 gene. Proc. Natl. Acad. Sci. U. S. A. 95, 4463–4468. 10.1073/pnas.95.8.4463 9539760 PMC22512

[B39] Chandra S. Gallardo G. Fernández-Chacón R. Schlüter O. M. Südhof T. C. (2005). Alpha-synuclein cooperates with CSPalpha in preventing neurodegeneration. Cell 123, 383–396. 10.1016/j.cell.2005.09.028 16269331

[B40] Chen F. Qian L. Yang Z.-H. Huang Y. Ngo S. T. Ruan N.-J. (2007). Rapsyn interaction with calpain stabilizes AChR clusters at the neuromuscular junction. Neuron 55, 247–260. 10.1016/j.neuron.2007.06.031 17640526

[B41] Chen P.-C. Bhattacharyya B. J. Hanna J. Minkel H. Wilson J. A. Finley D. (2011). Ubiquitin homeostasis is critical for synaptic development and function. J. Neurosci. 31, 17505–17513. 10.1523/JNEUROSCI.2922-11.2011 22131412 PMC3253363

[B42] Chen P.-C. Qin L.-N. Li X.-M. Walters B. J. Wilson J. A. Mei L. (2009). The proteasome-associated deubiquitinating enzyme Usp14 is essential for the maintenance of synaptic ubiquitin levels and the development of neuromuscular junctions. J. Neurosci. 29, 10909–10919. 10.1523/JNEUROSCI.2635-09.2009 19726649 PMC2766780

[B43] Cheusova T. Khan M. A. Schubert S. W. Gavin A.-C. Buchou T. Jacob G. (2006). Casein kinase 2-dependent serine phosphorylation of MuSK regulates acetylcholine receptor aggregation at the neuromuscular junction. Genes Dev. 20, 1800–1816. 10.1101/gad.375206 16818610 PMC1522076

[B44] Choi H. Y. Liu Y. Tennert C. Sugiura Y. Karakatsani A. Kröger S. (2013). APP interacts with LRP4 and agrin to coordinate the development of the neuromuscular junction in mice. Elife 2, e00220. 10.7554/eLife.00220 23986861 PMC3748711

[B45] Clarac F. Pearlstein E. (2007). Invertebrate preparations and their contribution to neurobiology in the second half of the 20th century. Brain Res. Rev. 54, 113–161. 10.1016/j.brainresrev.2006.12.007 17500093

[B46] Clerc P. Lipnick S. Willett C. (2016). A look into the future of ALS research. Drug Discov. Today 21, 939–949. 10.1016/j.drudis.2016.02.002 26861067

[B47] Cohen E. Bonne G. Rivier F. Hamroun D. (2021). The 2022 version of the gene table of neuromuscular disorders (nuclear genome). Neuromuscul. Disord. 31, 1313–1357. 10.1016/j.nmd.2021.11.004 34930546

[B48] Couesnon A. Offner N. Bernard V. Chaverot N. Backer S. Dimitrov A. (2013). CLIPR-59: A protein essential for neuromuscular junction stability during mouse late embryonic development. Development 140, 1583–1593. 10.1242/dev.087106 23482493

[B49] Couteaux R. (1947). Contribution a l’etude de la synapse myoneurale. Rev. Canad Biol. 6, 563–711.

[B50] Couteaux R. (1955). Localization of cholinesterases at neuromuscular junctions. Int. Rev. Cytol. 4, 335–375. 10.1016/S0074-7696(08)60463-5

[B51] Dale H. H. (1914). The action of certain esters and ethers of choline and their relation to muscarine. J. Pharmacol. Exp. Ther. 6, 147–190.

[B52] D’Amato C. J. Hicks S. P. (1965). Neuropathologic alterations in the ataxia (paralytic) mouse. Arch. Pathol. (Chic) 80, 604–612. 5855800

[B53] Davegårdh C. Hall Wedin E. Broholm C. Henriksen T. I. Pedersen M. Pedersen B. K. (2019). Sex influences DNA methylation and gene expression in human skeletal muscle myoblasts and myotubes. Stem Cell Res. Ther. 10, 26. 10.1186/s13287-018-1118-4 30646953 PMC6332625

[B54] de Pace R. Britt D. J. Mercurio J. Foster A. M. Djavaherian L. Hoffmann V. (2020). Synaptic vesicle precursors and lysosomes are transported by different mechanisms in the axon of mammalian neurons. Cell Rep. 31, 107775. 10.1016/j.celrep.2020.107775 32553155 PMC7478246

[B55] de Paola E. Forcina L. Pelosi L. Pisu S. la Rosa P. Cesari E. (2020). Sam68 splicing regulation contributes to motor unit establishment in the postnatal skeletal muscle. Life Sci. Alliance 3, e201900637. 10.26508/lsa.201900637 32753528 PMC7409371

[B56] Deconinck A. E. Potter A. C. Tinsley J. M. Wood S. J. Vater R. Young C. (1997a). Postsynaptic abnormalities at the neuromuscular junctions of utrophin-deficient mice. J. Cell Biol. 136, 883–894. 10.1083/jcb.136.4.883 9049253 PMC2132499

[B57] Deconinck A. E. Rafael J. A. Skinner J. A. Brown S. C. Potter A. C. Metzinger L. (1997b). Utrophin-dystrophin-deficient mice as a model for Duchenne muscular dystrophy. Cell 90, 717–727. 10.1016/s0092-8674(00)80532-2 9288751

[B58] Deschenes M. R. Hurst T. E. Ramser A. E. Sherman E. G. (2013). Presynaptic to postsynaptic relationships of the neuromuscular junction are held constant across age and muscle fiber type. Dev. Neurobiol. 73, 744–753. 10.1002/dneu.22095 23696094

[B59] Dobrowolny G. Barbiera A. Sica G. Scicchitano B. M. (2021). Age-related alterations at neuromuscular junction: Role of oxidative stress and epigenetic modifications. Cells 10, 1307. 10.3390/cells10061307 34074012 PMC8225025

[B60] Doig J. Griffiths L. A. Peberdy D. Dharmasaroja P. Vera M. Davies F. J. C. (2013). *In vivo* characterization of the role of tissue-specific translation elongation factor 1A2 in protein synthesis reveals insights into muscle atrophy. FEBS J. 280, 6528–6540. 10.1111/febs.12554 24460877 PMC4163635

[B61] Dubowitz V. (1991). Neuromuscular disorders: Gene location. Neuromuscul. Disord. 1, 75–76. 10.1016/0960-8966(91)90050-3 1822779

[B62] Duchen L. W. (1970). Hereditary motor end-plate disease in the mouse: Light and electron microscopic studies. J. Neurol. Neurosurg. Psychiatry 33, 238–250. 10.1136/jnnp.33.2.238 4315332 PMC493449

[B63] Duchen L. W. Searle A. G. Strich A. J. (1967). An hereditary motor endplate disease in the mouse. J. Physiol. (Lond.) 189, 4–6P.

[B64] Duchen L. W. Stefani E. (1971). Electrophysiological studies of neuromuscular transmission in hereditary “motor end-plate disease” of the mouse. J. Physiol. 212, 535–548. 10.1113/jphysiol.1971.sp009340 4323310 PMC1395673

[B65] Engel A. G. (2020). “Congenital myasthenic syndromes,” in Rosenberg’s molecular and genetic basis of neurological and psychiatric disease. Editors RA P Al. E (Amsterdam, Netherlands: Elsevier), 539–558. 10.1016/B978-0-12-813866-3.00032-1

[B66] Engel A. G. Shen X.-M. Selcen D. Sine S. M. (2015). Congenital myasthenic syndromes: Pathogenesis, diagnosis, and treatment. Lancet Neurol. 14, 420–434. 10.1016/S1474-4422(14)70201-7 25792100 PMC4520251

[B67] Engel A. G. (2008). The neuromuscular junction. Handb. Clin. Neurology 91, 103–148. Elsevier. 10.1016/S0072-9752(07)01503-5 18631841

[B68] Escher P. Lacazette E. Courtet M. Blindenbacher A. Landmann L. Bezakova G. (2005). Synapses form in skeletal muscles lacking neuregulin receptors. Science 308, 1920–1923. 10.1126/science.1108258 15976301

[B69] Fatt P. Katz B. (1951). An analysis of the end-plate potential recorded with an intracellular electrode. J. Physiol. 115, 320–370. 10.1113/JPHYSIOL.1951.SP004675 14898516 PMC1392060

[B70] Fatt P. Katz B. (1952). Spontaneous subthreshold activity at motor nerve endings. J. Physiol. 117, 109–128. 10.1113/jphysiol.1952.sp004735 14946732 PMC1392564

[B71] Feng G. Tintrup H. Kirsch J. Nichol M. C. Kuhse J. Betz H. (1998). Dual requirement for gephyrin in glycine receptor clustering and molybdoenzyme activity. Science 282, 1321–1324. 10.1126/science.282.5392.1321 9812897

[B72] Feng X. Zhang T. Ralston E. Ludlow C. L. (2012). Differences in neuromuscular junctions of laryngeal and limb muscles in rats. Laryngoscope 122, 1093–1098. 10.1002/lary.23218 22374515 PMC3462430

[B73] Fernández-Chacón R. Wölfel M. Nishimune H. Tabares L. Schmitz F. Castellano-Muñoz M. (2004). The synaptic vesicle protein CSP alpha prevents presynaptic degeneration. Neuron 42, 237–251. 10.1016/s0896-6273(04)00190-4 15091340

[B74] Ferns M. Campanelli J. T. Hoch W. Scheller R. H. Hall Z. M. (1993). The ability of agrin to cluster AChRs depends on alternative splicing and on cell surface proteoglycans. Neuron 11, 491–502. 10.1016/0896-6273(93)90153-i 8398142

[B75] Fil D. DeLoach A. Yadav S. Alkam D. MacNicol M. Singh A. (2017). Mutant Profilin1 transgenic mice recapitulate cardinal features of motor neuron disease. Hum. Mol. Genet. 26, 686–701. 10.1093/hmg/ddw429 28040732 PMC5968635

[B76] Flucher B. E. Daniels M. P. (1989). Distribution of Na+ channels and ankyrin in neuromuscular junctions is complementary to that of acetylcholine receptors and the 43 kd protein. Neuron 3, 163–175. 10.1016/0896-6273(89)90029-9 2560390

[B77] Frey D. Laux T. Xu L. Schneider C. Caroni P. (2000). Shared and unique roles of CAP23 and GAP43 in actin regulation, neurite outgrowth, and anatomical plasticity. J. Cell Biol. 149, 1443–1454. 10.1083/jcb.149.7.1443 10871284 PMC2175140

[B78] Gaudet A. D. Leung M. Poirier F. Kadoya T. Horie H. Ramer M. S. (2009). A role for galectin-1 in the immune response to peripheral nerve injury. Exp. Neurol. 220, 320–327. 10.1016/j.expneurol.2009.09.007 19766118

[B79] Geerts C. J. Plomp J. J. Koopmans B. Loos M. van der Pijl E. M. van der Valk M. A. (2015). Tomosyn-2 is required for normal motor performance in mice and sustains neurotransmission at motor endplates. Brain Struct. Funct. 220, 1971–1982. 10.1007/s00429-014-0766-0 24744148

[B80] Glass D. J. Bowen D. C. Stitt T. N. Radziejewski C. Bruno J. Ryan T. E. (1996). Agrin acts via a MuSK receptor complex. Cell 85, 513–523. 10.1016/s0092-8674(00)81252-0 8653787

[B81] Gomeza J. Ohno K. Hülsmann S. Armsen W. Eulenburg V. Richter D. W. (2003). Deletion of the mouse glycine transporter 2 results in a hyperekplexia phenotype and postnatal lethality. Neuron 40, 797–806. 10.1016/s0896-6273(03)00673-1 14622583

[B82] Goodwin L. O. Splinter E. Davis T. L. Urban R. He H. Braun R. E. (2019). Large-scale discovery of mouse transgenic integration sites reveals frequent structural variation and insertional mutagenesis. Genome Res. 29, 494–505. 10.1101/gr.233866.117 30659012 PMC6396414

[B83] Grady R. M. Grange R. W. Lau K. S. Maimone M. M. Nichol M. C. Stull J. T. (1999). Role for alpha-dystrobrevin in the pathogenesis of dystrophin-dependent muscular dystrophies. Nat. Cell Biol. 1, 215–220. 10.1038/12034 10559919

[B84] Grady R. M. Merlie J. P. Sanes J. R. (1997a). Subtle neuromuscular defects in utrophin-deficient mice. J. Cell Biol. 136, 871–882. 10.1083/jcb.136.4.871 9049252 PMC2132496

[B85] Grady R. M. Teng H. Nichol M. C. Cunningham J. C. Wilkinson R. S. Sanes J. R. (1997b). Skeletal and cardiac myopathies in mice lacking utrophin and dystrophin: A model for duchenne muscular dystrophy. Cell 90, 729–738. 10.1016/s0092-8674(00)80533-4 9288752

[B86] Grady R. M. Zhou H. Cunningham J. M. Henry M. D. Campbell K. P. Sanes J. R. (2000). Maturation and maintenance of the neuromuscular synapse: Genetic evidence for roles of the dystrophin-glycoprotein complex. Neuron 25, 279–293. 10.1016/s0896-6273(00)80894-6 10719885

[B87] Greschik H. Duteil D. Messaddeq N. Willmann D. Arrigoni L. Sum M. (2017). The histone code reader Spin1 controls skeletal muscle development. Cell Death Dis. 8, e3173, e3173. 10.1038/cddis.2017.468 29168801 PMC5775400

[B88] Griffiths L. A. Doig J. Churchhouse A. M. D. Davies F. C. J. Squires C. E. Newbery H. J. (2012). Haploinsufficiency for translation elongation factor eEF1A2 in aged mouse muscle and neurons is compatible with normal function. PLoS One 7, e41917. 10.1371/journal.pone.0041917 22848658 PMC3405021

[B89] Guasch R. M. Scambler P. Jones G. E. Ridley A. J. (1998). RhoE regulates actin cytoskeleton organization and cell migration. Mol. Cell Biol. 18, 4761–4771. 10.1128/MCB.18.8.4761 9671486 PMC109062

[B90] Hall E. A. Nahorski M. S. Murray L. M. Shaheen R. Perkins E. Dissanayake K. N. (2017). PLAA mutations cause a lethal infantile epileptic encephalopathy by disrupting ubiquitin-mediated endolysosomal degradation of synaptic proteins. Am. J. Hum. Genet. 100, 706–724. 10.1016/j.ajhg.2017.03.008 28413018 PMC5420347

[B91] Hanada T. Weitzer S. Mair B. Bernreuther C. Wainger B. J. Ichida J. (2013). CLP1 links tRNA metabolism to progressive motor-neuron loss. Nature 495, 474–480. 10.1038/nature11923 23474986 PMC3674495

[B92] Hanson M. G. Fregoso V. L. Vrana J. D. Tucker C. L. Niswander L. A. (2014). Peripheral nervous system defects in a mouse model for peroxisomal biogenesis disorders. Dev. Biol. 395, 84–95. 10.1016/j.ydbio.2014.08.026 25176044 PMC4190158

[B93] Helmbacher F. (2018). Tissue-specific activities of the Fat1 cadherin cooperate to control neuromuscular morphogenesis. PLoS Biol. 16, e2004734. 10.1371/journal.pbio.2004734 29768404 PMC5973635

[B94] Hideyama T. Yamashita T. Suzuki T. Tsuji S. Higuchi M. Seeburg P. H. (2010). Induced loss of ADAR2 engenders slow death of motor neurons from Q/R site-unedited GluR2. J. Neurosci. 30, 11917–11925. 10.1523/JNEUROSCI.2021-10.2010 20826656 PMC6633551

[B95] Horstick E. J. Linsley J. W. Dowling J. J. Hauser M. A. McDonald K. K. Ashley-Koch A. (2013). Stac3 is a component of the excitation-contraction coupling machinery and mutated in Native American myopathy. Nat. Commun. 4, 1952. 10.1038/ncomms2952 23736855 PMC4056023

[B96] Hosseinibarkooie S. Peters M. Torres-Benito L. Rastetter R. H. Hupperich K. Hoffmann A. (2016). The power of human protective modifiers: PLS3 and CORO1C unravel impaired endocytosis in spinal muscular atrophy and rescue SMA phenotype. Am. J. Hum. Genet. 99, 647–665. 10.1016/j.ajhg.2016.07.014 27499521 PMC5011078

[B97] Huang B.-T. Chang P.-Y. Su C.-H. Chao C. C.-K. Lin-Chao S. (2012). Gas7-deficient mouse reveals roles in motor function and muscle fiber composition during aging. PLoS One 7, e37702. 10.1371/journal.pone.0037702 22662195 PMC3360064

[B98] Ibraghimov-Beskrovnaya O. Ervasti J. M. Leveille C. J. Slaughter C. A. Sernett S. W. Campbell K. P. (1992). Primary structure of dystrophin-associated glycoproteins linking dystrophin to the extracellular matrix. Nature 355, 696–702. 10.1038/355696a0 1741056

[B99] Jaworski A. Smith C. L. Burden S. J. (2007). GA-binding protein is dispensable for neuromuscular synapse formation and synapse-specific gene expression. Mol. Cell Biol. 27, 5040–5046. 10.1128/MCB.02228-06 17485447 PMC1951497

[B100] Jones R. A. Harrison C. Eaton S. L. Llavero Hurtado M. Graham L. C. Alkhammash L. (2017). Cellular and molecular anatomy of the human neuromuscular junction. Cell Rep. 21, 2348–2356. 10.1016/j.celrep.2017.11.008 29186674 PMC5723673

[B101] Jones R. A. Reich C. D. Dissanayake K. N. Kristmundsdottir F. Findlater G. S. Ribchester R. R. (2016). NMJ-morph reveals principal components of synaptic morphology influencing structure-function relationships at the neuromuscular junction. Open Biol. 6, 160240. 10.1098/rsob.160240 27927794 PMC5204123

[B102] Kaeser P. S. Deng L. Wang Y. Dulubova I. Liu X. Rizo J. (2011). RIM proteins tether Ca2+ channels to presynaptic active zones via a direct PDZ-domain interaction. Cell 144, 282–295. 10.1016/j.cell.2010.12.029 21241895 PMC3063406

[B103] Kaether C. Scheuermann J. Fassler M. Zilow S. Shirotani K. Valkova C. (2007). Endoplasmic reticulum retention of the gamma-secretase complex component Pen2 by Rer1. EMBO Rep. 8, 743–748. 10.1038/sj.embor.7401027 17668005 PMC1978084

[B104] Kaja S. Todorov B. van de Ven R. C. G. Ferrari M. D. Frants R. R. van den Maagdenberg A. M. J. M. (2007). Redundancy of Cav2.1 channel accessory subunits in transmitter release at the mouse neuromuscular junction. Brain Res. 1143, 92–101. 10.1016/j.brainres.2007.01.063 17320843

[B105] Kang H. Tian L. Mikesh M. Lichtman J. W. Thompson W. J. (2014). Terminal Schwann cells participate in neuromuscular synapse remodeling during reinnervation following nerve injury. J. Neurosci. 34, 6323–6333. 10.1523/JNEUROSCI.4673-13.2014 24790203 PMC4004816

[B106] Khalyfa A. Bourbeau D. Chen E. Petroulakis E. Pan J. Xu S. (2001). Characterization of elongation factor-1A (eEF1A-1) and eEF1A-2/S1 protein expression in normal and wasted mice. J. Biol. Chem. 276, 22915–22922. 10.1074/jbc.M101011200 11294870

[B107] Kim M. J. Whitehead N. P. Bible K. L. Adams M. E. Froehner S. C. (2019). Mice lacking α-β1- and β2-syntrophins exhibit diminished function and reduced dystrophin expression in both cardiac and skeletal muscle. Hum. Mol. Genet. 28, 386–395. 10.1093/hmg/ddy341 30256963 PMC6337692

[B108] Kim N. Stiegler A. L. Cameron T. O. Hallock P. T. Gomez A. M. Huang J. H. (2008). Lrp4 is a receptor for Agrin and forms a complex with MuSK. Cell 135, 334–342. 10.1016/j.cell.2008.10.002 18848351 PMC2933840

[B109] Kirjavainen A. Singh P. Lahti L. Seja P. Lelkes Z. Makkonen A. (2022). Gata2, Nkx2-2 and Skor2 form a transcription factor network regulating development of a midbrain GABAergic neuron subtype with characteristics of REM-sleep regulatory neurons. Development 149, dev200937. 10.1242/dev.200937 35815619

[B110] Kiyonaka S. Wakamori M. Miki T. Uriu Y. Nonaka M. Bito H. (2007). RIM1 confers sustained activity and neurotransmitter vesicle anchoring to presynaptic Ca2+ channels. Nat. Neurosci. 10, 691–701. 10.1038/nn1904 17496890 PMC2687938

[B111] Klockner I. Schutt C. Gerhardt T. Boettger T. Braun T. (2022). Control of CRK-RAC1 activity by the miR-1/206/133 miRNA family is essential for neuromuscular junction function. Nat. Commun. 13, 3180. 10.1038/s41467-022-30778-7 35676269 PMC9178026

[B112] Kuhne W. (1863). Die muskelspindeln. Arch. fur Pathol. Anat. Physiol. fur Klin. Med. 28, 528e538. 10.1007/BF01942820

[B113] Lanuza M. A. Besalduch N. González C. Santafé M. M. Garcia N. Tomàs M. (2010). Decreased phosphorylation of δ and ε subunits of the acetylcholine receptor coincides with delayed postsynaptic maturation in PKC θ deficient mouse. Exp. Neurol. 225, 183–195. 10.1016/j.expneurol.2010.06.014 20599977

[B114] Lee K.-Y. Li M. Manchanda M. Batra R. Charizanis K. Mohan A. (2013). Compound loss of muscleblind-like function in myotonic dystrophy. EMBO Mol. Med. 5, 1887–1900. 10.1002/emmm.201303275 24293317 PMC3914532

[B115] Lenell C. Johnson A. M. (2017). Sexual dimorphism in laryngeal muscle fibers and ultrasonic vocalizations in the adult rat. Laryngoscope 127, E270–E276. 10.1002/lary.26561 28304076 PMC5819991

[B116] Letts V. A. Felix R. Biddlecome G. H. Arikkath J. Mahaffey C. L. Valenzuela A. (1998). The mouse stargazer gene encodes a neuronal Ca2+-channel gamma subunit. Nat. Genet. 19, 340–347. 10.1038/1228 9697694

[B117] Leu M. Bellmunt E. Schwander M. Fariñas I. Brenner H. R. Müller U. (2003). Erbb2 regulates neuromuscular synapse formation and is essential for muscle spindle development. Development 130, 2291–2301. 10.1242/dev.00447 12702645

[B118] Lewcock J. W. Genoud N. Lettieri K. Pfaff S. L. (2007). The ubiquitin ligase Phr1 regulates axon outgrowth through modulation of microtubule dynamics. Neuron 56, 604–620. 10.1016/j.neuron.2007.09.009 18031680

[B119] Li H. Wang Z. Wang B. Guo Q. Dolios G. Tabuchi K. (2010). Genetic dissection of the amyloid precursor protein in developmental function and amyloid pathogenesis. J. Biol. Chem. 285, 30598–30605. 10.1074/jbc.M110.137729 20693289 PMC2945554

[B120] Li M.-X. Jia M. Yang L.-X. Jiang H. Lanuza M. A. Gonzalez C. M. (2004). The role of the theta isoform of protein kinase C (PKC) in activity-dependent synapse elimination: Evidence from the PKC theta knock-out mouse *in vivo* and *in vitro* . J. Neurosci. 24, 3762–3769. 10.1523/JNEUROSCI.3930-03.2004 15084656 PMC6729339

[B121] Lin S. Landmann L. Ruegg M. A. Brenner H. R. (2008). The role of nerve-versus muscle-derived factors in mammalian neuromuscular junction formation. J. Neurosci. 28, 3333–3340. 10.1523/JNEUROSCI.5590-07.2008 18367600 PMC6670584

[B122] Lin W. Burgess R. W. Dominguez B. Pfaff S. L. Sanes J. R. Lee K. F. (2001). Distinct roles of nerve and muscle in postsynaptic differentiation of the neuromuscular synapse. Nature 410, 1057–1064. 10.1038/35074025 11323662

[B123] Lin W. Dominguez B. Yang J. Aryal P. Brandon E. P. Gage F. H. (2005). Neurotransmitter acetylcholine negatively regulates neuromuscular synapse formation by a Cdk5-dependent mechanism. Neuron 46, 569–579. 10.1016/j.neuron.2005.04.002 15944126

[B124] Lin W. Sanchez H. B. Deerinck T. Morris J. K. Ellisman M. Lee K. F. (2000). Aberrant development of motor axons and neuromuscular synapses in erbB2-deficient mice. Proc. Natl. Acad. Sci. U. S. A. 97, 1299–1304. 10.1073/pnas.97.3.1299 10655525 PMC15603

[B125] Lindqvist J. Torvaldson E. Gullmets J. Karvonen H. Nagy A. Taimen P. (2017). Nestin contributes to skeletal muscle homeostasis and regeneration. J. Cell Sci. 130, 2833–2842. 10.1242/jcs.202226 28733456

[B126] Liu K. Jones S. Minis A. Rodriguez J. Molina H. Steller H. (2019). PI31 is an adaptor protein for proteasome transport in axons and required for synaptic development. Dev. Cell 50, 509–524.e10. 10.1016/j.devcel.2019.06.009 31327739 PMC6702053

[B127] Liu N. Williams A. H. Maxeiner J. M. Bezprozvannaya S. Shelton J. M. Richardson J. A. (2012). microRNA-206 promotes skeletal muscle regeneration and delays progression of Duchenne muscular dystrophy in mice. J. Clin. Invest. 122, 2054–2065. 10.1172/JCI62656 22546853 PMC3366415

[B128] Liu Y Y. Sugiura Y. Chen F. Lee K. F. Ye Q. Lin W. (2019). Blocking skeletal muscle DHPRs/Ryr1 prevents neuromuscular synapse loss in mutant mice deficient in type III Neuregulin 1 (CRD-Nrg1). PLoS Genet. 15, e1007857. 10.1371/journal.pgen.1007857 30870432 PMC6417856

[B129] Liu Y. Padgett D. Takahashi M. Li H. Sayeed A. Teichert R. W. (2008). Essential roles of the acetylcholine receptor ɣ-subunit in neuromuscular synaptic patterning. Development 135, 1957–1967. 10.1242/dev.018119 18434415 PMC2650015

[B130] Lnenicka G. A. (2020). Crayfish and Drosophila NMJs. Neurosci. Lett. 732, 135110. 10.1016/j.neulet.2020.135110 32497734

[B131] Lüningschrör P. Werner G. Stroobants S. Kakuta S. Dombert B. Sinske D. (2020). The FTLD risk factor TMEM106B regulates the transport of lysosomes at the axon initial segment of motoneurons. Cell Rep. 30, 3506–3519.e6. 10.1016/j.celrep.2020.02.060 32160553

[B132] Mabbitt P. D. Loreto A. Déry M.-A. Fletcher A. J. Stanley M. Pao K.-C. (2020). Structural basis for RING-Cys-Relay E3 ligase activity and its role in axon integrity. Nat. Chem. Biol. 16, 1227–1236. 10.1038/s41589-020-0598-6 32747811 PMC7610530

[B133] Mandela P. Yankova M. Conti L. H. Ma X.-M. Grady J. Eipper B. A. (2012). Kalrn plays key roles within and outside of the nervous system. BMC Neurosci. 13, 136. 10.1186/1471-2202-13-136 23116210 PMC3541206

[B134] Marshall A. G. Watson J. A. Hallengren J. J. Walters B. J. Dobrunz L. E. Francillon L. (2013). Genetic background alters the severity and onset of neuromuscular disease caused by the loss of ubiquitin-specific protease 14 (usp14). PLoS One 8, e84042. 10.1371/journal.pone.0084042 24358326 PMC3865287

[B135] Martin P. B. Kigoshi-Tansho Y. Sher R. B. Ravenscroft G. Stauffer J. E. Kumar R. (2020). NEMF mutations that impair ribosome-associated quality control are associated with neuromuscular disease. Nat. Commun. 11, 4625. 10.1038/s41467-020-18327-6 32934225 PMC7494853

[B136] McGovern V. L. Massoni-Laporte A. Wang X. Le T. T. Le H. T. Beattie C. E. (2015). Plastin 3 expression does not modify spinal muscular atrophy severity in the ∆7 SMA mouse. PLoS One 10, e0132364. 10.1371/journal.pone.0132364 26134627 PMC4489873

[B137] McMahan U. J. (1990). The agrin hypothesis. Cold Spring Harb. Symp. Quant. Biol. 55, 407–418. 10.1101/sqb.1990.055.01.041 1966767

[B138] Medrihan L. Rohlmann A. Fairless R. Andrae J. Döring M. Missler M. (2009). Neurobeachin, a protein implicated in membrane protein traffic and autism, is required for the formation and functioning of central synapses. J. Physiol. 587, 5095–5106. 10.1113/jphysiol.2009.178236 19723784 PMC2790251

[B139] Mejia Maza A. Jarvis S. Lee W. C. Cunningham T. J. Schiavo G. Secrier M. (2021). NMJ-Analyser identifies subtle early changes in mouse models of neuromuscular disease. Sci. Rep. 11, 12251. 10.1038/s41598-021-91094-6 34112844 PMC8192785

[B140] Messéant J. Ezan J. Delers P. Glebov K. Marchiol C. Lager F. (2017). Wnt proteins contribute to neuromuscular junction formation through distinct signaling pathways. Development 144, 1712–1724. 10.1242/dev.146167 28348167

[B141] Minis A. Rodriguez J. A. Levin A. Liu K. Govek E.-E. Hatten M. E. (2019). The proteasome regulator PI31 is required for protein homeostasis, synapse maintenance, and neuronal survival in mice. Proc. Natl. Acad. Sci. U. S. A. 116, 24639–24650. 10.1073/pnas.1911921116 31754024 PMC6900516

[B142] Mocholí E. Ballester-Lurbe B. Arqué G. Poch E. Peris B. Guerri C. (2011). RhoE deficiency produces postnatal lethality, profound motor deficits and neurodevelopmental delay in mice. PLoS One 6, e19236. 10.1371/journal.pone.0019236 21552537 PMC3084285

[B143] Mohseni P. Sung H.-K. Murphy A. J. Laliberte C. L. Pallari H.-M. Henkelman M. (2011). Nestin is not essential for development of the CNS but required for dispersion of acetylcholine receptor clusters at the area of neuromuscular junctions. J. Neurosci. 31, 11547–11552. 10.1523/JNEUROSCI.4396-10.2011 21832185 PMC6623112

[B144] Morelli K. H. Seburn K. L. Schroeder D. G. Spaulding E. L. Dionne L. A. Cox G. A. (2017). Severity of demyelinating and axonal neuropathy mouse models is modified by genes affecting structure and function of peripheral nodes. Cell Rep. 18, 3178–3191. 10.1016/j.celrep.2017.03.009 28355569 PMC5415377

[B145] Musarella M. Alcaraz G. Caillol G. Boudier J.-L. Couraud F. Autillo-Touati A. (2006). Expression of Nav1.6 sodium channels by Schwann cells at neuromuscular junctions: Role in the motor endplate disease phenotype. Glia 53, 13–23. 10.1002/glia.20252 16078241

[B146] Nakatani T. Minaki Y. Kumai M. Nitta C. Ono Y. (2014). The c-Ski family member and transcriptional regulator Corl2/Skor2 promotes early differentiation of cerebellar Purkinje cells. Dev. Biol. 388, 68–80. 10.1016/j.ydbio.2014.01.016 24491816

[B147] Nakka K. Ghigna C. Gabellini D. Dilworth F. J. (2018). Diversification of the muscle proteome through alternative splicing. Skelet. Muscle 8, 8. 10.1186/s13395-018-0152-3 29510724 PMC5840707

[B148] Nelson B. R. Wu F. Liu Y. Anderson D. M. McAnally J. Lin W. (2013). Skeletal muscle-specific T-tubule protein STAC3 mediates voltage-induced Ca2+ release and contractility. Proc. Natl. Acad. Sci. U. S. A. 110, 11881–11886. 10.1073/pnas.1310571110 23818578 PMC3718085

[B149] Newbern J. Birchmeier C. (2010). Nrg1/ErbB signaling networks in Schwann cell development and myelination. Semin. Cell Dev. Biol. 21, 922–928. 10.1016/j.semcdb.2010.08.008 20832498 PMC2991617

[B150] Newbery H. J. Gillingwater T. H. Dharmasaroja P. Peters J. Wharton S. B. Thomson D. (2005). Progressive loss of motor neuron function in wasted mice: Effects of a spontaneous null mutation in the gene for the eEF1 A2 translation factor. J. Neuropathol. Exp. Neurol. 64, 295–303. 10.1093/jnen/64.4.295 15835265

[B151] Nguyen S. Meletis K. Fu D. Jhaveri S. Jaenisch R. (2007). Ablation of de novo DNA methyltransferase Dnmt3a in the nervous system leads to neuromuscular defects and shortened lifespan. Dev. Dyn. 236, 1663–1676. 10.1002/dvdy.21176 17477386

[B152] Nian F.-S. Li L.-L. Cheng C.-Y. Wu P.-C. Lin Y.-T. Tang C.-Y. (2019). Rab18 collaborates with Rab7 to modulate lysosomal and autophagy activities in the nervous system: An overlapping mechanism for warburg micro syndrome and charcot-marie-tooth neuropathy type 2B. Mol. Neurobiol. 56, 6095–6105. 10.1007/s12035-019-1471-z 30721447

[B153] Noebels J. L. Qiao X. Bronson R. T. Spencer C. Davisson M. T. (1990). Stargazer: A new neurological mutant on chromosome 15 in the mouse with prolonged cortical seizures. Epilepsy Res. 7, 129–135. 10.1016/0920-1211(90)90098-g 2289471

[B154] Nuytens K. Tuand K. Fu Q. Stijnen P. Pruniau V. Meulemans S. (2014). The dwarf phenotype in GH240B mice, haploinsufficient for the autism candidate gene Neurobeachin, is caused by ectopic expression of recombinant human growth hormone. PLoS One 9, e109598. 10.1371/journal.pone.0109598 25333629 PMC4198124

[B155] Okada K. Inoue A. Okada M. Murata Y. Kakuta S. Jigami T. (2006). The muscle protein Dok-7 is essential for neuromuscular synaptogenesis. Science 312, 1802–1805. 10.1126/science.1127142 16794080

[B156] O’Leary D. A. Noakes P. G. Lavidis N. A. Kola I. Hertzog P. J. Ristevski S. (2007). Targeting of the ETS factor GABPalpha disrupts neuromuscular junction synaptic function. Mol. Cell Biol. 27, 3470–3480. 10.1128/MCB.00659-06 17325042 PMC1899955

[B157] Olson E. N. Arnold H. H. Rigby P. W. Wold B. J. (1996). Know your neighbors: Three phenotypes in null mutants of the myogenic bHLH gene MRF4. Cell 85, 1–4. 10.1016/s0092-8674(00)81073-9 8620528

[B158] Oprea G. E. Kröber S. McWhorter M. L. Rossoll W. Müller S. Krawczak M. (2008). Plastin 3 is a protective modifier of autosomal recessive spinal muscular atrophy. Science 320, 524–527. 10.1126/science.1155085 18440926 PMC4908855

[B159] O’Reilly J. Ono-Moore K. D. Chintapalli S. v. Rutkowsky J. M. Tolentino T. Lloyd K. C. K. (2021). Sex differences in skeletal muscle revealed through fiber type, capillarity, and transcriptomics profiling in mice. Physiol. Rep. 9, e15031. 10.14814/phy2.15031 34545692 PMC8453262

[B160] Penzes P. Johnson R. C. Alam M. R. Kambampati V. Mains R. E. Eipper B. A. (2000). An isoform of kalirin, a brain-specific GDP/GTP exchange factor, is enriched in the postsynaptic density fraction. J. Biol. Chem. 275, 6395–6403. 10.1074/jbc.275.9.6395 10692441

[B161] Peris B. Gonzalez-Granero S. Ballester-Lurbe B. García-Verdugo J.-M. Pérez-Roger I. Guerri C. (2012). Neuronal polarization is impaired in mice lacking RhoE expression. J. Neurochem. 121, 903–914. 10.1111/j.1471-4159.2012.07733.x 22428561

[B162] Pfister J. Zenker W. (1984). The splenius capitis muscle of the rat, architecture and histochemistry, afferent and efferent innervation as compared with that of the quadriceps muscle. Anat. Embryol. Berl. 169, 79–89. 10.1007/BF00300589 6721223

[B163] Pietri T. Eder O. Breau M. A. Topilko P. Blanche M. Brakebusch C. (2004). Conditional beta1-integrin gene deletion in neural crest cells causes severe developmental alterations of the peripheral nervous system. Development 131, 3871–3883. 10.1242/dev.01264 15253938

[B164] Plachta N. Annaheim C. Bissière S. Lin S. Rüegg M. Hoving S. (2007). Identification of a lectin causing the degeneration of neuronal processes using engineered embryonic stem cells. Nat. Neurosci. 10, 712–719. 10.1038/nn1897 17486104

[B165] Plomp J. J. Huijbers M. G. M. Verschuuren J. J. G. M. (2017). Neuromuscular synapse electrophysiology in myasthenia gravis animal models. Ann. N. Y. Acad. Sci. 1412, 146–153. 10.1111/nyas.13507 29068559

[B166] Polster A. Nelson B. R. Olson E. N. Beam K. G. (2016). Stac3 has a direct role in skeletal muscle-type excitation-contraction coupling that is disrupted by a myopathy-causing mutation. Proc. Natl. Acad. Sci. U. S. A. 113, 10986–10991. 10.1073/pnas.1612441113 27621462 PMC5047181

[B167] Quintá H. R. Pasquini J. M. Rabinovich G. A. Pasquini L. A. (2014). Glycan-dependent binding of galectin-1 to neuropilin-1 promotes axonal regeneration after spinal cord injury. Cell Death Differ. 21, 941–955. 10.1038/cdd.2014.14 24561343 PMC4013512

[B168] Reichenstein I. Eitan C. Diaz-Garcia S. Haim G. Magen I. Siany A. (2019). Human genetics and neuropathology suggest a link between miR-218 and amyotrophic lateral sclerosis pathophysiology. Sci. Transl. Med. 11, eaav5264. 10.1126/scitranslmed.aav5264 31852800 PMC7057809

[B169] Reinholt B. M. Ge X. Cong X. Gerrard D. E. Jiang H. (2013). Stac3 is a novel regulator of skeletal muscle development in mice. PLoS One 8, e62760. 10.1371/journal.pone.0062760 23626854 PMC3633831

[B170] Richard S. Torabi N. Franco G. V. Tremblay G. A. Chen T. Vogel G. (2005). Ablation of the Sam68 RNA binding protein protects mice from age-related bone loss. PLoS Genet. 1, e74. 10.1371/journal.pgen.0010074 16362077 PMC1315279

[B171] Rieker C. Dev K. K. Lehnhoff K. Barbieri S. Ksiazek I. Kauffmann S. (2011). Neuropathology in mice expressing mouse alpha-synuclein. PLoS One 6, e24834. 10.1371/journal.pone.0024834 21966373 PMC3180287

[B172] Rodríguez Cruz P. M. Cossins J. Beeson D. Vincent A. (2020). The neuromuscular junction in health and disease: Molecular mechanisms governing synaptic formation and homeostasis. Front. Mol. Neurosci. 13, 610964. 10.3389/fnmol.2020.610964 33343299 PMC7744297

[B173] Rozas J. L. Gómez-Sánchez L. Mircheski J. Linares-Clemente P. Nieto-González J. L. Vázquez M. E. (2012). Motorneurons require cysteine string protein-α to maintain the readily releasable vesicular pool and synaptic vesicle recycling. Neuron 74, 151–165. 10.1016/j.neuron.2012.02.019 22500637

[B174] Ruggiu M. Herbst R. Kim N. Jevsek M. Fak J. J. Mann M. A. (2008). Rescuing Z^+^ agrin splicing in *Nova* null mice restores synapse formation and unmasks a physiologic defect in motor neuron firing. Proc. Natl. Acad. Sci. U. S. A. 106, 3513–3518. 10.1073/pnas.0813112106 PMC2642475 19221030

[B175] Ruiz R. Casañas J. J. Südhof T. C. Tabares L. (2008). Cysteine string protein-alpha is essential for the high calcium sensitivity of exocytosis in a vertebrate synapse. Eur. J. Neurosci. 27, 3118–3131. 10.1111/j.1460-9568.2008.06301.x 18598257

[B176] Saiga T. Fukuda T. Matsumoto M. Tada H. Okano H. J. Okano H. (2009). Fbxo45 forms a novel ubiquitin ligase complex and is required for neuronal development. Mol. Cell Biol. 29, 3529–3543. 10.1128/MCB.00364-09 19398581 PMC2698766

[B177] Sakisaka T. Yamamoto Y. Mochida S. Nakamura M. Nishikawa K. Ishizaki H. (2008). Dual inhibition of SNARE complex formation by tomosyn ensures controlled neurotransmitter release. J. Cell Biol. 183, 323–337. 10.1083/jcb.200805150 18936251 PMC2568027

[B178] Sandrock A. W. Dryer S. E. Rosen K. M. Gozani S. N. Kramer R. Theill L. E. (1997). Maintenance of acetylcholine receptor number by neuregulins at the neuromuscular junction *in vivo* . Science 276, 599–603. 10.1126/science.276.5312.599 9110980

[B179] Santosa K. B. Keane A. M. Jablonka-Shariff A. Vannucci B. Snyder-Warwick A. K. (2018). Clinical relevance of terminal Schwann cells: An overlooked component of the neuromuscular junction. J. Neurosci. Res. 96, 1125–1135. 10.1002/jnr.24231 29536564 PMC6292684

[B180] Schmidt M. Schülke J.-P. Liebl C. Stiess M. Avrabos C. Bock J. (2011). Tumor suppressor down-regulated in renal cell carcinoma 1 (DRR1) is a stress-induced actin bundling factor that modulates synaptic efficacy and cognition. Proc. Natl. Acad. Sci. U. S. A. 108, 17213–17218. 10.1073/pnas.1103318108 21969592 PMC3193257

[B181] Schmidt N. Akaaboune M. Gajendran N. Martinez-Pena y Valenzuela I. Wakefield S. Thurnheer R. (2011). Neuregulin/ErbB regulate neuromuscular junction development by phosphorylation of α-dystrobrevin. J. Cell Biol. 195, 1171–1184. 10.1083/jcb.201107083 22184199 PMC3246897

[B182] Schoch S. Castillo P. E. Jo T. Mukherjee K. Geppert M. Wang Y. (2002). RIM1alpha forms a protein scaffold for regulating neurotransmitter release at the active zone. Nature 415, 321–326. 10.1038/415321a 11797009

[B183] Schoch S. Mittelstaedt T. Kaeser P. S. Padgett D. Feldmann N. Chevaleyre V. (2006). Redundant functions of RIM1alpha and RIM2alpha in Ca(2+)-triggered neurotransmitter release. EMBO J. 25, 5852–5863. 10.1038/sj.emboj.7601425 17124501 PMC1698877

[B184] Schwander M. Shirasaki R. Pfaff S. L. Müller U. (2004). Beta1 integrins in muscle, but not in motor neurons, are required for skeletal muscle innervation. J. Neurosci. 24, 8181–8191. 10.1523/JNEUROSCI.1345-04.2004 15371519 PMC6729792

[B185] Sealock R. Butler M. H. Kramarcy N. R. Gao K. X. Murnane A. A. Douville K. (1991). Localization of dystrophin relative to acetylcholine receptor domains in electric tissue and adult and cultured skeletal muscle. J. Cell Biol. 113, 1133–1144. 10.1083/jcb.113.5.1133 2040646 PMC2289019

[B186] Searle A. G. (1962). Mouse News Lett. 27, 34.

[B187] Seburn K. L. Nangle L. A. Cox G. A. Schimmel P. Burgess R. W. (2006). An active dominant mutation of glycyl-tRNA synthetase causes neuropathy in a Charcot-Marie-Tooth 2D mouse model. Neuron 51, 715–726. 10.1016/j.neuron.2006.08.027 16982418

[B188] Sharma M. Burré J. Bronk P. Zhang Y. Xu W. Südhof T. C. (2012). CSPα knockout causes neurodegeneration by impairing SNAP-25 function. EMBO J. 31, 829–841. 10.1038/emboj.2011.467 22187053 PMC3280561

[B189] She B.-R. Liou G.-G. Lin-Chao S. (2002). Association of the growth-arrest-specific protein Gas7 with F-actin induces reorganization of microfilaments and promotes membrane outgrowth. Exp. Cell Res. 273, 34–44. 10.1006/excr.2001.5435 11795944

[B190] Shen C. Li L. Zhao K. Bai L. Wang A. Shu X. (2018). Motoneuron Wnts regulate neuromuscular junction development. eLife 7, e34625. 10.7554/eLife.34625 30113308 PMC6128691

[B191] Shin J. Salameh J. S. Richter J. D. (2016). Impaired neurodevelopment by the low complexity domain of CPEB4 reveals a convergent pathway with neurodegeneration. Sci. Rep. 6, 29395. 10.1038/srep29395 27381259 PMC4933966

[B192] Shultz L. D. Sweet H. O. Davisson M. T. Coman D. R. (1982). Wasted, a new mutant of the mouse with abnormalities characteristic to ataxia telangiectasia. Nature 297, 402–404. 10.1038/297402a0 7078649

[B193] Sieck G. C. Prakash Y. S. (1997). Morphological adaptations of neuromuscular junctions depend on fiber type. Can. J. Appl. Physiol. 22, 197–230. 10.1139/h97-014 9189302

[B194] Snell G. D. (1955). Ducky, a new second chromosome mutation in the mouse. J. Hered. 46, 27–29. 10.1093/oxfordjournals.jhered.a106505

[B195] Srinivasan E. Chandrasekhar G. Chandrasekar P. Anbarasu K. Vickram A. S. Karunakaran R. (2021). Alpha-synuclein aggregation in Parkinson’s disease. Front. Med. (Lausanne) 8, 736978. 10.3389/fmed.2021.736978 34733860 PMC8558257

[B196] Staron R. S. Hagerman F. C. Hikida R. S. Murray T. F. Hostler D. P. Crill M. T. (2000). Fiber type composition of the vastus lateralis muscle of young men and women. J. Histochem Cytochem 48, 623–629. 10.1177/002215540004800506 10769046

[B197] Su Y. Balice-Gordon R. J. Hess D. M. Landsman D. S. Minarcik J. Golden J. (2004). Neurobeachin is essential for neuromuscular synaptic transmission. J. Neurosci. 24, 3627–3636. 10.1523/JNEUROSCI.4644-03.2004 15071111 PMC6729756

[B198] Sun Y. Ren W. Côté J.-F. Hinds P. W. Hu X. Du K. (2015). ClipR-59 interacts with Elmo2 and modulates myoblast fusion. J. Biol. Chem. 290, 6130–6140. 10.1074/jbc.M114.616680 25572395 PMC4358253

[B199] Taetzsch T. Tenga M. J. Valdez G. (2017). Muscle fibers secrete FGFBP1 to slow degeneration of neuromuscular synapses during aging and progression of ALS. J. Neurosci. 37, 70–82. 10.1523/JNEUROSCI.2992-16.2016 28053031 PMC5214636

[B200] Tanaka M. Miyoshi J. Ishizaki H. Togawa A. Ohnishi K. Endo K. (2001). Role of Rab3 GDP/GTP exchange protein in synaptic vesicle trafficking at the mouse neuromuscular junction. Mol. Biol. Cell 12, 1421–1430. 10.1091/mbc.12.5.1421 11359932 PMC34594

[B201] Thaler J. Harrison K. Sharma K. Lettieri K. Kehrl J. Pfaff S. L. (1999). Active suppression of interneuron programs within developing motor neurons revealed by analysis of homeodomain factor HB9. Neuron 23, 675–687. 10.1016/s0896-6273(01)80027-1 10482235

[B202] Vaden J. H. Bhattacharyya B. J. Chen P.-C. Watson J. A. Marshall A. G. Phillips S. E. (2015). Ubiquitin-specific protease 14 regulates c-Jun N-terminal kinase signaling at the neuromuscular junction. Mol. Neurodegener. 10, 3. 10.1186/1750-1326-10-3 25575639 PMC4417291

[B203] Valdez G. Tapia J. C. Kang H. Clemenson G. D. Gage F. H. Lichtman J. W. (2010). Attenuation of age-related changes in mouse neuromuscular synapses by caloric restriction and exercise. Proc. Natl. Acad. Sci. U. S. A. 107, 14863–14868. 10.1073/pnas.1002220107 20679195 PMC2930485

[B204] Valkova C. Albrizio M. Röder I. Schwake M. Betto R. Rudolf R. (2011). Sorting receptor Rer1 controls surface expression of muscle acetylcholine receptors by ER retention of unassembled alpha-subunits. Proc. Natl. Acad. Sci. U. S. A. 108, 621–625. 10.1073/pnas.1001624108 21187406 PMC3021032

[B205] van der Putten H. Wiederhold K. H. Probst A. Barbieri S. Mistl C. Danner S. (2000). Neuropathology in mice expressing human alpha-synuclein. J. Neurosci. 20, 6021–6029. 10.1523/JNEUROSCI.20-16-06021.2000 10934251 PMC6772584

[B206] VanSaun M. Herrera A. A. Werle M. J. (2003). Structural alterations at the neuromuscular junctions of matrix metalloproteinase 3 null mutant mice. J. Neurocytol. 32, 1129–1142. 10.1023/B:NEUR.0000021907.68461.9c 15044844

[B207] VanSaun M. Werle M. J. (2000). Matrix metalloproteinase-3 removes agrin from synaptic basal lamina. J. Neurobiol. 43, 369–9. 10.1002/1097-4695(20000905)44:3<369:aid-neu7>3.0.co;2-z 10942889

[B208] Wang B. Harrison W. Overbeek P. A. Zheng H. (2011). Transposon mutagenesis with coat color genotyping identifies an essential role for Skor2 in sonic hedgehog signaling and cerebellum development. Development 138, 4487–4497. 10.1242/dev.067264 21937600 PMC3177318

[B209] Wang B. Yang L. Wang Z. Zheng H. (2007). Amyolid precursor protein mediates presynaptic localization and activity of the high-affinity choline transporter. Proc. Natl. Acad. Sci. U. S. A. 104, 14140–14145. 10.1073/pnas.0704070104 17709753 PMC1955810

[B210] Wang P. Yang G. Mosier D. R. Chang P. Zaidi T. Gong Y.-D. (2005). Defective neuromuscular synapses in mice lacking amyloid precursor protein (APP) and APP-Like protein 2. J. Neurosci. 25, 1219–1225. 10.1523/JNEUROSCI.4660-04.2005 15689559 PMC6725967

[B211] Wang Z. Wang B. Yang L. Guo Q. Aithmitti N. Songyang Z. (2009). Presynaptic and postsynaptic interaction of the amyloid precursor protein promotes peripheral and central synaptogenesis. J. Neurosci. 29, 10788–10801. 10.1523/JNEUROSCI.2132-09.2009 19726636 PMC2757256

[B212] Weatherbee S. D. Anderson K. V. Niswander L. A. (2006). LDL-receptor-related protein 4 is crucial for formation of the neuromuscular junction. Development 133, 4993–5000. 10.1242/dev.02696 17119023

[B213] Willadt S. Nash M. Slater C. R. (2016). Age-related fragmentation of the motor endplate is not associated with impaired neuromuscular transmission in the mouse diaphragm. Sci. Rep. 6, 24849. 10.1038/srep24849 27094316 PMC4837408

[B214] Williams A. H. Valdez G. Moresi V. Qi X. McAnally J. Elliott J. L. (2009). MicroRNA-206 delays ALS progression and promotes regeneration of neuromuscular synapses in mice. Science 326, 1549–1554. 10.1126/science.1181046 20007902 PMC2796560

[B215] Wilson S. M. Bhattacharyya B. Rachel R. A. Coppola V. Tessarollo L. Householder D. B. (2002). Synaptic defects in ataxia mice result from a mutation in Usp14, encoding a ubiquitin-specific protease. Nat. Genet. 32, 420–425. 10.1038/ng1006 12368914

[B216] Wojtowicz J. M. Marin L. Atwood H. L. (1994). Activity-induced changes in synaptic release sites at the crayfish neuromuscular junction. J. Neurosci. 14, 3688–3703. 10.1523/JNEUROSCI.14-06-03688.1994 8207482 PMC6576941

[B217] Wood S. J. Slater C. R. (2001). Safety factor at the neuromuscular junction. Prog. Neurobiol. 64, 393–429. 10.1016/s0301-0082(00)00055-1 11275359

[B218] Yang J. Dominguez B. de Winter F. Gould T. W. Eriksson J. E. Lee K.-F. (2011). Nestin negatively regulates postsynaptic differentiation of the neuromuscular synapse. Nat. Neurosci. 14, 324–330. 10.1038/nn.2747 21278733 PMC3069133

[B219] Yang X. Arber S. William C. Li L. Tanabe Y. Jessell T. M. (2001). Patterning of muscle acetylcholine receptor gene expression in the absence of motor innervation. Neuron 30, 399–410. 10.1016/s0896-6273(01)00287-2 11395002

[B220] Zahavi E. E. Ionescu A. Gluska S. Gradus T. Ben-Yaakov K. Perlson E. (2015). A compartmentalized microfluidic neuromuscular co-culture system reveals spatial aspects of GDNF functions. J. Cell Sci. 128, 1241–1252. 10.1242/jcs.167544 25632161 PMC4359927

[B221] Zhang B. Luo S. Wang Q. Suzuki T. Xiong W. C. Mei L. (2008). LRP4 serves as a coreceptor of agrin. Neuron 60, 285–297. 10.1016/j.neuron.2008.10.006 18957220 PMC2743173

[B222] Zhang Y.-Q. Henderson M. X. Colangelo C. M. Ginsberg S. D. Bruce C. Wu T. (2012). Identification of CSPα clients reveals a role in dynamin 1 regulation. Neuron 74, 136–150. 10.1016/j.neuron.2012.01.029 22500636 PMC3328141

[B223] Ziller M. J. Ortega J. A. Quinlan K. A. Santos D. P. Gu H. Martin E. J. (2018). Dissecting the functional consequences of de novo DNA methylation dynamics in human motor neuron differentiation and physiology. Cell Stem Cell 22, 559–574.e9. 10.1016/j.stem.2018.02.012 29551301 PMC6535433

