# Supplementary material for: NMJ-related diseases beyond the congenital myasthenic syndromes
Source: Front Cell Dev Biol. 2023 Aug 4;11:1216726. doi: 10.3389/fcell.2023.1216726 (PMC10436495; doi:10.3389/fcell.2023.1216726)
Supplement: Supplementary file 2 [file Table1.docx]

Supplementary Material

**NMJ-related diseases beyond the congenital myasthenic syndromes**

**Alejandra Navarro-Martínez, Cristina Vicente-García*, Jaime J. Carvajal**

**Table S1**. Mouse models associated with NMJ-related abnormalities registered at the Mouse Genome Informatics MGI database (September, 2022). AEP: Abnormal Endplate Potential; AMEP: Abnormal Miniature Endplate Potential; ANSM: Abnormal Neuromuscular Synapse Morphology; FNSPrD: Failure of Neuromuscular Synapse Presynaptic Differentiation; FNSPoD: Failure of Neuromuscular Synapse Postsynaptic Differentiation.

| **Allele composition** | **Genetic background** | **Annotated MGI term** |
| --- | --- | --- |
| Adarb1^tm1.1Skwa^/Adarb1^tm1.1Skwa^  Tg(SLC18A3-cre)KMisa/0 | C57BL/6 | ANSM |
| Afg3l2^par^/Afg3l2^par^ | Not Specified | ANSM AMEP |
| Agrn^Gt(Ex192)Byg^/Agrn^Gt(Ex192)Byg^ | 129P2/OlaHsd * C57BL/6 | ANSM |
| Agrn^nmf380^/Agrn^nmf380^ | C57BL/6J-Agrn^nmf380^/J | ANSM |
| Agrn^nmf380^/Agrn^nmf380^ | C57BL/6J * FVB/N | ANSM |
| Agrn^nmf380^/Agrn^nmf380^ | C57BL/6J * DBA | ANSM |
| Agrn^nmf380^/Agrn^nmf380^ | BALB/c * C57BL/6J | ANSM |
| Agrn^nmf380^/Agrn^nmf380^ | C57BL/6J * CAST | ANSM |
| Agrn^tm1Jrs^/Agrn^tm1Jrs^ | 129S1/Sv * 129X1/SvJ | ANSM |
| Agrn^tm1Rwb^/Agrn^tm1Rwb^ Tg(Pax3-cre)1Joe/0 | 129S1/Sv * 129X1/SvJ * C57BL/6 * SJL | ANSM |
| Agrn^tm3Jrs^/Agrn^tm3Jrs^ | 129S1/Sv * 129X1/SvJ * C57BL/6 | ANSM |
| Agrn^tm4Jrs^/Agrn^tm4Jrs^ | Not Specified | FNSPrD |
| Agrn^tm4Jrs^/Agrn^tm4Jrs^ Chrna1^tm1Klee^/Chrna1^tm1Klee^ Chrna7^tm1Bay^/Chrna7^tm1Bay^ | 129S4/SvJae * 129S7/SvEvBrd * C57BL/6 | FNSPrD |
| Als2^tm1Jei^/Als2^tm1Jei^ | 129P2/OlaHsd * C57BL/6J | ANSM |
| Aplp2^tm1Dbo^/Aplp2^tm1Dbo^ App^tm1.1Zhe^/App^tm1.1Zhe^ Chat^tm2(cre)Lowl^/Chat^+^ | 129S6/SvEvTac * 129S7/SvEvBrd * C57BL/6J | ANSM |
| Aplp2^tm1Dbo^/Aplp2^tm1Dbo^ App^tm1.1Zhe^/App^tm1.1Zhe^ Tg(Ckmm-cre)5Khn/0 | 129S7/SvEvBrd * C57BL/6J * FVB | ANSM AMEP |
| Aplp2^tm1Dbo^/Aplp2^tm1Dbo^ App^tm1.1Zhe^/App^tm1.1Zhe^ Tg(Nes-cre)1Kln/0 | 129S7/SvEvBrd * C57BL/6 * C57BL/6J * SJL | ANSM AMEP |
| Aplp2^tm1Dbo^/Aplp2^tm1Dbo^ App^tm1.2Zhe^/App^tm1.2Zhe^ | 129S7/SvEvBrd * C57BL/6J | ANSM |
| Aplp2^tm1Dbo^/Aplp2^tm1Dbo^ App^tm1Dbo^/App^tm1Dbo^ | 129S7/SvEvBrd * C57BL/6J | ANSM |

**Table S1.** (*continued*)

| **Allele composition** | **Genetic background** | **Annotated MGI term** |
| --- | --- | --- |
| Aplp2^tm1Dbo^/Aplp2^tm1Dbo^ App^tm1Dbo^/App^tm3.1Zhe^ | 129/Sv * 129X1/SvJ * C57BL/6 | ANSM |
| Aplp2^tm1Dbo^/Aplp2^tm1Dbo^ App^tm2.1Zhe^/App^tm2.1Zhe^ | 129S1/Sv * 129S7/SvEvBrd * 129X1/SvJ | ANSM |
| Aplp2^tm1Dbo^/Aplp2^tm1Dbo^ App^tm3.1Zhe^/App^tm3.1Zhe^ | 129/Sv * 129X1/SvJ * C57BL/6 | ANSM |
| Aplp2^tm1Dbo^/Aplp2^tm1Dbo^ Lrp4^tm1Her^/Lrp4^tm1Her^ | 129S6/SvEvTac * 129S7/SvEvBrd * C57BL/6J | ANSM |
| App^tm1Dbo^/App^tm1Dbo^ Lrp4^tm1Her^/Lrp4^tm1Her^ | 129S6/SvEvTac * 129S7/SvEvBrd * C57BL/6J | ANSM |
| Atp2b1^Tg(Thy1-CHMP2B*)1Rene^/Atp2b1^+^ | C57BL/6 * DBA/2 * FVB/N | ANSM |
| Atp2b1^Tg(Thy1-CHMP2B*)1Rene^/ Atp2b1^Tg(Thy1-CHMP2B*)1Rene^ | C57BL/6 * DBA/2 * FVB/N | ANSM |
| Atp7a^tm1.1Mjp^/Y Mnx1^tm4(cre)Tmj^/Mnx1^+^ | 129S1/Sv * C57BL/6 | ANSM |
| Basp1^tm1Crni^/Basp1^tm1Crni^ | Not Specified | ANSM |
| Basp1^tm2(Gap43)Crni^/Basp1^tm2(Gap43)Crni^ | Not Specified | ANSM |
| Borcs5^em1Jusb^/Borcs5^em1Jusb^ | C57BL/6-Borcs5^em1Jusb^ | ANSM |
| Cacna1a^tm1Maag^/Cacna1a^tm1Maag^ | 129P2/OlaHsd * C57BL/6J | AMEP |
| Cacna2d2^du^/Cacna2d2^du^ | C3H * DBA/2J | ANSM AMEP |
| Cacnb4^lh^/Cacnb4^lh^ | BALB/cGn * C3H/HeSnJ * C57BL/6JEi | ANSM |
| Cacng2^stg^/Cacng2^stg^ | A/J * C3HeB/FeJ * C57BL/6J | ANSM |
| Chat/Slc18a3^tm1.1Vpra^/Chat^+^ | 129S4/SvJae * C57BL/6J | AMEP |
| Chat/Slc18a3^tm1Vpra^/Chat/Slc18a3^tm1Vpra^ | 129S4/SvJae * C57BL/6J | AMEP |
| Chat/Slc18a3^tm1Vpra^/Chat/Slc18a3^tm1.1Vpra^ | 129S4/SvJae * C57BL/6J | AMEP |
| Chat/Slc18a3^tm1.1Vpra^/Chat/Slc18a3^tm1.1Vpra^ | 129S4/SvJae * C57BL/6J | ANSM AMEP |
| Chat/Slc18a3^tm1.1Vpra^/ Slc18a3^+^ | 129S4/SvJae * C57BL/6J | AMEP |
| Chat^tm1.1Jrs^/Chat^tm1.1Jrs^ | Not Specified | ANSM |
| Chat^tm1Fhg^/Chat^tm1Fhg^ | 129S2/SvPas * C57BL/6 | ANSM |
| Chat^tm1Jrs^/Chat^tm1Jrs^ Tg(CAG-cre/Esr1*)1Lbe/0 | 129S1/Sv * 129X1/SvJ * C57BL/6 * SJL | ANSM |
| Chat^tm2(cre)Lowl^/Chat^+^ Mapt^tm2(FUS)Neas^/Mapt^+^ | B6.129-Mapt^tm2(FUS)Neas^ Chat^tm2(cre)Lowl^ | ANSM |
| Chat^tm2(cre)Lowl^/Chat^+^ Mapt^tm3(FUS)Neas^/Mapt^+^ | B6.129-Mapt^tm3(FUS)Neas^ Chat^tm2(cre)Lowl^ | ANSM |
| Chchd10^em8Lutzy^/Chchd10^+^ | 57BL/6NJ-Chchd10^em8Lutzy^/J | ANSM |
| Chrna1^tm1Klee^/Chrna1^tm1Klee^ | 129S4/SvJae * C57BL/6 | ANSM AEP AMEP |
| Chrna7^tm1Bay^/Chrna7^tm1Bay^ | 129S7/SvEvBrd | FNSPrD |
| Chrnb1^tm1Sjb^/Chrnb1^tm1Sjb^ | Not Specified | ANSM AMEP |

**Table S1.** (*continued*)

| **Allele composition** | **Genetic background** | **Annotated MGI term** |
| --- | --- | --- |
| Chrne^tm1Jrs^/Chrne^tm1Jrs^ | 129S1/Sv * 129X1/SvJ * C57BL/6 | ANSM |
| Chrne^tm1Vwi^/Chrne^tm1Vwi^ | 129P2/OlaHsd * C57BL/6 | ANSM AMEP |
| Chrne^tm2Vwi^/Chrne^tm2Vwi^ | 129P2/OlaHsd * C57BL/6 | ANSM AMEP |
| Chrng^tm1(Chrne)Mknn^/Chrng^tm1(Chrne)Mknn^ | 129/Sv * C57BL/6 | AEP |
| Chrng^tm1Mtak^/Chrng^tm1Mtak^ | 129X1/SvJ * C57BL/6 | ANSM AMEP |
| Chrng^tm2(Chrne)Vwi^/Chrng^tm2(Chrne)Vwi^ | C57BL/6 | ANSM AMEP |
| Clip3^tm1.1Ics^/Clip3^tm1.1Ics^ | C57BL/6 | ANSM |
| Clp1^tm1.1Pngr^/Clp1^tm1.1Pngr^ | CBA.Cg-Clp1^tm1.1Pngr^ | ANSM |
| Clp1^tm1.1Pngr^/Clp1^tm1.1Pngr^ | B6.Cg-Clp1^tm1.1Pngr^ | ANSM |
| Col13a1^tm1Pih^/Col13a1^tm1Pih^ | 129S1/Sv * 129X1/SvJ | AEP |
| Col13a1^tm2Pih^/Col13a1^tm2Pih^ | 129S6/SvEvTac * C57BL/6 | AEP |
| Col13a1^tm3.1Pih^/Col13a1^tm3.1Pih^ | 129S1/Sv * 129X1/SvJ * C57BL/6 | ANSM AEP AMEP |
| Col13a1^tm3.1Pih^/Col13a1^tm3.1Pih^ | 129S1/Sv * 129X1/SvJ | ANSM |
| Col13a1^tm4.1Pih^/Col13a1^tm4.1Pih^ | C57BL/6 | ANSM AEP AMEP |
| Colq^tm1Jrs^/Colq^tm1Jrs^ | 129S1/Sv * 129X1/SvJ | ANSM |
| Colq^tm2Jrs^/Colq^tm2Jrs^ | 129S1/Sv * 129X1/SvJ | ANSM |
| Cpeb4^tm1a(EUCOMM)Wtsi^/Cpeb4^tm1a(EUCOMM)Wtsi^ | C57BL/6 * C57BL/6Ntac | ANSM |
| Csnk2b^tm1.1Bb^/Csnk2b^tm1.1Bb^ Tg(ACTA1-cre)79Jme/? | 129S2/SvPasCrl * C57BL/6J * SJL | ANSM AMEP |
| Dag1^tm2Kcam^/Dag1^tm2Kcam^ Tg(Pax3-cre)1Joe/0 | 129S1/Sv * 129X1/SvJ * C57BL/6 * SJL | ANSM |
| Dag1^tm4.1Kcam^/Dag1^tm4.1Kcam^ | 129S1/Sv * 129X1/SvJ | ANSM |
| Dctn1^tm1Cai^/Dctn1^+^ | 129X1/SvJ * C57BL/6 * FVB/N | ANSM |
| Dctn1^tm2.1Cai^/Dctn1^tm2.1Cai^ Tg(Thy1-cre)1Vln/? | 129S4/SvJaeSor * 129X1/SvJ * C57BL/6 * FVB/N | ANSM |
| Dhtkd1^tm1Zgwg^/Dhtkd1^tm1Zgwg^ | C57BL/6 | ANSM |
| Dmd^mdx^/Y | C57BL/10ScSn | ANSM |
| Dmd^mdx^/Y Dtna^tm1Jrs^/Dtna^tm1Jrs^ | 129X1/SvJ * C57BL/10ScSn | ANSM |
| Dmd^mdx^/Y Dtna^tm1Jrs^/Dtna^tm1Jrs^ Utrn^tm1Jrs^/Utrn^tm1Jrs^ | 129S1/Sv * 129X1/SvJ * C57BL/10ScSn | ANSM |
| Dmd^mdx^/Dmd^mdx^ | C57BL/10ScSn | ANSM |
| Dmd^mdx^/Dmd^mdx^ Utrn^tm1Jrs^/Utrn^tm1Jrs^ | 129S1/Sv * 129X1/SvJ * C57BL/10ScSn | ANSM |
| Dmd^mdx^/Dmd^mdx^ Utrn^tm1Ked^/Utrn^tm1Ked^ | 129S1/Sv * 129X1/SvJ * C57BL/6 * C57BL/10ScSn * DBA | ANSM |
| Dnajc5^tm1Sud^/Dnajc5^tm1Sud^ | 129S6/SvEvTac | ANSM AEP |
| Dnm2^tm2.1Ics^/Dnm2^+^ | C57BL/6J | ANSM |

**Table S1.** (*continued*)

| **Allele composition** | **Genetic background** | **Annotated MGI term** |
| --- | --- | --- |
| Dnmt3a^tm1Jae^/Dnmt3a^tm1Jae^ Tg(Nes-cre)1Atp/0 | 129S4/SvJae * C57BL/6 * FVB/N | ANSM |
| Dok7^tm1Yyam^/Dok7^tm1Yyam^ | 129P2/OlaHsd * C57BL/6 | ANSM |
| Dok7^tm2Yyam^/Dok7^tm2Yyam^ | 129P2/OlaHsd * C57BL/6J | ANSM |
| Dst^Tg4^/Dst^Tg4^ | C57BL/6 * CD-1 | ANSM |
| Dtna^tm1Jrs^/Dtna^tm1Jrs^ | 129X1/SvJ * C57BL/6 | ANSM |
| Dtna^tm1Jrs^/Dtna^tm1Jrs^ Utrn^tm1Jrs^/Utrn^tm1Jrs^ | 129S1/Sv * 129X1/SvJ | ANSM |
| Dync1h1^tm1.1Sjki^/Dync1h1^+^ | 129 * 129S1/SvImJ * C57BL/6 * C57BL/6J | ANSM |
| Ecel1^tm1Hiki^/Ecel1^tm1Hiki^ | B6.129-Ecel1^tm1Hiki^ | ANSM |
| Ecel1^tm1Hiki^/Ecel1^tm1Hiki^ Tg(Hlxb9-GFP)1Tmj/0 | 129S1/Sv * 129X1/SvJ * C57BL/6 * CBA | ANSM |
| Ednrb^s-1Acrg^/Ednrb^s-9ThW^ | 101/Rl * C3H/Rl * C57BL/6J | ANSM |
| Eef1a2^wst^/Eef1a2^wst^ | HRS/J | ANSM AEP |
| Egr2^tm2Jmi^/Egr2^tm2Jmi^ | B6.Cg-Egr2^tm2Jmi^ | ANSM |
| Erbb2^tm3(Erbb2)Mul^/Erbb2^tm1Mll^ Tg(ACTA1-cre)1Mll/0 | 129S1/Sv * 129X1/SvJ * C57BL/6 | ANSM |
| Fat1^Gt(KST249)Byg^/Fat1^Gt(KST249)Byg^ Tg(Myl1-lacZ)1Ibdml/0 | 129P2/OlaHsd | ANSM |
| Fat1^tm1.2Fhel^/Fat1^tm1.2Fhel^ Tg(Myl1-lacZ)1Ibdml/0 | 129S6/SvEvTac * BALB/cJ * C57BL/6J * SJL | ANSM |
| Fbxo45^tm1Kei^/Fbxo45^tm1Kei^ | B6.129P2-Fbxo45^tm1Kei^ | ANSM |
| Fgfbp1^tm1Gvdz^/Fgfbp1^tm1Gvdz^ | 129S5/SvEvBrd | ANSM |
| Fgfbp1^tm1Gvdz^/Fgfbp1^tm1Gvdz^ Tg(SOD1*G93A)1Gur/0 Tg(Thy1-YFP)16Jrs/0 | 129S5/SvEvBrd * C57BL/6J * CBA * SJL | ANSM |
| Fgfbp1^tm1Gvdz^/Fgfbp1^tm1Gvdz^ Tg(Thy1-YFP)16Jrs/0 | 129S5/SvEvBrd * C57BL/6J * CBA | ANSM |
| Fus^tm1.1Emcf^/Fus^+^ | C3H * C57BL/6J * C57BL/6N | ANSM |
| Fus^tm1a(EUCOMM)Wtsi^/Fus^tm1c(EUCOMM)Wtsi^ Mapt^tm3.1(FUS)Neas^/Mapt^+^ Ndor1^Tg(UBC-cre/ERT2)1Ejb^/0 | 129 * C3H * C57BL/6 * C57BL/6N | ANSM |
| Gabpa^tm1Pjh^/Gabpa^tm1Pjh^ Tg(ACTA1-cre)79Jme/0 | 129X1/SvJ * C57BL/6 * SJL | ANSM |
| Gars^C201R^/Gars^+^ | BALB/cAnN * C3H/HeH * C57BL/6J | ANSM |
| Gars^C201R^/Gars^+^ Nrcam^m1J^/Nrcam^m1J^ | BALB/cAnN * C3H/HeH * C57BL/6J | ANSM |
| Gars^C201R^/Gars^+^ Scn8a^m10J^/Scn8a^+^ | BALB/cAnN * C3H/HeJ * FVB/NJ | ANSM |
| Gars^C201R^/Gars^+^ Tg(Thy1-YFP)16Jrs/? | BALB/cAnN * C3H/HeH * C57BL/6J * CBA | ANSM |

**Table S1.** (*continued*)

| **Allele composition** | **Genetic background** | **Annotated MGI term** |
| --- | --- | --- |
| Gars^C201R^/Gars^C201R^ Tg(CAG-GARS)DRwb/0 | BALB/cAnN * C3H/HeH * C57BL/6J * FVB/N | ANSM |
| Gars^C201R^/Gars^Gt(XM256)6Byg^ Tg(CAG-GARS)DRwb/0 | 129P2/OlaHsd * BALB/cAnN * C3H/HeH * C57BL/6J * FVB/N | ANSM |
| Gars^C201R^/Gars^Nmf249^ | BALB/cAnN * C3H/HeH * C57BL/6J | ANSM |
| Gars^C201R^/Gars^Nmf249^ Tg(CAG-GARS)DRwb/0 | BALB/cAnN * C3H/HeH * C57BL/6J * FVB/N | ANSM |
| Gars^em1Rwb^/Gars^em2Rwb^ | C57BL/6NJ * FVB/NJ | ANSM |
| Gars^Nmf249^/Gars^+^ | C57BL/6J-Gars^Nmf249^/J | ANSM |
| Gars^Nmf249^/Gars^+^ | C57BL/6J * CAST/Ei | ANSM |
| Gars^Nmf249^/Gars^+^ Scn8a^m10J^/Scn8a^+^ | C57BL/6J * FVB/NJ | ANSM |
| Gars^Nmf249^/Gars^+^ Tg(CAG-GARS)DRwb/0 | C57BL/6J * CAST/Ei * FVB/N | ANSM |
| Gars^Nmf249^/Gars^+^ Tg(Thy1-YFP)16Jrs/? | C57BL/6J * CAST/Ei * CBA/J | ANSM AEP |
| Gas7^tm1Slch^/Gas7^tm1Slch^ | B6.129-Gas7^tm1Slch^ | ANSM |
| Gdap1^tm1.2Geno^/Gdap1^tm1.2Geno^ | 129 * C57BL/6 | ANSM |
| Gfpt1^tm1c(EUCOMM)Wtsi^/Gfpt1^tm1c(EUCOMM)Wtsi^ Tg(Ckmm-cre)5Khn/0 | C57BL/6J * C57BL/6N * SJL/J | ANSM |
| Gphn^tm1Jrs^/Gphn^tm1Jrs^ | 129S1/Sv * 129X1/SvJ * C57BL/6 | ANSM |
| Gt(ROSA)26Sor^tm1.1(CAG-PLS3,-GFP)Bwir^/ Gt(ROSA)26Sor^+^ Smn1^tm1Hung^/Smn1^+^ Tg(SMN2)2Hung/0 | B6N.Cg-Gt(ROSA)26Sor^tm1.1(CAG-PLS3,-GFP)Bwir^ Smn1^tm1Hung^ Tg(SMN2)2Hung | ANSM |
| Gt(ROSA)26Sor^tm1.1(CAG-PLS3,-GFP)Bwir^/ Gt(ROSA)26Sor^+^ Smn1^tm1Hung^/Smn1^tm1Hung^ Tg(SMN2)2Hung/0 | B6N.Cg-Gt(ROSA)26Sor^tm1.1(CAG-PLS3,-GFP)Bwir^ Smn1^tm1Hung^ Tg(SMN2)2Hung | ANSM |
| Gt(ROSA)26Sor^tm407(H1/tetO-RNAi:Large)Arte^/ Gt(ROSA)26Sor^+^ | B6.Cg-Gt(ROSA)26Sor^tm407(H1/tetO-RNAi:Large)Arte^ | ANSM |
| Hspg2^tm1.1Soni^/Hspg2^tm1.1Soni^ | 129S/SvEv * C57BL/6 | ANSM AEP AMEP |
| Hspg2^tm1Nid^/Hspg2^tm1Nid^ | 129X1/SvJ * C57BL/6 | ANSM |
| Hspg2^tm1Soni^/Hspg2^tm1Soni^ | 129S/SvEv * DBA/2J | ANSM |
| Ighmbp2^em1Cll^/Ighmbp2^em1Cll^ | FVB/NJ-Ighmbp2^em1Cll^ | ANSM |
| Ighmbp2^nmd-2J^/Ighmbp2^nmd-2J^ | B6.BKS-Ighmbp2^nmd-2J^/J | ANSM |
| Itgb1^tm1Lscd^/Itgb1^tm1Ref^ Tg(PLAT-cre)116Sdu/0 | Not Specified | ANSM |
| Kalrn^tm2.1Npl^/Kalrn^tm2.1Npl^ | B6.Cg-Kalrn^tm2.1Npl^ | ANSM |
| Ky^ky^/Ky^ky^ | Not Specified | ANSM |

**Table S1.** (*continued*)

| **Allele composition** | **Genetic background** | **Annotated MGI term** |
| --- | --- | --- |
| Lama4^tm1Ktry^/Lama4^tm1Ktry^ Lama5^tm2Jhm^/Lama5^tm2Jhm^ Tg(ACTA1-cre)1Mll/0 | 129S1/Sv * 129X1/SvJ | ANSM |
| Lama5^tm1Jhm^/Lama5^tm1Jhm^ Tg(ACTB-Lama5/LAMA1)1Jhm/0 | 129S1/Sv * 129X1/SvJ * C57BL/6 * CBA | ANSM |
| Lama5^tm2Jhm^/Lama5^tm2Jhm^ Tg(ACTA1-cre)1Mll/0 | 129S1/Sv * 129X1/SvJ | ANSM |
| Lamb2^tm1Jrs^/Lamb2^tm1Jrs^ | 129S1/Sv * 129X1/SvJ | ANSM AMEP |
| Lgals1^tm1Rob^/Lgals1^tm1Rob^ | 129S/SvEv | ANSM |
| Lrp4^mitt^/Lrp4^mitt^ | C57BL/6 | ANSM FNSPrD FNSPoD |
| Lrp4^mte^/Lrp4^mte^ | C57BL/6 | ANSM FNSPrD FNSPoD |
| Lrp4^tm1.1Line^/Lrp4^tm1.1Line^ Mnx1^tm4(cre)Tmj^/Mnx1^+^ Tg(ACTA1-cre)79Jme/0 | 129S/SvEv * 129S1/Sv * C57BL/6 * C57BL/6J * SJL | ANSM |
| Lrp4^tm1.1Line^/Lrp4^tm1.1Line^ Tg(ACTA1-cre)79Jme/0 | 129S/SvEv * C57BL/6 * C57BL/6J * SJL | ANSM AEP AMEP |
| Lrp4^tm1Her^/Lrp4^tm1Her^ | 129S/SvEvBrd * 129S6/SvEvTac * C57BL/6J | ANSM |
| Lrp4^tm2Her^/Lrp4^tm2Her^ | 129S/SvEv * C57BL | ANSM |
| Lrp4^tm2Her^/Lrp4^tm2Her^ | 129S/SvEvBrd * C57BL/6J | ANSM FNSPrD FNSPoD |
| Lrsam1^Gt(RRK461)Byg^/Lrsam1^+^ | 129P2/OlaHsd | ANSM |
| Lrsam1^Gt(RRK461)Byg^/Lrsam1^Gt(RRK461)Byg^ | 129P2/OlaHsd | ANSM |
| Madd^tm1Ytk^/Madd^tm1Ytk^ | 129X1/SvJ * C57BL/6 * DBA/2 | ANSM |
| Mapt^tm2.1(FUS)Neas^/Mapt^+^ | B6J.129P2(129S)-Mapt^tm2.1(FUS)Neas^ | ANSM |
| Mapt^tm3.1(FUS)Neas^/Mapt^+^ | B6J.129P2(129S)-Mapt^tm3.1(FUS)Neas^ | ANSM |
| Mapt^tm3.1(FUS)Neas^/Mapt^tm3.1(FUS)Neas^ | B6J.129P2(129S)-Mapt^tm3.1(FUS)Neas^ | ANSM |
| Matr3^em1Tcp^/Matr3^em1Tcp^ | C57BL/6J-Matr3^em1Tcp^ | ANSM |
| Mbnl1^tm1Sws^/Mbnl1^tm1Sws^ Mbnl2^tm1.1Sws^/Mbnl2^+^ | 129S1/Sv * 129S1/SvImJ * C57BL | ANSM |
| Mdga2^Tg(Prnp-PFN1*G118V)838Kiaei^/Mdga2^+^ | C57BL/6N | ANSM |
| Meox2^tm1(cre)Sor^/Meox2^+^ Tg(CAG-lacZ,-FUS*R521G,-EGFP)682Gyu/0 | 129S4/SvJaeSor * C57BL/6 | ANSM |
| Meox2^tm1(cre)Sor^/Meox2^+^ Tg(CAG-lacZ,-FUS,-EGFP)629Gyu/0 | 129S4/SvJaeSor * C57BL/6 | ANSM |
| Met^tm1Cpo^/Met^tm1Sst^ Tg(Nes-cre)1Kln/0 | 129P2/OlaHsd * 129S1/Sv * 129X1/SvJ * C57BL/6 * SJL | ANSM |
| Mir206^tm1Eno^/Mir206^tm1Eno^ Tg(SOD1*G93A)^dl^1Gur/0 | 129S/SvEv * C57BL/6 * SJL | ANSM |

**Table S1.** (*continued*)

| **Allele composition** | **Genetic background** | **Annotated MGI term** |
| --- | --- | --- |
| Mir218-1^em1Slp^/Mir218-1^em1Slp^ Mir218-2^em1Slp^/Mir218-2^em1Slp^ | Not Specified | FNSPrD |
| Mmp3^tm1Mrl^/Mmp3^tm1Mrl^ | 129S7/SvEvBrd or 129S7/SvEvBrd * C57BL/6 | ANSM AMEP |
| Mnx1^tm4(cre)Tmj^/Mnx1^+^ | 129S1/Sv | ANSM |
| Mnx1^tm4(cre)Tmj^/Mnx1^+^ Mycbp2^tm1Adia^/Mycbp2^tm1Adia^ | 129S1/Sv * C57BL/6 | ANSM |
| Musk^tm1.1Vwi^/Musk^tm2Vwi^ | Not Specified | ANSM AEP AMEP |
| Musk^tm1.2Ics^/Musk^tm1.2Ics^ | BALB/cN * C57BL/6NTac | ANSM |
| Musk^tm1Gdy^/Musk^tm1Gdy^ | 129P2/OlaHsd * C57BL/6 | ANSM FNSPrD FNSPoD |
| Musk^tm1Vwi^/Musk^tm1.1Vwi^ Tg(Ckmm-cre)5Khn/0 | C57BL/6J * FVB | ANSM |
| Musk^tm1Vwi^/Musk^tm1Vwi^ Tg(Ckmm-cre)5Khn/0 | C57BL/6J * FVB | ANSM |
| Mycbp2^tm1.1Adia^/Mycbp2^tm1.1Adia^ | C57BL/6 * FVB/N | ANSM |
| Myf5^tm3(cre)Sor^/Myf5^+^ Spin1^tm1.1Rosc^/Spin1^tm1.1Rosc^ | 129S4/SvJaeSor * C57BL/6N * C57BL/6NTac | ANSM |
| Nbea^Tg(GH1)240BNec^/Nbea^Tg(GH1)240BNec^ | C57BL/6 * SJL | AMEP |
| Nemf^em8Cx^/Nemf^em8Cx^ | C57BL/6J-Nemf^em8Cx^/Cx | ANSM |
| Nemf^pdft^/Nemf^pdft^ | B6(C3)-Nemf^pdft^/Cx | ANSM |
| Nemf^tvrm116^/Nemf^tvrm116^ | C57BL/6J-Nemf^tvrm116^/PjnCx | ANSM |
| Nes^tm1Nagy^/Nes^tm1Nagy^ | 129S6/SvEvTac * C57BL/6NCr or 129S6/SvEvTac * C57BL/6NCr * ICR | ANSM |
| Nkx6-2^tm2(cre)Ercs^/Nkx6-2^+^ Ret^tm1.1Pern^/Ret^tm1.1Pern^ | 129P2/OlaHsd * C57BL/6 * SJL | ANSM |
| nmf67/nmf67 | C57BL/6J-nmf67/J | ANSM |
| Nrcam^m1J^/Nrcam^m1J^ Sh3tc2^m1J^/Sh3tc2^m1J^ | B6.Cg-Nrcam^m1J^ Sh3tc2^m1J^/GrsrRwb | ANSM |
| Nrg1^tm1Leth^/Nrg1^+^ | 129S1/Sv * C57BL/6 | ANSM AEP AMEP |
| Nrg1^tm1Cbm^/Nrg1^tm3Cbm^ Isl2^tm1Arbr^/Isl2^+^ | 129P2/OlaHsd * 129S7/SvEvBrd * C57BL/6J | ANSM |
| Olig2^tm1(cre)Tmj^/Olig2^+^ Smn1^tm1Jme^/Smn1^tm1Msd^ Grm7^Tg(SMN2)89Ahmb^/Grm7^Tg(SMN2)89Ahmb^ | 129 * 129P2/OlaHsd * FVB/N | ANSM AMEP |
| Pex10^m1Nisw^/Pex10^m1Nisw^ | 129S1.B6-Pex10^m1Nisw^ | ANSM AEP |
| Plaa^em1Pmi^/Plaa^em1Pmi^ | C57BL/6J-Plaa^em1Pmi^ | ANSM AMEP |
| Plaa^em1Pmi^/Plaa^tm1(NCOM)Cmhd^ | C57BL/6J * C57BL/6NTac or C57BL/6J * C57BL/6NTac * CD-1 | ANSM |
| Prkcq^tm1Litt^/Prkcq^+^ | B6.129P2-Prkcq^tm1Litt^ | ANSM |

**Table S1.** (*continued*)

| **Allele composition** | **Genetic background** | **Annotated MGI term** |
| --- | --- | --- |
| Prkcq^tm1Litt^/Prkcq^tm1Litt^ | B6.129P2-Prkcq^tm1Litt^ | ANSM AEP |
| Psmf1^tm1c(EUCOMM)Hmgu^/Psmf1^tm1c(EUCOMM)Hmgu^ Mnx1^tm4(cre)Tmj^/Mnx1^+^ | 129S1/Sv * C57BL/6J * C57BL/6N | ANSM |
| Rab18^Gt(EUCE0233a03)Hmgu^/ Rab18^Gt(EUCE0233a03)Hmgu^ | 129P2/OlaHsd * C57BL/6J | ANSM |
| Rapsn^em1Gan^/Rapsn^em1Gan^ | C57BL/6J-Rapsn^em1Gan^ | ANSM AMEP |
| Rapsn^em1Gan^/Rapsn^tm1Jrs^ | 129S1/Sv * 129X1/SvJ * C57BL/6J | ANSM AMEP |
| Rapsn^tm1Jrs^/Rapsn^tm1Jrs^ | 129S1/Sv * 129X1/SvJ | ANSM FNSPoD |
| Rer1^Gt(RRN159)Byg^/Rer1^+^ | 129P2/OlaHsd | ANSM |
| Ret^tm1.2Pern^/Ret^tm1.2Pern^ | 129P2/OlaHsd * BALB/c * C57BL/6 * SJL | ANSM |
| Rims1^tm1Sud^/Rims1^tm1Sud^  Rims2^tm1.1Schc^/Rims2^tm1.1Schc^ | 129P2/OlaHsd * 129S1/Sv * 129S4/SvJae * 129X1/SvJ | AEP AMEP |
| Rnd3^Gt(OST364657)Lex^/Rnd3^Gt(OST364657)Lex^ | 129S5/SvEvBrd * C57BL/6J | ANSM |
| Scn8a^med^/Scn8a^med^ | PCT | ANSM AEP AMEP |
| Scyl1^mdf^/Scyl1^mdf^ | B6C3Fe a/a-Scyl1^mdf^/J | ANSM |
| Setx^tm1.1Als^/Setx^+^ | C57BL/6J | ANSM |
| Skor2^Tn(sb-Tyr)1799B.CA3Ove^/ Skor2^Tn(sb-Tyr)1799B.CA3Ove^ | FVB/N | ANSM |
| Skor2^Tn(sb-Tyr)1799B.CA7Ove^/ Skor2^Tn(sb-Tyr)1799B.CA7Ove^ | FVB/N | ANSM |
| Slc18a3^tm1Mca^/Slc18a3^+^ | 129S4/SvJae * C57BL/6J | AMEP |
| Slc18a3^tm1Mca^/Slc18a3^tm1Mca^ | 129S4/SvJae * C57BL/6J | AEP AMEP |
| Slc5a7^tm1Rbl^/Slc5a7^tm1Rbl^ | 129S6/SvEvTac * C57BL/6 | ANSM |
| Slc6a5^m1J^/Slc6a5^m1J^ | 129S2/SvPas * NOD | ANSM |
| Slc16a3^tm1Tac^/Slc16a3^tm1Tac^ | B6NTac.Cg-Slc16a3^tm1Tac^/Tac | ANSM |
| Slc25a46^atc^/Slc25a46^atc^ | B6.Cg-Slc25a46^atc^ | ANSM |
| Smn1^tm1Cdid^/Smn1^tm1Cdid^ Grm7^Tg(SMN2)89Ahmb^/Grm7^+^ | 129 * C57BL/6 * FVB | ANSM |
| Smn1^tm1Hung^/Smn1^tm1Hung^ Tg(SMN2)2Hung/0 | B6N.Cg-Smn1^tm1Hung^ Tg(SMN2)2Hung | ANSM |
| Smn1^tm1Hung^/Smn1^tm1Hung^ Tg(SMN2)2Hung/0 | FVB.Cg-Smn1^tm1Hung^ Tg(SMN2)2Hung/J | ANSM |
| Smn1^tm1Hung^/Smn1^tm1Hung^ Tg(SMN2)2Hung/0 | 129P2/OlaHsd * C57BL/6N * FVB/N | ANSM AEP |
| Smn1^tm1Msd^/Smn1^tm1Msd^ Grm7^Tg(SMN2)89Ahmb^/Grm7^Tg(SMN2)89Ahmb^ | FVB.Cg-Grm7^Tg(SMN2)89Ahmb^ Smn1^tm1Msd^/J | ANSM |
| Smn1^tm1Msd^/Smn1^tm1Msd^ Grm7^Tg(SMN2)89Ahmb^/Grm7^Tg(SMN2)89Ahmb^ Tg(SMN2*delta7)4299Ahmb/ Tg(SMN2*delta7)4299Ahmb | FVB.Cg-Grm7^Tg(SMN2)89Ahmb^ Smn1^tm1Msd^ Tg(SMN2*delta7)4299Ahmb/J | ANSM |

**Table S1.** (*continued*)

| **Allele composition** | **Genetic background** | **Annotated MGI term** |
| --- | --- | --- |
| Smn1^tm1Msd^/Smn1^tm1Msd^ Grm7^Tg(SMN2)89Ahmb^/Grm7^Tg(SMN2)89Ahmb^ Tg(SMN2*delta7)4299Ahmb/ Tg(SMN2*delta7)4299Ahmb | FVB.Cg-Grm7^Tg(SMN2)89Ahmb^ Smn1^tm1Msd^ Tg(SMN2*delta7)4299Ahmb | ANSM |
| Smn1^tm1Msd^/Smn1^tm1Msd^ Tg(SMN1*A2G)2023Ahmb/0 Grm7^Tg(SMN2)89Ahmb^/Grm7^+^ | 129P2/OlaHsd * FVB/N | ANSM |
| Smn1^tm1Msd^/Smn1^tm1Msd^ Tg(SMN1-SMN2*)16Cll/0 Grm7^Tg(SMN2)89Ahmb^/Grm7^Tg(SMN2)89Ahmb^ | 129P2/OlaHsd * C57BL/6 * FVB/N | ANSM |
| Smn1^tm1Msd^/Smn1^tm1Msd^ Tg(SMN2)11Tro/0 Tg(SMN2)46Tro/0 | B6.Cg-Tg(SMN2)11Tro Tg(SMN2)46Tro Smn1^tm1Msd^/J | ANSM |
| Smn1^tm5(Smn1/SMN2)Mrph^/Smn1^tm5(Smn1/SMN2)Mrph^ | 129S6/SvEvTac * C57BL/6J * C57BL/6NTac | ANSM AEP AMEP |
| Snap25^tm1Mcw^/Snap25^tm1Mcw^ | C57BL/6 | AEP |
| Snta1^tm1Scf^/Snta1^tm1Scf^ | 129P2/OlaHsd * C57BL/6 | ANSM |
| Snta1^tm1Scf^/Snta1^tm1Scf^ Sntb2^tm1Scf^/Sntb2^tm1Scf^ | 129P2/OlaHsd * C57BL/6 | ANSM AMEP |
| Stac3^tm1a(KOMP)Wtsi^/Stac3^tm1a(KOMP)Wtsi^ | C57BL/6N | ANSM AMEP |
| Stxbp5l^tm1.1Aljg^/Stxbp5l^tm1.1Aljg^ | C57BL/6 | AEP |
| Syne1^tm1Rexu^/Syne1^tm1Rexu^ | 129 * C57BL/6 | ANSM |
| Syne1^tm1Rexu^/Syne1^tm1Rexu^ Syne2^tm1Rexu^/Syne2^+^ | 129 * C57BL/6 | ANSM |
| Syne1^tm1Rexu^/Syne1^tm1Rexu^ Syne2^tm1Rexu^/Syne2^tm1Rexu^ | 129 * C57BL/6 | ANSM |
| Syt2^m1Ingm^/Syt2^m1Ingm^ | Not Specified | AMEP |
| Syt2^tm1Sud^/Syt2^tm1Sud^ | B6.129-Syt2^tm1Sud^ | AMEP |
| Tardbp^tm1.1Sobue^/Tardbp^tm1.1Sobue^ Tg(SLC18A3-cre)Misa/0 | 129S6/SvEvTac * C57BL/6 * C57BL/6J * C57BL/6NTac | ANSM |
| Tardbp^tm3.1Ckjs^/Tardbp^+^ | C57BL/6J | ANSM |
| Tg(C9orf72)500Lpwr/0 | FVB/NJ-Tg(C9orf72)500Lpwr | ANSM |
| Tg(Ckm-Chrnd*S262T)40Cgz/0 | C57BL/6 * DBA/2 | AEP AMEP |
| Tg(Ckm-Chrne*L269F)5Cgz/? | FVB/NJ | ANSM AEP AMEP |
| Tg(Ckm-Syne2)1Rexu/0 | FVB | ANSM |
| Tg(DMWD,DMPK*,SIX5)328Ggo/ Tg(DMWD,DMPK*,SIX5)328Ggo | C57BL/6 * DBA/2 | ANSM |
| Tg(NEFH-tTA)8Vle/0 Tg(tetO-TARDBP*)4Vle/0 | C3H/HeJ * C57BL/6J | ANSM |
| Tg(Prnp-FUS)WT3Cshw/ Tg(Prnp-FUS)WT3Cshw | C57BL/6 * Crl:CD-1(ICR) | ANSM |
| Tg(Prnp-FUS*R521C)3313Ejh/0 | C57BL/6 * SJL | ANSM |

**Table S1.** (*continued*)

| **Allele composition** | **Genetic background** | **Annotated MGI term** |
| --- | --- | --- |
| Tg(Prnp-TARDBP)96Dwc/0 Tg(Prnp-TARDBP*Q331K)31Dwc/0 | C3H * C57BL/6 | ANSM |
| Tg(Prnp-TARDBP*Q331K)31Dwc/? | C3H * C57BL/6 | ANSM |
| Tg(Prnp-TARDBP*Q331K)103Dwc/? | C57BL/6 * C3H | ANSM |
| Tg(TARDBP)#Jpj/0 | C3H * C57BL/6 | ANSM |
| Tg(TARDBP*G348C)#Jpj/0 | C3H * C57BL/6 | ANSM |
| Tg(tetO-NEFL)173.2Jpj/0 Tg(THY1-tTA)177Jpj/0 | C3H * C57BL/6 | ANSM |
| Tg(Thy1-DCTN1*G59S)M2Pcw/? | C57BL/6 * SJL | ANSM |
| Tg(Thy1-HSPB1*P182L)#Lvdb/0 | FVB/N | ANSM |
| Tg(Thy1-HSPB1*S135F)#Lvdb/0 | FVB/N | ANSM |
| Tg(Thy1-Snca)1S13Putt/? | C57BL/6 | ANSM |
| Tg(Thy1-SNCA*A53T)9813Putt/0 | C57BL/6 | ANSM |
| Tg(Thy1-SOD1*G93A)T1Hgrd/0 Tg(Thy1-SOD1*G93A)T3Hgrd/0 | C57BL/6 * CBA * FVB | ANSM |
| Tg(Thy1-SOD1*G93A)T3Hgrd/ Tg(Thy1-SOD1*G93A)T3Hgrd | C57BL/6 * CBA * FVB | ANSM |
| Tmem106b^em1Damme^/Tmem106b^em1Damme^ | C57BL/6-Tmem106b^em1Damme^ | ANSM |
| Tmem184b^Gt(IST10294F4)Tigm^/ Tmem184b^Gt(IST10294F4)Tigm^ | C57BL/6N | ANSM |
| Uchl1^tm1Dgen^/Uchl1^tm1Dgen^ | B6.129P2-Uchl1^tm1Dgen^/Mmnc | ANSM AEP AMEP |
| Usp14^ax-J^/Usp14^ax-J^ | B6.Cg-Usp14^ax-J^ | ANSM AMEP |
| Usp14^nmf375^/Usp14^nmf375^ | C.B6-Usp14^nmf375^ | ANSM |
| Utp14b^jsd^/Utp14b^jsd^ | C57BL/6J | FNSPoD |
| Utrn^tm1Jrs^/Utrn^tm1Jrs^ | 129S1/Sv * 129X1/SvJ | ANSM |
| Utrn^tm1Ked^/Utrn^tm1Ked^ | 129S1/Sv * 129X1/SvJ * C57BL/6 * DBA | ANSM AMEP |
| Vapb^tm1.1Tsud^/Vapb^+^ | 129S7/SvEvBrd | ANSM |
| Vapb^tm1.1Tsud^/Vapb^tm1.1Tsud^ | 129S7/SvEvBrd | ANSM |

**Table S2**. Unique genes involved in the mouse models associated with NMJ-related abnormalities registered at the Mouse Genome Informatics MGI database (September, 2022). Associated neuromuscular diseases, as described at www.musclegenetable.fr, are indicated with a code (disease group and disease separated by a dot), the name of the disease and the type of inheritance. Note that congenital myasthenic syndromes are clustered in group 11 and associated genes are marked with an asterisk *. AD: Autosomal Dominant; AR: Autosomal Recessive; N/A: Not Applicable; XR: X-linked.

| **Gene** | **Neuromuscular disease (Code: Disease – (Inheritance))** |
| --- | --- |
| *Adarb1/Adar2* | N/A |
| *Afg3l2* | 13.25: Spinocerebellar ataxia 28 - (AD)  15.80: Spastic ataxia 5 autosomal recessive - (AR) |
| *Agrn** | 11.16: Myasthenic syndrome, congenital, 8, with pre- and postsynaptic defects - (AR)  16.31: Fetal akinesia deformation related to AGRN- (AR) |
| *Als2* | 12.45: Amyotrophic lateral sclerosis 2 juvenile - (AR)  15.69: Infantile-onset ascending spastic paraplegia- (AR) |
| *Aplp2* | N/A |
| *App* | N/A |
| *Atp7a* | 12.42: Spinal muscular atrophy, distal, X-linked, 3 - (XR) |
| *Basp1/Cap23* | N/A |
| *Borcs5* | N/A |
| *C9orf72* | 12.72: Amyotrophic lateral sclerosis and/or frontotemporal dementia - (AD) |
| *Cacna1a* | 7.9: Acetazolamide responsive hereditary paroxysmal cerebellar ataxia - (AD)  7.10: Episodic ataxia type-2 - (AD)  13.6: Spinocerebellar ataxia 6 - (AD)  13.45: Episodic ataxia type-2 ,and familial hemiplegic migraine - (AD)  13.50: Acetazolamide responsive hereditary paroxysmal cerebellar ataxia - (AD) |
| *Cacna2d2* | N/A |
| *Cacnb4* | 13.47: Episodic ataxia type-5 - (AD) |
| *Cacng2* | N/A |
| *ChAT** | 11.13: Myasthenic syndrome, congenital, 6, presynaptic - (AR) |
| *Chchd10* | 12.36: Spinal muscular atrophy, Jokela type - (AD)  12.73: Amyotrophic lateral sclerosis and/or frontotemporal dementia - (AD)  16.62: Isolated mitochondrial myopathy - (AD) |
| *Chmp2b* | 12.60: Amyotrophic lateral sclerosis 17 - (AD) |
| *Chrna1** | 11.1: Myasthenic syndrome, congenital, 1A, Slow-channel - (AD)  11.5: Myasthenic syndrome, congenital, 1B, Fast-channel - (AR) |
| *Chrna7* | N/A |
| *Chrnb1** | 11.2: Myasthenic syndrome, congenital, 2A, Slow-channel - (AD)  11.6: Myasthenic syndrome, congenital, 2B, Fast-channel - (AR)  11.9: Myasthenic syndrome, congenital, 2C, associated with acetylcholine receptor deficiency - (AR) |
| *Chrnd** | 11.3: Myasthenic syndrome, congenital, 3A, Slow-channel - (AD)  11.7: Myasthenic syndrome, congenital, 3B, Fast-channel - (AR  11.10: Myasthenic syndrome, congenital, 3C, associated with acetylcholine receptor deficiency - (AR) |

**Table S2.** (*continued*)

| **Gene** | **Neuromuscular disease (Code: Disease – (Inheritance))** |
| --- | --- |
| *Chrne** | 11.4: Myasthenic syndrome, congenital, 4A, Slow-channel - (AD, AR)  11.8: Myasthenic syndrome, congenital, 4B, Fast-channel - (AR)  11.11: Myasthenic syndrome, congenital, 4C, associated with acetylcholine receptor deficiency - (AR) |
| *Chrng** | 11.35: Escobar syndrome (multiple pterygium syndrome) - (AR) |
| *Clip3/CLIPR-59* | N/A |
| *Clp1* | N/A |
| *Col13a1** | 11.27: Congenital myasthenic syndrome type 19 - (AR) |
| *Colq** | 11.12: Myasthenic syndrome, congenital, 5 - (AR) |
| *Cpeb4* | N/A |
| *Csnk2b* | N/A |
| *Dag1* | 1.45: Limb-Girdle Muscular Dystrophy type R16 - (AR)  2.38: Congenital muscular dystrophy with hypoglycosylation of dystroglycan type A9 - (AR) |
| *Dctn1* | 12.71: Amyotrophic lateral sclerosis related to dynactin 1, susceptibility to - (AD) |
| *Dhtkd1* | 14.61: Charcot-Marie-Tooth neuropathy Type 2Q - (AD) |
| *Dmd* | 1.1: Duchenne/Becker Muscular Dystrophies - (XR)  10.81: Dilated cardiomyopathy, 3B - (XR) |
| *Dmpk* | 6.1: Myotonic dystrophy 1 (Steinert) - (AD) |
| *Dnajc5/Csp-alpha* | N/A |
| *Dnm2* | 2.15: Congenital muscular dystrophy related to DNM2 - (AD)  3.23: Centronuclear myopathy 1 - (AD)  4.16: Distal myopathy related to DNM2 - (AD)  12.82: Lethal Congenital Contracture Syndrome 5 - (AR)  14.14: Charcot-Marie-Tooth disease, dominant intermediate B - (AD) |
| *Dnmt3a* | N/A |
| *Dok7** | 11.18: Myasthenic syndrome, congenital, 10 - (AR)  16.27: Fetal akinesia deformation sequence 3 - (AR) |
| *Dst* | 14.106: Hereditary sensory and autonomic neuropathy type VI - (AR) |
| *Dtna* | 10.92: Left ventricular noncompaction, 1 - (AD) |
| *Dync1h1* | 12.32: Spinal muscular atrophy, lower extremity, autosomal dominant - (AD)  14.59: Charcot-Marie-Tooth neuropathy Type 2O - (AD) |
| *Ecel1* | 16.18: Arthrogryposis, distal, type 5D - (AR) |
| *Eef1a2* | N/A |
| *Egr2* | 14.4: Charcot-Marie-Tooth disease, type 1D - (AD)  14.27: Neuropathy, congenital hypomyelinating, 1- ( AR)  14.44: Dejerine-Sottas hypertrophic neuropathy, dominant - (AD digenic) |
| *Erbb2* | N/A |
| *Erbb4* | 12.62: Amyotrophic lateral sclerosis 19 - (AD) |
| *Fat1* | N/A |
| *Fbxo45* | N/A |

**Table S2.** (*continued*)

| **Gene** | **Neuromuscular disease (Code: Disease – (Inheritance))** |
| --- | --- |
| *Fgfbp1* | N/A |
| *Fus* | 12.49: Amyotrophic lateral sclerosis 6, with or without frontotemporal dementia - (AD) |
| *Gabpa* | N/A |
| *Gars* | 12.21: Distal spinal muscular atrophy, distal with upper limb predominance (type V)  - (AD)  14.50: Charcot-Marie-Tooth neuropathy Type 2D - (AD) |
| *Gas7* | N/A |
| *Gdap1* | 14.21: Charcot-Marie-Tooth disease Type 4A - (AR)  14.56: Charcot-Marie-Tooth neuropathy Type 2K - (AD, AR)  14.82: Charcot-Marie-Tooth disease, recessive intermediate, A - (AR) |
| *Gfpt1** | 11.20: Myasthenia, congenital, 12, with tubular aggregates - (AR) |
| *Gphn/Geph* | N/A |
| *Hspb1* | 12.18: Neuropathy, distal hereditary motor, type IIB - (AD)  14.52: Charcot-Marie-Tooth neuropathy Type 2F - (AD, AR) |
| *Hspg2* | 6.8: Schwartz-Jampel syndrome - (AR) |
| *Ighmbp2* | 12.5: Spinal muscular atrophy, distal autosomal recessive 1 ( with respiratory distress) - (AR)  14.87: Charcot-Marie-Tooth disease, type 2S - (AR) |
| *Itgb1* | N/A |
| *Kalrn* | N/A |
| *Ky* | 5.8: Myofibrillar myopathy 7 - (AR) |
| *Lama4* | 10.71: Dilated cardiomyopathy, 1JJ - (AD) |
| *Lama5** | 11.38: Presynaptic congenital myasthenic syndrome - (AR) |
| *Lamb2** | 11.34: Congenital myasthenic syndrome with nephrotic syndrome - (AR) |
| *Large1* | 2.34: Congenital muscular dystrophy with hypoglycosylation of dystroglycan - (AR) |
| *Lgals1/Galectin-1* | N/A |
| *Lrp4** | 11.25: Myasthenic syndrome, congenital, 17 - (AR) |
| *Lrsam1* | 14.60: Charcot-Marie-Tooth neuropathy Type 2P - (AD) |
| *Madd/Rab3 GEP* | N/A |
| *Mapt* | 12.99: Lower motor neuron disease with respiratory failure related to MAPT - (AD) |
| *Matr3* | 4.5: Vocal cord and pharyngeal distal myopathy (VCPDM) reclassified as ALS21   - (AD)  12.64: Amyotrophic lateral sclerosis 21 - (AD) |
| *Mbnl1* | N/A |
| *Mbnl2* | N/A |
| *Met* | 16.23: Arthrogryposis and muscular dysplasia - (AD) |
| *miR-1/206/133* | N/A |
| *miR-218-1/2* | N/A |

**Table S2.** (*continued*)

| **Gene** | **Neuromuscular disease (Code: Disease – (Inheritance))** |
| --- | --- |
| *Mmp3/ Stromelysin-1* | N/A |
| *Mnx1/Hb9* | N/A |
| *MuSK** | 11.17: Myasthenic syndrome, congenital, 9, associated with acetylcholine receptor deficiency - (AR)  16.26: Fetal akinesia deformation sequence 1 - (AR) |
| *Nbea* | N/A |
| *Nefl* | 14.7: Charcot-Marie-Tooth neuropathy Type 1F - (AD)  14.19: Charcot-Marie-Tooth disease, dominant intermediate G - (AD)  14.51: Charcot-Marie-Tooth neuropathy Type 2E - (AD) |
| *Nemf* | N/A |
| *Nes* | N/A |
| *Nmf67* | N/A |
| *Nrcam* | N/A |
| *Nrg1/ARIA* | N/A |
| *Pex10* | N/A |
| *Pfn1* | 12.61: Amyotrophic lateral sclerosis 18 - (AD) |
| *Phr1/Mycbp2* | N/A |
| *Plaa* | N/A |
| *Pls3* | N/A |
| *Prkcq/PKCθ* | N/A |
| *Psmf1/PI31* | N/A |
| *Rab18* | N/A |
| *Rapsn** | 11.19: Myasthenic syndrome, congenital, 11, associated with acetylcholine receptor deficiency - (AR)  16.28: Fetal akinesia deformation sequence 2 - (AR) |
| *Rer1* | N/A |
| *Ret* | N/A |
| *Rims1* | N/A |
| *Rims2* | N/A |
| *Rnd3/RhoE* | N/A |
| *Scn8a/Nav 1.6* | N/A |
| *Scyl1* | 13.76: Spinocerebellar ataxia, autosomal recessive 21 - (AR) |
| *Setx* | 12.47: Amyotrophic lateral sclerosis 4 - (AD)  13.87: Spinocerebellar ataxia with axonal neuropathy type 2 - (AR) |
| *Sh3tc2* | 14.25: Charcot-Marie-Tooth disease, Type 4C - (AR) |
| *Skor2* | N/A |
| *Slc16a3/Mct4* | N/A |
| *Slc18a3** | 11.29: Myasthenic syndrome, congenital, 21, presynaptic - (AR) |

**Table S2.** (*continued*)

| **Gene** | **Neuromuscular disease (Code: Disease – (Inheritance))** |
| --- | --- |
| *Slc25a46* | 12.92: Pontocerebellar hypoplasia with spinal muscular atrophy - (AR) |
| *Slc5a7** | 11.28: Myasthenic syndrome, congenital, 20, presynaptic - (AR)  12.26: Spinal muscular atrophy, distal, with vocal cord paralysis (Harper-Young) - (AD) |
| *Slc6a5/Glyt2* | N/A |
| *Smn1* | 12.1: Spinal muscular atrophy , type I (Werdnig-Hoffman ) - (AR)  12.2: Spinal muscular atrophy, type II (intermediate) - (AR)  12.3: Spinal muscular atrophy, type III (Kugelberg-Welander) - (AR)  12.4: Spinal muscular atrophy, type IV, adult form - (AR) |
| *Snap25** | 11.26: Myasthenic syndrome, congenital, 18 - (AD) |
| *Snca* | N/A |
| *Snta1* | 10.135: Long QT syndrome 12 - (AD) |
| *Sntb1* | N/A |
| *Sntb2* | N/A |
| *Sod1* | 12.43: Amyotrophic lateral sclerosis 1 (dominant) - (AD)  12.44: Amyotrophic lateral sclerosis 1 (recessive) - (AR) |
| *Spin1* | N/A |
| *Stac3* | 3.54: Myopathy, congenital, Baily-Bloch (Native American myopathy) - (AR)  3.55: Myopathy, congenital, with malignant hyperthermia susceptibility - (AR) |
| *Stxbp5l/Tom2* | N/A |
| *Syne1* | 1.6: Emery-Dreifuss muscular dystrophy 4, autosomal dominant - (AD)  10.82: Dilated cardiomyopathy related to nesprin-1- (AD)  13.63: Autosomal recessive spinocerebellar ataxia, 8 - (AR)  16.21: Arthrogryposis multiplex congenita with nesprin-1 defect - (AR) |
| *Syne2* | 1.7: Emery-Dreifuss muscular dystrophy 5, autosomal dominant - (AD) |
| *Syt2** | 11.14: Myasthenic syndrome, congenital, 7, presynaptic - (AD)  11.15: Myasthenic syndrome, congenital, 7B, presynaptic - (AR)  12.37: Distal motor neuropathy related to SYT2 - (AD) |
| *Tardbp* | 12.53: Amyotrophic lateral sclerosis 10, with or without frontotemporal dementia - (AD) |
| *Tmem106b* | N/A |
| *Tmem184b* | N/A |
| *Uchl1* | 15.67: Spastic paraplegia 79, autosomal recessive - (AR) |
| *Usp14* | N/A |
| *Utp14b* | N/A |
| *Utrn* | N/A |
| *Vapb* | 12.35: Spinal muscular atrophy, late-onset, Finkel type - (AD)  12.51: Amyotrophic lateral sclerosis 8 - (AD) |

**Table S3**. Novel candidate genes for NMJ-related diseases in humans based on phenotypes from mouse models registered at the Mouse Genome Informatics MGI database. Extended. All models are constitutive unless otherwise stated. Lethal phenotypes can have complete or incomplete penetrance. ACh: acetylcholine; AChR: acetylcholine receptor; ALS: Amyotrophic lateral sclerosis; cKO: constitutive KO; CT: connective tissue; DGC: dystrophin-associated glycoprotein complex; DM: Myotonic dystrophy; FSHD: Facioscapulohumeral muscular dystrophy; MN: motor neuron; NMJ: neuromuscular junction; PM: point-mutation; SC: Schwann cell; Skm: skeletal muscle; WT: Wild-type.

| **Gene** | **Model** | **Phenotype** | **Lethality^1^** |
| --- | --- | --- | --- |
| **Presynaptic terminal** | | | |
| MN identity, function and survival | | | |
| *Adarb1/Adar2* | MN-specific KO (*VAChT-Cre*) | Degeneration of MN axons resulting in denervation of NMJs | PD |
| *Clp1* | Kinase-dead isoform | MN axonal degeneration leading to MN apoptosis and subsequent denervation of NMJs | SD: V, NL or PD |
| *Gphn/Geph* | cKO | Increased MN survival, increased number of NMJs and axon branching capacity, and reduced motor activity in limbs, where glycine has an inhibitory role. The opposite effects are observed in the diaphragm, where this neurotransmitter is excitatory. No changes in NMJ morphology or in the postsynaptic terminal in either case | SD: V or NL |
| *Mnx1/Hb9* | cKO | Defective innervation of the diaphragm -but not of limb muscles- due to misrouting of axons. In this muscle, which is thinner than normal, AChR clusters are dispersed, abnormally small and not correctly apposed to the presynaptic terminal because the lack of nerve-derived agrin in the absence of MNs, interferes with postsynaptic differentiation | PNL |
| *Pex10* | Chemically induced PM affecting protein function | SCs are reduced in number, abnormally shaped and not properly placed in the NMJ, causing a progressive loss of MN axon integrity. Reduced apposition of pre- and postsynaptic terminals, with many axons extending beyond the endplate. Some axons lack synaptic vesicles, while postsynaptic terminals are normal. Diminished synaptic transmission during development | PNL |
| *Ret* | cKO and cranial MN-specific KO (*Nkx6.2-Cre*) | NMJs in cKOs are less developed, as exemplified by presynaptic nerve sprouting and impaired postsynaptic AChR clustering. MN-specific KOs have a reduced number of NMJs but they are mostly morphologically normal | PNL (cKO) |
| *Slc6a5/Glyt2* | cKO by spontaneous retrotransposon insertion | Accelerated synapse elimination to achieve fiber monoinnervation, as well as accelerated switch from embryonic to adult AChR subunit types, concomitant with an increase in AChR transcription in the postsynaptic cell. There is a trend towards smaller but normally shaped NMJs | PL |
|  |  |  |  |
| Channel-related | | | |
| *Cacna2d2* | Spontaneous mutation leading to a shorter transcript (*Ducky* mice) | Small reduction of evoked ACh release, but rather than a consequence of reduced voltage-activated Ca^2+^ channel function, it could be attributable to the smaller size of ducky mice, which present reduced NMJ size as well as muscle fiber diameter | SD: V or PM |
| *Cacng2* | Hypomorph by spontaneous transposon insertion (*Stargazer* mice) | Smaller NMJ size | - |

**Table S3.** (*continued*)

| **Gene** | **Model** | **Phenotype** | **Lethality^1^** |
| --- | --- | --- | --- |
| Regulation of gene expression at the transcript and protein levels. Proteostasis and autophagy | | | |
| *Borcs5* | cKO | Neuroaxonal dystrophy. Normal NMJ morphology | PNL |
| *Dnmt3a* | Nervous system-specific KO (*Nes-Cre*) | Normal looking MNs in the spinal cord, with normal innervation, but high NMJ fragmentation. No signs of skeletal muscle degeneration | PD |
| *Eef1a2* | cKO by spontaneous PM (*Wasted* mice) | Aggressive, recessive and early-onset form of neurodegeneration characterized by reactive gliosis in spinal cord, followed by progressive retraction of MN axons from muscle and MN death. Extensive muscle wasting that cannot be solely explained by denervation, but overexpression of *Eef1a2* in muscle does not ameliorate the phenotype | PD |
| *miR-218-1/2* | cKO | Normal MN fate specification and development, but axons fail to form proper NMJs at the target muscles, as exemplified by reduced terminal branching and the lack of innervation of the majority of AChR clusters. Progressive MN loss is also observed | NL |
| *Nemf* | cKO and chemically induced PM affecting protein function | The PM models show denervation and fragmentation of the postsynaptic terminals, progressive axonal degeneration, and specific abnormalities in skeletal muscle that depend on the model. cKO mice show more severe phenotypes | AD: V or PD |
| *Plaa* | cKO and G23V PM recapitulating human PLAA-associated neurodevelopmental disorder | Homozygous mice for the G23V point mutation show swelling and/or sprouting of axon terminals, a reduced number of synaptic vesicles and the presence of enlarged endosomes and vacuoles, as well as muscle atrophy. These defects lead to altered synaptic transmission. Compound G23V/- mutants present a more severe phenotype while homozygous KOs die before birth | AD: PM or PNL |
| *Psmf1/PI31* | MN-specific KO (*Mnx1-Cre2*) | Presynaptic terminals appear fragmented, with accumulation of polyubiquitinated proteins and massive axonal sprouting, while muscles get atrophied. Phenotypes get progressively more severe with age | -  (EL cKO) |
| *Rab18* | cKO | Disorganization of the cytoskeleton in presynaptic terminals, with randomly oriented filaments. No abnormalities in myelination, endplate area or in the morphology of postsynaptic terminals, although progressive muscle weakness is observed. No dying-back pathology. In essence, no hallmark features of neurodegeneration | PNL |
| *Tmem106b* | cKO | MN axons are slightly but significantly shorter, and contain vacuoles. There is a high percentage of partially or fully denervated NMJs | - |
| *Tmem184b* | cKO | Presynaptic terminals present large dystrophic swellings and an abnormal accumulation of autophagosomal structures. Postsynaptic AChRs are virtually absent in areas of presynaptic swelling, thus causing reduced apposition of the pre- and postsynaptic terminals. Muscle fibers occasionally accumulate autophagosomes/lysosomes as well. Neuroprotection is illustrated by a short but significant delay in axon degeneration after injury | - |

**Table S3.** (*continued*)

| **Gene** | **Model** | | **Phenotype** | **Lethality^1^** |
| --- | --- | --- | --- | --- |
| *Usp14* | Hypomorphs by chemically induced PM (nmf375 mice) and by spontaneous PM (Ataxia mice) | | Specific phenotypes depend on the mouse strain and mutant allele. nmf375 mice show progressive defects in NMJs with a significant upregulation of AChRs subunits, altered synaptic transmission, abnormal accumulation of neurofilaments at presynaptic terminals and a reduction in the levels of monomeric ubiquitin. axJ mice exhibit more severe phenotypes, especially at the presynaptic terminals, with large neurofilament accumulations, terminal swelling and sprouting, poor terminal arborization, NMJ denervation, and reduced ACh release, concomitant with defective synaptic transmission. Additionally, muscle size is reduced and the distribution of AChRs reflects an immature stage | SD: V, PNL or PD |
|  | |  |  |  |
| Cytoskeleton dynamics | |  |  |  |
| *Basp1/Cap23* | cKO | | Defect in stimulus-induced nerve sprouting, expanded synaptic regions, and presence of tSC processes | PL |
| *Clip3/ CLIPR-59* | cKO | | Lower density of nerve terminals and reduced number of terminals capped by tSCs, leading to a defect in MN axon stability during late embryogenesis | PNL |
| *Cpeb4* | Dominant negative truncated protein | | Defect in neurite outgrowth and consequently, in NMJ formation | NL |
| *Fbxo45* | cKO | | Incomplete innervation of the diaphragm, excessive branching of axon terminals, sometimes extending across the endplate bands, which are narrower than in WT conditions | NL |
|  |  | |  |  |
| *Phr1/Mycbp2* | cKO, MN-specific deletion (*Mnx1-Cre*^2^), C-terminal truncated protein (*Magellan* mice), mutant lacking exons 8-9, and PM affecting protein function | | MN axon terminals show excessive sprouting and varicose endings, even extending extrasynaptically, due to defective growth cones that cause navigation errors. These defects lead to fewer axons reaching their targets in muscle and ultimately, to impaired synaptic transmission. No signs of increased MN death or axon degeneration. Depending on the model, either diffuse AChR clustering or densely packed and narrow endplate bands are found at the postsynaptic terminal | AD: V, PNL or NL |
| *Rnd3/RhoE* | cKO | | Less number of MNs, immature NMJs and absence of the peroneal nerve, which results in the atrophy of the target muscles. The rest of the muscles are smaller, without signs of degeneration/regeneration | PD |

**Table S3.** (*continued*)

| **Gene** | **Model** | | **Phenotype** | | | **Lethality^1^** | |
| --- | --- | --- | --- | --- | --- | --- | --- |
| Defective signaling among tSCs, pre-, and post-synaptic terminals. Defective synaptic transmission | | | | | | |  |
| *Nrcam* | | cKO by spontaneous B2 insertion; compound *Nrcam/Gars* and *Nrcam/Sh3tc2* mutants | | Single *Nrcam* mutants do not show an overt neuromuscular phenotype, similarly to *Sh3tc2* single mutants. Double KOs show the typical demyelinating effects of *Sh3tc2* mutants as well as NMJ fragmentation, nerve terminal sprouting, extrasynaptic AchR expression and defective action potential conduction at the presynaptic terminal. Similar phenotypes in double *Gars^+/-^Nrcam^-/-^* mutants, with additional muscle atrophy due to *Gars* happloinsufficiency | PD (*Nrcam/ Sh3tc2* KOs) | |  |
| *Scn8a/Nav1.6* | | cKO by spontaneous LINE1 insertion (*Med* mice); cKO by spontaneous PM (*m10J* mice); compound *Scn8a/Gars* mutants | | In *Med* KO mice, axons appear thick, swollen, with reduced branching and high content of vesicles, presumably due to impaired synaptic transmission. Reduction in the number of tSCs is the first observed cellular defect. Delayed maturation of NMJs: they are smaller and abnormally shaped. Increased presence of multi-innervated fibers. No denervation and close-to-normal apposition of pre- and postsynaptic terminals are observed. The NMJ morphological defects and the reduction in nerve conduction velocity characteristic of *Gars* mutants are exacerbated in the case of *Scn8a* happloinsuffiency | SD and AD: V or PL | |  |
|  | |  | |  |  | |  |
| Endocytosis, synaptic vesicle formation and neurotransmitter release | | | | | | |  |
| *Dnajc5/Csp-⍺* | | cKO | | Immature postsynaptic terminals, reduced apposition of pre and post-synaptic terminals, vacuolization of presynaptic terminals and presence of multilamellar bodies, invagination of presynaptic terminals by tSCs and impairment of synaptic transmission | PD | |  |
| *Madd/Rab3 GEP* | | cKO | | Normal nerve conduction at the presynaptic component, but 10-fold reduction in vesicle content, with many of the few existing vesicles being large, empty and/or mislocalized, causing impaired neuromuscular transmission. Axon terminals are enlarged and contain degenerated mitochondria. Normal NMJs and postsynaptic specializations | SD: V or NL | |  |
| *Nbea* | | cKO by random integration that in addition disturbs GH secretion causing dwarfism; cKO by gene trap | | Block of synaptic transmission, albeit with normal action potential conduction along MNs and normal muscle function. NMJ morphology appears unaltered | NL | |  |
| *Pls3* | | Overexpression in SMA models (*Smn1^-/-^/Tg(SMN2*)) | | In mild SMA mouse models, *Pls3* overexpression stabilizes axons, and restores synaptic vesicle recycling and release processes at the presynaptic terminal. At the postsynaptic level, endplate and muscle fiber sizes are increased. All in all, NMJ functionality, motor function and animal survival are moderately ameliorated in these mice. In contrast, in severe SMA models only mild protection effects are observed, and they are insufficient to improve survival or motor function | PD | |  |

**Table S3.** (*continued*)

| **Gene** | **Model** | **Phenotype** | | **Lethality^1^** |
| --- | --- | --- | --- | --- |
| *Rims1* and *Rims2* | cKO | NMJs are mostly morphologically normal, although there is extensive nerve terminal branching that covers a broader than usual area in the diaphragm, possibly in response to the impaired synaptic transmission observed in the double mutants, which die immediately after birth. In contrast, single mutants are viable and thus, some functional compensation between paralogs presumably takes place, although there are no compensatory changes at protein level in either single model. Single *Rims1^-/-^*, but not single *Rims2^-/-^* mice, show impaired synaptic transmission as well | | NL |
| *Stxbp5l/Tom2* | cKO | No morphological defects in MNs, NMJs or muscle, but altered synaptic transmission: enhanced spontaneous ACh release and increased synaptic fatigue upon sustained stimulation | | SD: V or PL |
|  |  |  | |  |
| Unknown | | | | |
| *Slc16a3/Mct4* | cKO | No impact in MN survival but axon diameter and length are increased in the *Mct4* mutants. Muscle-specific progressive degeneration of the NMJs is observed, with progressive fragmentation in the postsynaptic terminals. These defects are accompanied by abnormal synaptic transmission. Muscles have normal morphology and contractility even if they accumulate high amounts of intramuscular lactate | | - |
| *Snca* | Constitutive overexpression of mouse WT or human PD-associated A53T mutant form | MN axons degenerate and retract from NMJs, thus leading to muscle denervation in both models, with mice overexpressing the human mutant A56T SNCA protein showing a more severe phenotype. In the case of the mouse WT Snca overexpression model, muscles show some signs of atrophy, and motor impairment appears later and in a much less pronounced manner than in the case of human WT SNCA overexpression. Pronounced ubiquitin immunopathology is also observed in the spinal cord | | AD: V or PD |
|  |  |  | |  |
| **Synaptic cleft** | | |  |  |
| Channel-related | | | | |
| *Mmp3* | cKO | Extensive remodeling of the postsynaptic membrane: increased density of AChR clusters, bigger endplate areas, increased number and size of junctional folds, as well as ectopic junctional folds. All these morphological changes have little electrophysiological impact | | - |
|  |  |  | |  |
| Defective signaling among tSCs, pre-, and post-synaptic terminals. Defective synaptic transmission | | | | |
| *Fgfbp1* | cKO | Increased fragmentation of NMJs, suggesting accelerated ageing, but no signs of denervation. Increased expression of some AChR subunits as well as of atrogenes in the muscle terminal | | - |

**Table S3.** (*continued*)

| **Gene** | **Model** | **Phenotype** | **Lethality^1^** |
| --- | --- | --- | --- |
| Adhesion | | | |
| *Aplp2* and *App* | Skeletal muscle- (*Ckmm-Cre*), nervous system- (*Nes-Cre*), neuron-specific (*ChAT-Cre*) or constitutive *App* deletion in a *Aplp2* cKO background; humanized mutated and truncated *App* transgene in a *Aplp2* cKO background; constitutive deletion of the *Lrp4* transmembrane domain in an *App* cKO background | In double *App/Aplp2* KOs, NMJs show a diffuse patterning of the synaptic terminals with reduced apposition between them. There is excessive sprouting of the presynaptic terminal, which in addition contains a reduced number of synaptic vesicles. All these phenotypes are concomitant with defects in synaptic transmission | AD: V, PNL, PL or PD |
| *Itgb1* | Neural crest- (*PLAT-Cre*), nervous system- (*Nes-Cre*) or skeletal muscle-specific (*ACTA1-Cre*) KOs | In tSCs-specific *Itgb1* mutants, there is a nearly complete absence of tSCs at the presynaptic terminals, and defects in axon arborization and morphology are observed. NMJs appear immature, with abnormal ECM composition and organization. Failure of many axons to establish functional NMJs. Abnormal density of AChR clusters at the postsynaptic terminal, and progressive muscle atrophy. When *Itgb1* is absent from muscles, presynaptic terminal morphology is altered, synaptic contacts are not correctly established, muscles are smaller and mice die at birth | PD |
| *Fat1* | Skeletal muscle-specific (*Pax3-Cre*) hypomorph; skeletal muscle- (*Pax3-Cre*), MN- (*Olig2-Cre*), mesenchymal- except cranial; (*Prx1-Cre*), craniofacial mesenchymal-specific (*Wnt1-Cre*) or constitutive mutant lacking the transmembrane domain | Altered coupling of muscular and neuronal morphogenesis. In all constitutive, MN-, CT- or Skm-specific *Fat1* mutants, NMJs deteriorate and the migration of myogenic progenitors is compromised, albeit at various extents. Muscle defects are more severe in Skm-specific *Fat1* mice, which present abnormal shape and position of specific muscle groups, in particular, of those associated with FSHD. These mutant muscles exhibit misoriented fibers, necrosis, and reduced fiber size | AD: V, NL or PD |

**Table S3.** (*continued*)

| **Gene** | **Model** | | **Phenotype** | **Lethality^1^** |
| --- | --- | --- | --- | --- |
| **Postsynaptic terminal** | |  |  |  |
| Channel-related | |  |  |  |
| *Csnk2b* | Skeletal muscle-specific KO (*ACTA1-Cre*) | | Fragmentation and disintegration of the AChR clusters at the postsynaptic terminal, disrupting the typical pretzel-like shape. Impairment of synaptic transmission by affecting sustained neurotransmitter release and reducing post-synaptic sensitivity to Ach | - |
| *Nes* | cKO | | Increased number of AChR clusters, nerve length and endplate area. Reduced skeletal muscle mass due to frequent spontaneous regeneration. | - |
| *Prkcq/PKCθ* | cKO | | Delayed activity-dependent synapse elimination process to achieve monoinnervation of NMJs during the early postnatal period by about 4 days. Delayed maturation of the presynaptic terminal. Beyond this point, NMJ morphology and function were normal. No other abnormalities | - |
| *Rer1* | Heterozygous cKO | | Smaller normally-shaped NMJ size that precedes a reduction in muscle fiber size. The amount of fully assembled AChR receptors is reduced | -  (EL cKO) |
|  | | | |  |
| Regulation of gene expression at the transcript and protein levels. Proteostasis and autophagy | | | |  |
| *Mbnl1* and *Mbnl2* | *Mbnl1^-/-^/Mbnl2^+/-^* | | DM is caused by expansion of specific repeats in the *DMPK* or *CNBP* genes. These expansions sequester Mbnl proteins. In accordance, compound *Mbnl1/2* mutant recapitulate the DM-relevant features: NMJs appear immature and fragmented, while many muscle fibers are atrophied, split or contain central nuclei | PD (EL double KO) |
| *miR-1/206/133* | cKO | | *miR-206* is required for efficient regeneration of NMJs after acute nerve injury in a mouse model for ALS, but not in *mdx* mice, a commonly used model for muscle regeneration. The absence of *miR-206* alone has no effect in NMJ formation during development. In contrast, in triple *miR-1/206/133* cKO mice, AChR clusters are dispersed and reduced in number and size, while axons extend to extrasynaptic areas, leading to defects in NMJ formation and maintenance. No effects in muscle development are observed in any model | PD |
| *Spin1* | Skeletal muscle-specific KO (*Myf5-Cre*) | | Severe muscle deficiencies including sarcomere disorganization, fiber degeneration and necrosis, defective mitochondrial network and abnormal glycogen accumulation. The morphology of the presynaptic membrane is also altered, and nerve terminals contain vacuoles and a reduced number of vesicles | NL |
|  | | | | |
| Endocytosis, synaptic vesicle formation and neurotransmitter release | | | | |
| *Erbb2 and Erbb4* | Skeletal muscle-specific single *Erbb2* or double *Erbb2/Erbb4* KOs (*ACTA1-Cre*) | | Normal presynaptic terminals. However, postsynaptic terminals contain reduced levels of AChRs due to their migration to the perisynaptic membrane, thus causing postsynaptic fragmentation. Other scaffold proteins such as MuSK and rapsyn also relocate to perisynaptic areas. Synaptic transmission is mildly compromised | - |
| *Gabpa* | Skeletal muscle-specific KO (*ACTA1-Cre*) | | Two different skeletal muscle specific models show different phenotypes: mild NMJ morphological and functional defects with altered expression of some AChR subunits in one model, no phenotype in the second model | - |

**Table S3.** (*continued*)

| **Gene** | | **Model** | | | **Phenotype** | | | | **Lethality^1^** | | |
| --- | --- | --- | --- | --- | --- | --- | --- | --- | --- | --- | --- |
| *Nrg1/ARIA* | | | Heterozygous cKO | | | Reduced postsynaptic AChR density | | | | -  (EL cKO) | |
|  | | | | | | | | | | | |
| The DGC | | | | | | | | | | | |
| *Stnb1* and *Stnb2* | | Single *Snta1*, *Sntb1*, or *Sntb2*, double *Snta1/Sntb2* or triple *Snta1/Sntb1/Sntb2* cKOs | | | Disturbed NMJs morphology in *Snta1-/-* single mutants that is exacerbated in the double *Snta1^-/-^/Sntb2^-/-^* KOs, while single *Sntb2^-/-^* mice remain mostly unaltered. Thus, double mutants have few and aberrant functional folds, as well as reduced levels of AChRs, which in addition are abnormally distributed. Also, while dystrophin and dystrobrevin are still retained at the DGC complex, utrophin and nNOS are absent. The lack of nNOS could further contribute to postsynaptic membrane destabilization since it controls the expression of specific components of the integrin complex. Despite all these defects, synaptic transmission is mostly normal and no muscular dystrophy is observed. Triple *Snta1/Sntb1/Sntb2* KO mice have reduced protein expression and sarcolemmal targeting of dystrophin, and a much more severe outcome in muscle functional tests due to significant left ventricular cardiac hypertrophy | | | | - | | |
| *Utrn* | | Single Utrn, double Utrn/Dmd or triple Utrn/Dmd/Dnta cKOs | | | Single Utrn or Dmd mutants show reduced density of otherwise normally clustered AChR, and reduced number of junctional folds. The double Utrn/Dmd mutants exhibit abnormal AChR distribution and complete absence of postsynaptic folding. Only minimal electrophysiological changes in these three models. Defects in AChR distribution are due to the loss of ⍺-dystrobrevin association to the DGC, suggesting that this protein maintains the crests and depths of functional folds as molecularly distinct domains. Its absence in single Dnta and triple Utrn/Dmd/Dnta KOs leads to instability of postsynaptic specializations and eventually to NMJ deterioration | | | | AD: V or PD | | |
|  | | | | | | | | | | |  |
| Unknown | | | | | | | | | | |  |
| *Kalrn* | cKO or nervous system-specific KO (*Nes-Cre*) | | | Morphological abnormalities in pre and postsynaptic terminals, with decreased number of junctional folds in the latter case. Altered NMJ shape and sarcomere length in the target muscle | | | | SD: V or PD | | |  |
|  |  | | |  | | | |  | | |  |
| **Unknown** | | | | | | |  |  |  |  |  |
| Unknown | | | | | | | | | | |  |
| *Gas7* | Truncated, lowly expressed and unstable hypomorph | | | Decreased number of MNs, which show reduced terminal sprouting in adult NMJs. Changes in muscle fiber type composition and size only of slow muscles due to either MN innervation/denervation or defects in postsynaptic specialization | | | | - | | |  |
| *Skor2* | cKO | | | 90% KO pups die within 48 hours of birth due to failure to nurse, defective NMJ function, and/or respiratory failure | | | | PNL | | |  |

^1^ Key: AD: allele or model-dependent; EL: embryonic lethality; NL: neonatal lethality; PD: premature death; PNL: perinatal lethality; PL: postnatal lethality; SD: strain-dependent; V: viability.

^2^ See section *2.10. Other considerations* in the main text for further information regarding this line.
