# Supplementary material for: NMJ-related diseases beyond the congenital myasthenic syndromes
Source: Front Cell Dev Biol. 2023 Aug 4;11:1216726. doi: 10.3389/fcell.2023.1216726 (PMC10436495; doi:10.3389/fcell.2023.1216726)
Supplement: Supplementary file 3 [file DataSheet1.PDF]

Supplementary Material

NMJ-related diseases beyond the congenital myasthenic syndromes

Alejandra Navarro-Martínez, Cristina Vicente-García*, Jaime J. Carvajal

Table S1. Mouse models associated with NMJ-related abnormalities registered at the Mouse Genome Informatics MGI database (September, 2022). AEP: Abnormal Endplate Potential; AMEP: Abnormal Miniature Endplate Potential; ANSM: Abnormal Neuromuscular Synapse Morphology; FNSPrD: Failure of Neuromuscular Synapse Presynaptic Differentiation; FNSPoD: Failure of Neuromuscular Synapse Postsynaptic Differentiation.

Allele composition	Genetic background	Annotated MGI term
Adarb1$^{tm1.1Skwa}$/Adarb1$^{tm1.1Skwa}$<br>Tg(SLC18A3-cre)KMisa/0	C57BL/6	ANSM
Afg3l2^{par}/Afg3l2^{par}	Not Specified	ANSM AMEP
Agrn$^{Gt(Ex192)Byg}$/Agrn$^{Gt(Ex192)Byg}$	129P2/OlaHsd * C57BL/6	ANSM
Agrn^{nmf380}/Agrn^{nmf380}	C57BL/6J-Agrn^{nmf380}/J	ANSM
Agrn^{nmf380}/Agrn^{nmf380}	C57BL/6J * FVB/N	ANSM
Agrn^{nmf380}/Agrn^{nmf380}	C57BL/6J * DBA	ANSM
Agrn^{nmf380}/Agrn^{nmf380}	BALB/c * C57BL/6J	ANSM
Agrn^{nmf380}/Agrn^{nmf380}	C57BL/6J * CAST	ANSM
Agrn^{tm1Jrs}/Agrn^{tm1Jrs}	129S1/Sv * 129X1/SvJ	ANSM
Agrn^{tm1Rwb}/Agrn^{tm1Rwb}<br>Tg(Pax3-cre)1Joe/0	129S1/Sv * 129X1/SvJ * C57BL/6 * SJL	ANSM
Agrn^{tm3Jrs}/Agrn^{tm3Jrs}	129S1/Sv * 129X1/SvJ * C57BL/6	ANSM
Agrn^{tm4Jrs}/Agrn^{tm4Jrs}	Not Specified	FNSPrD
Agrn^{tm4Jrs}/Agrn^{tm4Jrs}<br>Chrna1^{tm1Klee}/Chrna1^{tm1Klee}<br>Chrna7^{tm1Bay}/Chrna7^{tm1Bay}	129S4/SvJae * 129S7/SvEvBrd * C57BL/6	FNSPrD
Als2^{tm1Jei}/Als2^{tm1Jei}	129P2/OlaHsd * C57BL/6J	ANSM
Aplp2^{tm1Dbo}/Aplp2^{tm1Dbo}<br>App$^{tm1.1Zhe}$/App$^{tm1.1Zhe}$<br>Chat$^{tm2(cre)Lowl}$/Chat^{+}	129S6/SvEvTac * 129S7/SvEvBrd * C57BL/6J	ANSM
Aplp2^{tm1Dbo}/Aplp2^{tm1Dbo}<br>App$^{tm1.1Zhe}$/App$^{tm1.1Zhe}$<br>Tg(Ckmm-cre)5Khn/0	129S7/SvEvBrd * C57BL/6J * FVB	ANSM AMEP
Aplp2^{tm1Dbo}/Aplp2^{tm1Dbo}<br>App$^{tm1.1Zhe}$/App$^{tm1.1Zhe}$<br>Tg(Nes-cre)1Kln/0	129S7/SvEvBrd * C57BL/6 * C57BL/6J * SJL	ANSM AMEP
Aplp2^{tm1Dbo}/Aplp2^{tm1Dbo}<br>App$^{tm1.2Zhe}$/App$^{tm1.2Zhe}$	129S7/SvEvBrd * C57BL/6J	ANSM

Table S1. (*continued*)

Allele composition	Genetic background	Annotated MGI term
Aplp2^{tm1Dbo}/Aplp2^{tm1Dbo}<br>App^{tm1Dbo}/App^{tm1Dbo}	129S7/SvEvBrd * C57BL/6J	ANSM
Aplp2^{tm1Dbo}/Aplp2^{tm1Dbo}<br>App^{tm1Dbo}/App$^{tm3.1Zhe}$	129/Sv * 129X1/SvJ * C57BL/6	ANSM
Aplp2^{tm1Dbo}/Aplp2^{tm1Dbo}<br>App$^{tm2.1Zhe}$/App$^{tm2.1Zhe}$	129S1/Sv * 129S7/SvEvBrd *<br>129X1/SvJ	ANSM
Aplp2^{tm1Dbo}/Aplp2^{tm1Dbo}<br>App$^{tm3.1Zhe}$/App$^{tm3.1Zhe}$	129/Sv * 129X1/SvJ * C57BL/6	ANSM
Aplp2^{tm1Dbo}/Aplp2^{tm1Dbo}<br>Lrp4^{tm1Her}/Lrp4^{tm1Her}	129S6/SvEvTac * 129S7/SvEvBrd<br>* C57BL/6J	ANSM
App^{tm1Dbo}/App^{tm1Dbo}<br>Lrp4^{tm1Her}/Lrp4^{tm1Her}	129S6/SvEvTac * 129S7/SvEvBrd<br>* C57BL/6J	ANSM
Atp2b1Tg(Thy1-<br>CHMP2B*)1Rene/Atp2b1+	C57BL/6 * DBA/2 * FVB/N	ANSM
Atp2b1Tg(Thy1-<br>CHMP2B*)1Rene/Atp2b1Tg(Thy1-<br>CHMP2B*)1Rene	C57BL/6 * DBA/2 * FVB/N	ANSM
Atp7atm1.1Mjp/Y<br>Mnx1tm4(cre)Tmj/Mnx1+	129S1/Sv * C57BL/6	ANSM
Basp1tm1Crni/Basp1tm1Crni	Not Specified	ANSM
Basp1tm2(Gap43)Crni/Basp1tm2(Gap43)Cr<br>ni	Not Specified	ANSM
Borcs5em1Jusb/Borcs5em1Jusb	C57BL/6-Borcs5em1Jusb	ANSM
Cacna1atm1Maag/Cacna1atm1Maag	129P2/OlaHsd * C57BL/6J	AMEP
Cacna2d2du/Cacna2d2du	C3H * DBA/2J	ANSM AMEP
Cacnb4lh/Cacnb4lh	BALB/cGn * C3H/HeSnJ *<br>C57BL/6JEi	ANSM
Cacng2stg/Cacng2stg	A/J * C3HeB/FeJ * C57BL/6J	ANSM
Chat/Slc18a3tm1.1Vpra/Chat+	129S4/SvJae * C57BL/6J	AMEP
Chat/Slc18a3tm1Vpra/Chat/Slc18a3tm1Vpr<br>a	129S4/SvJae * C57BL/6J	AMEP
Chat/Slc18a3tm1Vpra/Chat/Slc18a3tm1.1V<br>pra	129S4/SvJae * C57BL/6J	AMEP
Chat/Slc18a3tm1.1Vpra/Chat/Slc18a3tm1.1<br>Vpra	129S4/SvJae * C57BL/6J	ANSM AMEP
Chat/Slc18a3tm1.1Vpra/ Slc18a3+	129S4/SvJae * C57BL/6J	AMEP
Chattm1.1Jrs/Chattm1.1Jrs	Not Specified	ANSM
Chattm1Fhg/Chattm1Fhg	129S2/SvPas * C57BL/6	ANSM
Chattm1Jrs/Chattm1Jrs<br>Tg(CAG-cre/Esr1*)1Lbe/0	129S1/Sv * 129X1/SvJ * C57BL/6<br>* SJL	ANSM
Chattm2(cre)Lowl/Chat+<br>Mapttm2(FUS)Neas/Mapt+	B6.129-Mapttm2(FUS)Neas<br>Chattm2(cre)Lowl	ANSM

Table S1. (*continued*)

Allele composition	Genetic background	Annotated MGI term
Chattm2(cre)Lowl/Chat+ Mapttm3(FUS)Neas/Mapt+	B6.129-Mapttm3(FUS)Neas Chattm2(cre)Lowl	ANSM
Chchd10em8Lutzy/Chchd10+	57BL/6NJ-Chchd10em8Lutzy/J	ANSM
Chrna1tm1Klee/Chrna1tm1Klee	129S4/SvJae * C57BL/6	ANSM AEP AMEP
Chrna7tm1Bay/Chrna7tm1Bay	129S7/SvEvBrd	FNSPrD
Chrnb1tm1Sjb/Chrnb1tm1Sjb	Not Specified	ANSM AMEP
Chrnetm1Jrs/Chrnetm1Jrs	129S1/Sv * 129X1/SvJ * C57BL/6	ANSM
Chrnetm1Vwi/Chrnetm1Vwi	129P2/OlaHsd * C57BL/6	ANSM AMEP
Chrnetm2Vwi/Chrnetm2Vwi	129P2/OlaHsd * C57BL/6	ANSM AMEP
Chrngtm1(Chrne)Mknn/Chrngtm1(Chrne)Mknn	129/Sv * C57BL/6	AEP
Chrngtm1Mtak/Chrngtm1Mtak	129X1/SvJ * C57BL/6	ANSM AMEP
Chrngtm2(Chrne)Vwi/Chrngtm2(Chrne)Vwi	C57BL/6	ANSM AMEP
Clip3tm1.1Ics/Clip3tm1.1Ics	C57BL/6	ANSM
Clp1tm1.1Pngr/Clp1tm1.1Pngr	CBA.Cg-Clp1tm1.1Pngr	ANSM
Clp1tm1.1Pngr/Clp1tm1.1Pngr	B6.Cg-Clp1tm1.1Pngr	ANSM
Col13a1tm1Pih/Col13a1tm1Pih	129S1/Sv * 129X1/SvJ	AEP
Col13a1tm2Pih/Col13a1tm2Pih	129S6/SvEvTac * C57BL/6	AEP
Col13a1tm3.1Pih/Col13a1tm3.1Pih	129S1/Sv * 129X1/SvJ * C57BL/6	ANSM AEP AMEP
Col13a1tm3.1Pih/Col13a1tm3.1Pih	129S1/Sv * 129X1/SvJ	ANSM
Col13a1tm4.1Pih/Col13a1tm4.1Pih	C57BL/6	ANSM AEP AMEP
Colqtm1Jrs/Colqtm1Jrs	129S1/Sv * 129X1/SvJ	ANSM
Colqtm2Jrs/Colqtm2Jrs	129S1/Sv * 129X1/SvJ	ANSM
Cpeb4tm1a(EUCOMM)Wtsi/Cpeb4tm1a(EUCOMM)Wtsi	C57BL/6 * C57BL/6Ntac	ANSM
Csnk2btm1.1Bb/Csnk2btm1.1Bb Tg(ACTA1-cre)79Jme/?	129S2/SvPasCrl * C57BL/6J * SJL	ANSM AMEP
Dag1tm2Kcam/Dag1tm2Kcam Tg(Pax3-cre)1Joe/0	129S1/Sv * 129X1/SvJ * C57BL/6 * SJL	ANSM
Dag1tm4.1Kcam/Dag1tm4.1Kcam	129S1/Sv * 129X1/SvJ	ANSM
Dctn1tm1Cai/Dctn1+	129X1/SvJ * C57BL/6 * FVB/N	ANSM
Dctn1tm2.1Cai/Dctn1tm2.1Cai Tg(Thy1-cre)1Vln/?	129S4/SvJaeSor * 129X1/SvJ * C57BL/6 * FVB/N	ANSM
Dhtkd1tm1Zgwg/Dhtkd1tm1Zgwg	C57BL/6	ANSM
Dmdmdx/Y	C57BL/10ScSn	ANSM
Dmdmdx/Y Dtnatm1Jrs/Dtnatm1Jrs	129X1/SvJ * C57BL/10ScSn	ANSM

Table S1. (*continued*)

Allele composition	Genetic background	Annotated MGI term
Dmdmdx/Y Dtnatm1Jrs/Dtnatm1Jrs Utrntm1Jrs/Utrntm1Jrs	129S1/Sv * 129X1/SvJ * C57BL/10ScSn	ANSM
Dmdmdx/Dmdmdx	C57BL/10ScSn	ANSM
Dmdmdx/Dmdmdx Utrntm1Jrs/Utrntm1Jrs	129S1/Sv * 129X1/SvJ * C57BL/10ScSn	ANSM
Dmdmdx/Dmdmdx Utrntm1Ked/Utrntm1Ked	129S1/Sv * 129X1/SvJ * C57BL/6 * C57BL/10ScSn * DBA	ANSM
Dnajc5tm1Sud/Dnajc5tm1Sud	129S6/SvEvTac	ANSM AEP
Dnm2tm2.1Ics/Dnm2+	C57BL/6J	ANSM
Dnmt3atm1Jae/Dnmt3atm1Jae Tg(Nes-cre)1Atp/0	129S4/SvJae * C57BL/6 * FVB/N	ANSM
Dok7tm1Yyam/Dok7tm1Yyam	129P2/OlaHsd * C57BL/6	ANSM
Dok7tm2Yyam/Dok7tm2Yyam	129P2/OlaHsd * C57BL/6J	ANSM
DstTg4/DstTg4	C57BL/6 * CD-1	ANSM
Dtnatm1Jrs/Dtnatm1Jrs	129X1/SvJ * C57BL/6	ANSM
Dtnatm1Jrs/Dtnatm1Jrs Utrntm1Jrs/Utrntm1Jrs	129S1/Sv * 129X1/SvJ	ANSM
Dync1h1tm1.1Sjki/Dync1h1+	129 * 129S1/SvImJ * C57BL/6 * C57BL/6J	ANSM
Ecel1tm1Hiki/Ecel1tm1Hiki	B6.129-Ecel1tm1Hiki	ANSM
Ecel1tm1Hiki/Ecel1tm1Hiki Tg(Hlxb9-GFP)1Tmj/0	129S1/Sv * 129X1/SvJ * C57BL/6 * CBA	ANSM
Ednrbs-1Acrg/Ednrbs-9ThW	101/Rl * C3H/Rl * C57BL/6J	ANSM
Eef1a2wst/Eef1a2wst	HRS/J	ANSM AEP
Egr2tm2Jmi/Egr2tm2Jmi	B6.Cg-Egr2tm2Jmi	ANSM
Erbb2tm3(Erbb2)Mul/Erbb2tm1Mll Tg(ACTA1-cre)1Mll/0	129S1/Sv * 129X1/SvJ * C57BL/6	ANSM
Fat1Gt(KST249)Byg/Fat1Gt(KST249)Byg Tg(Myl1-lacZ)1Ibdml/0	129P2/OlaHsd	ANSM
Fat1tm1.2Fhel/Fat1tm1.2Fhel Tg(Myl1-lacZ)1Ibdml/0	129S6/SvEvTac * BALB/cJ * C57BL/6J * SJL	ANSM
Fbxo45tm1Kei/Fbxo45tm1Kei	B6.129P2-Fbxo45tm1Kei	ANSM
Fgfbp1tm1Gvdz/Fgfbp1tm1Gvdz	129S5/SvEvBrd	ANSM
Fgfbp1tm1Gvdz/Fgfbp1tm1Gvdz Tg(SOD1*G93A)1Gur/0 Tg(Thy1-YFP)16Jrs/0	129S5/SvEvBrd * C57BL/6J * CBA * SJL	ANSM
Fgfbp1tm1Gvdz/Fgfbp1tm1Gvdz Tg(Thy1-YFP)16Jrs/0	129S5/SvEvBrd * C57BL/6J * CBA	ANSM
Fustm1.1Emcf/Fus+	C3H * C57BL/6J * C57BL/6N	ANSM

Table S1. (*continued*)

Allele composition	Genetic background	Annotated MGI term
Fustm1a(EUCOMM)Wtsi/Fustm1c(EUCOMM)Wtsi Mapttm3.1(FUS)Neas/Mapt+ Ndor1Tg(UBC-cre/ERT2)1Ejb/0	129 * C3H * C57BL/6 * C57BL/6N	ANSM
Gabpatm1Pjh/Gabpatm1Pjh Tg(ACTA1-cre)79Jme/0	129X1/SvJ * C57BL/6 * SJL	ANSM
GarsC201R/Gars+	BALB/cAnN * C3H/HeH * C57BL/6J	ANSM
GarsC201R/Gars+ Nrcamm1J/Nrcamm1J	BALB/cAnN * C3H/HeH * C57BL/6J	ANSM
GarsC201R/Gars+ Scn8am10J/Scn8a+	BALB/cAnN * C3H/HeJ * FVB/NJ	ANSM
GarsC201R/Gars+ Tg(Thy1-YFP)16Jrs/?	BALB/cAnN * C3H/HeH * C57BL/6J * CBA	ANSM
GarsC201R/GarsC201R Tg(CAG-GARS)DRwb/0	BALB/cAnN * C3H/HeH * C57BL/6J * FVB/N	ANSM
GarsC201R/GarsGt(XM256)6Byg Tg(CAG-GARS)DRwb/0	129P2/OlaHsd * BALB/cAnN * C3H/HeH * C57BL/6J * FVB/N	ANSM
GarsC201R/GarsNmf249	BALB/cAnN * C3H/HeH * C57BL/6J	ANSM
GarsC201R/GarsNmf249 Tg(CAG-GARS)DRwb/0	BALB/cAnN * C3H/HeH * C57BL/6J * FVB/N	ANSM
Garsem1Rwb/Garsem2Rwb	C57BL/6NJ * FVB/NJ	ANSM
GarsNmf249/Gars+	C57BL/6J-GarsNmf249/J	ANSM
GarsNmf249/Gars+	C57BL/6J * CAST/Ei	ANSM
GarsNmf249/Gars+ Scn8am10J/Scn8a+	C57BL/6J * FVB/NJ	ANSM
GarsNmf249/Gars+ Tg(CAG-GARS)DRwb/0	C57BL/6J * CAST/Ei * FVB/N	ANSM
GarsNmf249/Gars+ Tg(Thy1-YFP)16Jrs/?	C57BL/6J * CAST/Ei * CBA/J	ANSM AEP
Gas7tm1Slch/Gas7tm1Slch	B6.129-Gas7tm1Slch	ANSM
Gdap1tm1.2Geno/Gdap1tm1.2Geno	129 * C57BL/6	ANSM
Gfpt1tm1c(EUCOMM)Wtsi/Gfpt1tm1c(EUCOMM)Wtsi Tg(Ckmm-cre)5Khn/0	C57BL/6J * C57BL/6N * SJL/J	ANSM
Gphntm1Jrs/Gphntm1Jrs	129S1/Sv * 129X1/SvJ * C57BL/6	ANSM
Gt(ROSA)26Sortm1.1(CAG-PLS3,-GFP)Bwir/Gt(ROSA)26Sor+ Smn1tm1Hung/Smn1+ Tg(SMN2)2Hung/0	B6N.Cg-Gt(ROSA)26Sortm1.1(CAG-PLS3,-GFP)Bwir Smn1tm1Hung Tg(SMN2)2Hung	ANSM
Gt(ROSA)26Sortm1.1(CAG-PLS3,-GFP)Bwir/Gt(ROSA)26Sor+ Smn1tm1Hung/Smn1tm1Hung Tg(SMN2)2Hung/0	B6N.Cg-Gt(ROSA)26Sortm1.1(CAG-PLS3,-GFP)Bwir Smn1tm1Hung Tg(SMN2)2Hung	ANSM

Table S1. (*continued*)

Allele composition	Genetic background	Annotated MGI term
Gt(ROSA)26Sortm407(H1/tetO-RNAi:Large)Arte/Gt(ROSA)26Sor+	B6.Cg-Gt(ROSA)26Sortm407(H1/tetO-RNAi:Large)Arte	ANSM
Hspg2tm1.1Soni/Hspg2tm1.1Soni	129S/SvEv * C57BL/6	ANSM AEP AMEP
Hspg2tm1Nid/Hspg2tm1Nid	129X1/SvJ * C57BL/6	ANSM
Hspg2tm1Soni/Hspg2tm1Soni	129S/SvEv * DBA/2J	ANSM
Ighmbp2em1Cll/Ighmbp2em1Cll	FVB/NJ-Ighmbp2em1Cll	ANSM
Ighmbp2nmd-2J/Ighmbp2nmd-2J	B6.BKS-Ighmbp2nmd-2J/J	ANSM
Itgb1tm1Lscd/Itgb1tm1Ref Tg(PLAT-cre)116Sdu/0	Not Specified	ANSM
Kalrntm2.1Npl/Kalrntm2.1Npl	B6.Cg-Kalrntm2.1Npl	ANSM
Kyky/Kyky	Not Specified	ANSM
Lama4tm1Ktry/Lama4tm1Ktry Lama5tm2Jhm/Lama5tm2Jhm Tg(ACTA1-cre)1Mll/0	129S1/Sv * 129X1/SvJ	ANSM
Lama5tm1Jhm/Lama5tm1Jhm Tg(ACTB-Lama5/LAMA1)1Jhm/0	129S1/Sv * 129X1/SvJ * C57BL/6 * CBA	ANSM
Lama5tm2Jhm/Lama5tm2Jhm Tg(ACTA1-cre)1Mll/0	129S1/Sv * 129X1/SvJ	ANSM
Lamb2tm1Jrs/Lamb2tm1Jrs	129S1/Sv * 129X1/SvJ	ANSM AMEP
Lgals1tm1Rob/Lgals1tm1Rob	129S/SvEv	ANSM
Lrp4mitt/Lrp4mitt	C57BL/6	ANSM FNSPrD FNSPoD
Lrp4mte/Lrp4mte	C57BL/6	ANSM FNSPrD FNSPoD
Lrp4tm1.1Line/Lrp4tm1.1Line Mnx1tm4(cre)Tmj/Mnx1+ Tg(ACTA1-cre)79Jme/0	129S/SvEv * 129S1/Sv * C57BL/6 * C57BL/6J * SJL	ANSM
Lrp4tm1.1Line/Lrp4tm1.1Line Tg(ACTA1-cre)79Jme/0	129S/SvEv * C57BL/6 * C57BL/6J * SJL	ANSM AEP AMEP
Lrp4tm1Her/Lrp4tm1Her	129S/SvEvBrd * 129S6/SvEvTac * C57BL/6J	ANSM
Lrp4tm2Her/Lrp4tm2Her	129S/SvEv * C57BL	ANSM
Lrp4tm2Her/Lrp4tm2Her	129S/SvEvBrd * C57BL/6J	ANSM FNSPrD FNSPoD
Lrsam1Gt(RRK461)Byg/Lrsam1+	129P2/OlaHsd	ANSM
Lrsam1Gt(RRK461)Byg/Lrsam1Gt(RRK461)Byg	129P2/OlaHsd	ANSM
Maddtm1Ytk/Maddtm1Ytk	129X1/SvJ * C57BL/6 * DBA/2	ANSM
Mapttm2.1(FUS)Neas/Mapt+	B6J.129P2(129S)-Mapttm2.1(FUS)Neas	ANSM
Mapttm3.1(FUS)Neas/Mapt+	B6J.129P2(129S)-Mapttm3.1(FUS)Neas	ANSM
Mapttm3.1(FUS)Neas/Mapttm3.1(FUS)Neas	B6J.129P2(129S)-Mapttm3.1(FUS)Neas	ANSM
Matr3em1Tcp/Matr3em1Tcp	C57BL/6J-Matr3em1Tcp	ANSM

Table S1. (*continued*)

Allele composition	Genetic background	Annotated MGI term
Mbnl1tm1Sws/Mbnl1tm1Sws Mbnl2tm1.1Sws/Mbnl2+	129S1/Sv * 129S1/SvImJ * C57BL	ANSM
Mdga2Tg(Prnp-PFN1*G118V)838Kiaei/Mdga2+	C57BL/6N	ANSM
Meox2tm1(cre)Sor/Meox2+ Tg(CAG-lacZ,-FUS*R521G,-EGFP)682Gyu/0	129S4/SvJaeSor * C57BL/6	ANSM
Meox2tm1(cre)Sor/Meox2+ Tg(CAG-lacZ,-FUS,-EGFP)629Gyu/0	129S4/SvJaeSor * C57BL/6	ANSM
Mettm1Cpo/Mettm1Sst Tg(Nes-cre)1Kln/0	129P2/OlaHsd * 129S1/Sv * 129X1/SvJ * C57BL/6 * SJL	ANSM
Mir206tm1Eno/Mir206tm1Eno Tg(SOD1*G93A)dl1Gur/0	129S/SvEv * C57BL/6 * SJL	ANSM
Mir218-1em1Slp/Mir218-1em1Slp Mir218-2em1Slp/Mir218-2em1Slp	Not Specified	FNSPrD
Mmp3tm1Mrl/Mmp3tm1Mrl	129S7/SvEvBrd or 129S7/SvEvBrd * C57BL/6	ANSM AMEP
Mnx1tm4(cre)Tmj/Mnx1+	129S1/Sv	ANSM
Mnx1tm4(cre)Tmj/Mnx1+ Mycbp2tm1Adia/Mycbp2tm1Adia	129S1/Sv * C57BL/6	ANSM
Musktm1.1Vwi/Musktm2Vwi	Not Specified	ANSM AEP AMEP
Musktm1.2Ics/Musktm1.2Ics	BALB/cN * C57BL/6NTac	ANSM
Musktm1Gdy/Musktm1Gdy	129P2/OlaHsd * C57BL/6	ANSM FNSPrD FNSPoD
Musktm1Vwi/Musktm1.1Vwi Tg(Ckmm-cre)5Khn/0	C57BL/6J * FVB	ANSM
Musktm1Vwi/Musktm1Vwi Tg(Ckmm-cre)5Khn/0	C57BL/6J * FVB	ANSM
Mycbp2tm1.1Adia/Mycbp2tm1.1Adia	C57BL/6 * FVB/N	ANSM
Myf5tm3(cre)Sor/Myf5+ Spin1tm1.1Rosc/Spin1tm1.1Rosc	129S4/SvJaeSor * C57BL/6N * C57BL/6NTac	ANSM
NbeaTg(GH1)240BNec/NbeaTg(GH1)240BNec	C57BL/6 * SJL	AMEP
Nemfem8Cx/Nemfem8Cx	C57BL/6J-Nemfem8Cx/Cx	ANSM
Nemfpdft/Nemfpdft	B6(C3)-Nemfpdft/Cx	ANSM
Nemftvrm116/Nemftvrm116	C57BL/6J-Nemftvrm116/PjnCx	ANSM
Nestm1Nagy/Nestm1Nagy	129S6/SvEvTac * C57BL/6NCr or 129S6/SvEvTac * C57BL/6NCr * ICR	ANSM
Nkx6-2tm2(cre)Ercs/Nkx6-2+ Rettm1.1Pern/Rettm1.1Pern	129P2/OlaHsd * C57BL/6 * SJL	ANSM
nmf67/nmf67	C57BL/6J-nmf67/J	ANSM
Nrcamm1J/Nrcamm1J Sh3tc2m1J/Sh3tc2m1J	B6.Cg-Nrcamm1J Sh3tc2m1J/GrsrRwb	ANSM
Nrg1tm1Leth/Nrg1+	129S1/Sv * C57BL/6	ANSM AEP AMEP

Table S1. (*continued*)

Allele composition	Genetic background	Annotated MGI term
Nrg1tm1Cbm/Nrg1tm3Cbm Isl2tm1Arbr/Isl2+	129P2/OlaHsd * 129S7/SvEvBrd * C57BL/6J	ANSM
Olig2tm1(cre)Tmj/Olig2+ Smn1tm1Jme/Smn1tm1Msd Grm7Tg(SMN2)89Ahmb/Grm7Tg(SMN2)89Ahmb	129 * 129P2/OlaHsd * FVB/N	ANSM AMEP
Pex10m1Nisw/Pex10m1Nisw	129S1.B6-Pex10m1Nisw	ANSM AEP
Plaaem1Pmi/Plaaem1Pmi	C57BL/6J-Plaaem1Pmi	ANSM AMEP
Plaaem1Pmi/Plaatm1(NCOM)Cmhd	C57BL/6J * C57BL/6NTac or C57BL/6J * C57BL/6NTac * CD-1	ANSM
Prkcqtm1Litt/Prkcq+	B6.129P2-Prkcqtm1Litt	ANSM
Prkcqtm1Litt/Prkcqtm1Litt	B6.129P2-Prkcqtm1Litt	ANSM AEP
Psmf1tm1c(EUCOMM)Hmgu/Psmf1tm1c(EUCOMM)Hmgu Mnx1tm4(cre)Tmj/Mnx1+	129S1/Sv * C57BL/6J * C57BL/6N	ANSM
Rab18Gt(EUCE0233a03)Hmgu/Rab18Gt(EUCE0233a03)Hmgu	129P2/OlaHsd * C57BL/6J	ANSM
Rapsnem1Gan/Rapsnem1Gan	C57BL/6J-Rapsnem1Gan	ANSM AMEP
Rapsnem1Gan/Rapsntm1Jrs	129S1/Sv * 129X1/SvJ * C57BL/6J	ANSM AMEP
Rapsntm1Jrs/Rapsntm1Jrs	129S1/Sv * 129X1/SvJ	ANSM FNSPoD
Rer1Gt(RRN159)Byg/Rer1+	129P2/OlaHsd	ANSM
Rettm1.2Pern/Rettm1.2Pern	129P2/OlaHsd * BALB/c * C57BL/6 * SJL	ANSM
Rims1tm1Sud/Rims1tm1Sud Rims2tm1.1Schc/Rims2tm1.1Schc	129P2/OlaHsd * 129S1/Sv * 129S4/SvJae * 129X1/SvJ	AEP AMEP
Rnd3Gt(OST364657)Lex/Rnd3Gt(OST364657)Lex	129S5/SvEvBrd * C57BL/6J	ANSM
Scn8amed/Scn8amed	PCT	ANSM AEP AMEP
Scyl1mdf/Scyl1mdf	B6C3Fe a/a-Scyl1mdf/J	ANSM
Setxtm1.1Als/Setx+	C57BL/6J	ANSM
Skor2Tn(sb-Tyr)1799B.CA3Ove/Skor2Tn(sb-Tyr)1799B.CA3Ove	FVB/N	ANSM
Skor2Tn(sb-Tyr)1799B.CA7Ove/Skor2Tn(sb-Tyr)1799B.CA7Ove	FVB/N	ANSM
Slc18a3tm1Mca/Slc18a3+	129S4/SvJae * C57BL/6J	AMEP
Slc18a3tm1Mca/Slc18a3tm1Mca	129S4/SvJae * C57BL/6J	AEP AMEP
Slc5a7tm1Rbl/Slc5a7tm1Rbl	129S6/SvEvTac * C57BL/6	ANSM
Slc6a5m1J/Slc6a5m1J	129S2/SvPas * NOD	ANSM
Slc16a3tm1Tac/Slc16a3tm1Tac	B6NTac.Cg-Slc16a3tm1Tac/Tac	ANSM

Table S1. (*continued*)

Allele composition	Genetic background	Annotated MGI term
Slc25a46atc/Slc25a46atc	B6.Cg-Slc25a46atc	ANSM
Smn1tm1Cdid/Smn1tm1Cdid Grm7Tg(SMN2)89Ahmb/Grm7+	129 * C57BL/6 * FVB	ANSM
Smn1tm1Hung/Smn1tm1Hung Tg(SMN2)2Hung/0	B6N.Cg-Smn1tm1Hung Tg(SMN2)2Hung	ANSM
Smn1tm1Hung/Smn1tm1Hung Tg(SMN2)2Hung/0	FVB.Cg-Smn1tm1Hung Tg(SMN2)2Hung/J	ANSM
Smn1tm1Hung/Smn1tm1Hung Tg(SMN2)2Hung/0	129P2/OlaHsd * C57BL/6N * FVB/N	ANSM AEP
Smn1tm1Msd/Smn1tm1Msd Grm7Tg(SMN2)89Ahmb/Grm7Tg(SMN2)89Ahmb	FVB.Cg-Grm7Tg(SMN2)89Ahmb Smn1tm1Msd/J	ANSM
Smn1tm1Msd/Smn1tm1Msd Grm7Tg(SMN2)89Ahmb/Grm7Tg(SMN2)89Ahmb Tg(SMN2*delta7)4299Ahmb/Tg(SMN2*delta7)4299Ahmb	FVB.Cg-Grm7Tg(SMN2)89Ahmb Smn1tm1Msd Tg(SMN2*delta7)4299Ahmb/J	ANSM
Smn1tm1Msd/Smn1tm1Msd Grm7Tg(SMN2)89Ahmb/Grm7Tg(SMN2)89Ahmb Tg(SMN2*delta7)4299Ahmb/Tg(SMN2*delta7)4299Ahmb	FVB.Cg-Grm7Tg(SMN2)89Ahmb Smn1tm1Msd Tg(SMN2*delta7)4299Ahmb	ANSM
Smn1tm1Msd/Smn1tm1Msd Tg(SMN1*A2G)2023Ahmb/0 Grm7Tg(SMN2)89Ahmb/Grm7+	129P2/OlaHsd * FVB/N	ANSM
Smn1tm1Msd/Smn1tm1Msd Tg(SMN1-SMN2*)16Cll/0 Grm7Tg(SMN2)89Ahmb/Grm7Tg(SMN2)89Ahmb	129P2/OlaHsd * C57BL/6 * FVB/N	ANSM
Smn1tm1Msd/Smn1tm1Msd Tg(SMN2)11Tro/0 Tg(SMN2)46Tro/0	B6.Cg-Tg(SMN2)11Tro Tg(SMN2)46Tro Smn1tm1Msd/J	ANSM
Smn1tm5(Smn1/SMN2)Mrph/Smn1tm5(Smn1/SMN2)Mrph	129S6/SvEvTac * C57BL/6J * C57BL/6NTac	ANSM AEP AMEP
Snap25tm1Mcw/Snap25tm1Mcw	C57BL/6	AEP
Snta1tm1Scf/Snta1tm1Scf	129P2/OlaHsd * C57BL/6	ANSM
Snta1tm1Scf/Snta1tm1Scf Sntb2tm1Scf/Sntb2tm1Scf	129P2/OlaHsd * C57BL/6	ANSM AMEP
Stac3tm1a(KOMP)Wtsi/Stac3tm1a(KOMP)Wtsi	C57BL/6N	ANSM AMEP
Stxbp5ltm1.1Aljg/Stxbp5ltm1.1Aljg	C57BL/6	AEP
Syne1tm1Rexu/Syne1tm1Rexu	129 * C57BL/6	ANSM
Syne1tm1Rexu/Syne1tm1Rexu Syne2tm1Rexu/Syne2+	129 * C57BL/6	ANSM
Syne1tm1Rexu/Syne1tm1Rexu Syne2tm1Rexu/Syne2tm1Rexu	129 * C57BL/6	ANSM

Table S1. (*continued*)

Allele composition	Genetic background	Annotated MGI term
Syt2m1Ingm/Syt2m1Ingm	Not Specified	AMEP
Syt2tm1Sud/Syt2tm1Sud	B6.129-Syt2tm1Sud	AMEP
Tardbptm1.1Sobue/Tardbptm1.1Sobue Tg(SLC18A3-cre)Misa/0	129S6/SvEvTac * C57BL/6 * C57BL/6J * C57BL/6NTac	ANSM
Tardbptm3.1Ckjs/Tardbp+	C57BL/6J	ANSM
Tg(C9orf72)500Lpwr/0	FVB/NJ-Tg(C9orf72)500Lpwr	ANSM
Tg(Ckm-Chrnd*S262T)40Cgz/0	C57BL/6 * DBA/2	AEP AMEP
Tg(Ckm-Chrne*L269F)5Cgz/?	FVB/NJ	ANSM AEP AMEP
Tg(Ckm-Syne2)1Rexu/0	FVB	ANSM
Tg(DMWD,DMPK*,SIX5)328Ggo /Tg(DMWD,DMPK*,SIX5)328Ggo	C57BL/6 * DBA/2	ANSM
Tg(NEFH-tTA)8Vle/0 Tg(tetO-TARDBP*)4Vle/0	C3H/HeJ * C57BL/6J	ANSM
Tg(Prnp-FUS)WT3Cshw/Tg(Prnp-FUS)WT3Cshw	C57BL/6 * Crl:CD-1(ICR)	ANSM
Tg(Prnp-FUS*R521C)3313Ejh/0	C57BL/6 * SJL	ANSM
Tg(Prnp-TARDBP)96Dwc/0 Tg(Prnp-TARDBP*Q331K)31Dwc/0	C3H * C57BL/6	ANSM
Tg(Prnp-TARDBP*Q331K)31Dwc/?	C3H * C57BL/6	ANSM
Tg(Prnp-TARDBP*Q331K)103Dwc/?	C57BL/6 * C3H	ANSM
Tg(TARDBP)#Jpj/0	C3H * C57BL/6	ANSM
Tg(TARDBP*G348C)#Jpj/0	C3H * C57BL/6	ANSM
Tg(tetO-NEFL)173.2Jpj/0 Tg(THY1-tTA)177Jpj/0	C3H * C57BL/6	ANSM
Tg(Thy1-DCTN1*G59S)M2Pcw/?	C57BL/6 * SJL	ANSM
Tg(Thy1-HSPB1*P182L)#Lvdb/0	FVB/N	ANSM
Tg(Thy1-HSPB1*S135F)#Lvdb/0	FVB/N	ANSM
Tg(Thy1-Snca)1S13Putt/?	C57BL/6	ANSM
Tg(Thy1-SNCA*A53T)9813Putt/0	C57BL/6	ANSM
Tg(Thy1-SOD1*G93A)T1Hgrd/0 Tg(Thy1-SOD1*G93A)T3Hgrd/0	C57BL/6 * CBA * FVB	ANSM
Tg(Thy1-SOD1*G93A)T3Hgrd/Tg(Thy1-SOD1*G93A)T3Hgrd	C57BL/6 * CBA * FVB	ANSM
Tmem106bem1Damme/Tmem106bem1Damme	C57BL/6-Tmem106bem1Damme	ANSM
Tmem184bGt(IST10294F4)Tigm/Tmem184bGt(IST10294F4)Tigm	C57BL/6N	ANSM
Uchl1tm1Dgen/Uchl1tm1Dgen	B6.129P2-Uchl1tm1Dgen/Mmnc	ANSM AEP AMEP
Usp14ax-J/Usp14ax-J	B6.Cg-Usp14ax-J	ANSM AMEP
Usp14nmf375/Usp14nmf375	C.B6-Usp14nmf375	ANSM

Table S1. (*continued*)

**Allele composition**	**Genetic background**	**Annotated MGI term**
Utp14bjsd/Utp14bjsd	C57BL/6J	FNSPoD
Utrntm1Jrs/Utrntm1Jrs	129S1/Sv * 129X1/SvJ	ANSM
Utrntm1Ked/Utrntm1Ked	129S1/Sv * 129X1/SvJ * C57BL/6 * DBA	ANSM AMEP
Vapbtm1.1Tsud/Vapb+	129S7/SvEvBrd	ANSM
Vapbtm1.1Tsud/Vapbtm1.1Tsud	129S7/SvEvBrd	ANSM

Table S2. Unique genes involved in the mouse models associated with NMJ-related abnormalities registered at the Mouse Genome Informatics MGI database (September, 2022). Associated neuromuscular diseases, as described at www.musclegenetable.fr, are indicated with a code (disease group and disease separated by a dot), the name of the disease and the type of inheritance. Note that congenital myasthenic syndromes are clustered in group 11 and associated genes are marked with an asterisk *. AD: Autosomal Dominant; AR: Autosomal Recessive; N/A: Not Applicable; XR: X-linked.

Gene	Neuromuscular disease (Code: Disease – (Inheritance))
Adarb1/Adar2	N/A
Afg3l2	13.25: Spinocerebellar ataxia 28 - (AD)<br>15.80: Spastic ataxia 5 autosomal recessive - (AR)
*Agrn**	11.16: Myasthenic syndrome, congenital, 8, with pre- and postsynaptic defects - (AR)<br>16.31: Fetal akinesia deformation related to AGRN- (AR)
Als2	12.45: Amyotrophic lateral sclerosis 2 juvenile - (AR)<br>15.69: Infantile-onset ascending spastic paraplegia- (AR)
Aplp2	N/A
App	N/A
Atp7a	12.42: Spinal muscular atrophy, distal, X-linked, 3 - (XR)
Basp1/Cap23	N/A
Borcs5	N/A
C9orf72	12.72: Amyotrophic lateral sclerosis and/or frontotemporal dementia - (AD)
Cacna1a	7.9: Acetazolamide responsive hereditary paroxysmal cerebellar ataxia - (AD)<br>7.10: Episodic ataxia type-2 - (AD)<br>13.6: Spinocerebellar ataxia 6 - (AD)<br>13.45: Episodic ataxia type-2 ,and familial hemiplegic migraine - (AD)<br>13.50: Acetazolamide responsive hereditary paroxysmal cerebellar ataxia - (AD)
Cacna2d2	N/A
Cacnb4	13.47: Episodic ataxia type-5 - (AD)
Cacng2	N/A
*ChAT**	11.13: Myasthenic syndrome, congenital, 6, presynaptic - (AR)
Chchd10	12.36: Spinal muscular atrophy, Jokela type - (AD)<br>12.73: Amyotrophic lateral sclerosis and/or frontotemporal dementia - (AD)<br>16.62: Isolated mitochondrial myopathy - (AD)
Chmp2b	12.60: Amyotrophic lateral sclerosis 17 - (AD)
*Chrna1**	11.1: Myasthenic syndrome, congenital, 1A, Slow-channel - (AD)<br>11.5: Myasthenic syndrome, congenital, 1B, Fast-channel - (AR)
Chrna7	N/A
*Chrnb1**	11.2: Myasthenic syndrome, congenital, 2A, Slow-channel - (AD)<br>11.6: Myasthenic syndrome, congenital, 2B, Fast-channel - (AR)<br>11.9: Myasthenic syndrome, congenital, 2C, associated with acetylcholine receptor deficiency - (AR)
*Chrnd**	11.3: Myasthenic syndrome, congenital, 3A, Slow-channel - (AD)<br>11.7: Myasthenic syndrome, congenital, 3B, Fast-channel - (AR<br>11.10: Myasthenic syndrome, congenital, 3C, associated with acetylcholine receptor deficiency - (AR)
*Chrne**	11.4: Myasthenic syndrome, congenital, 4A, Slow-channel - (AD, AR)<br>11.8: Myasthenic syndrome, congenital, 4B, Fast-channel - (AR)<br>11.11: Myasthenic syndrome, congenital, 4C, associated with acetylcholine receptor deficiency - (AR)

Table S2. (*continued*)

Gene	Neuromuscular disease (Code: Disease – (Inheritance))
*Chrng**	11.35: Escobar syndrome (multiple pterygium syndrome) - (AR)
Clip3/CLIPR-59	N/A
Clp1	N/A
*Col13a1**	11.27: Congenital myasthenic syndrome type 19 - (AR)
*Colq**	11.12: Myasthenic syndrome, congenital, 5 - (AR)
Cpeb4	N/A
Csnk2b	N/A
Dag1	1.45: Limb-Girdle Muscular Dystrophy type R16 - (AR)<br>2.38: Congenital muscular dystrophy with hypoglycosylation of dystroglycan type A9 - (AR)
Dctn1	12.71: Amyotrophic lateral sclerosis related to dynactin 1, susceptibility to - (AD)
Dhtkd1	14.61: Charcot-Marie-Tooth neuropathy Type 2Q - (AD)
Dmd	1.1: Duchenne/Becker Muscular Dystrophies - (XR)<br>10.81: Dilated cardiomyopathy, 3B - (XR)
Dmpk	6.1: Myotonic dystrophy 1 (Steinert) - (AD)
Dnajc5/Csp-alpha	N/A
Dnm2	2.15: Congenital muscular dystrophy related to DNM2 - (AD)<br>3.23: Centronuclear myopathy 1 - (AD)<br>4.16: Distal myopathy related to DNM2 - (AD)<br>12.82: Lethal Congenital Contracture Syndrome 5 - (AR)<br>14.14: Charcot-Marie-Tooth disease, dominant intermediate B - (AD)
Dnmt3a	N/A
*Dok7**	11.18: Myasthenic syndrome, congenital, 10 - (AR)<br>16.27: Fetal akinesia deformation sequence 3 - (AR)
Dst	14.106: Hereditary sensory and autonomic neuropathy type VI - (AR)
Dtna	10.92: Left ventricular noncompaction, 1 - (AD)
Dync1h1	12.32: Spinal muscular atrophy, lower extremity, autosomal dominant - (AD)<br>14.59: Charcot-Marie-Tooth neuropathy Type 2O - (AD)
Ecel1	16.18: Arthrogryposis, distal, type 5D - (AR)
Eef1a2	N/A
Egr2	14.4: Charcot-Marie-Tooth disease, type 1D - (AD)<br>14.27: Neuropathy, congenital hypomyelinating, 1- (AR)<br>14.44: Dejerine-Sottas hypertrophic neuropathy, dominant - (AD digenic)
Erbb2	N/A
Erbb4	12.62: Amyotrophic lateral sclerosis 19 - (AD)
Fat1	N/A
Fbxo45	N/A
Fgfbp1	N/A
Fus	12.49: Amyotrophic lateral sclerosis 6, with or without frontotemporal dementia - (AD)
Gabpa	N/A
Gars	12.21: Distal spinal muscular atrophy, distal with upper limb predominance (type V) - (AD)<br>14.50: Charcot-Marie-Tooth neuropathy Type 2D - (AD)
Gas7	N/A

Table S2. (*continued*)

Gene	Neuromuscular disease (Code: Disease – (Inheritance))
Gdap1	14.21: Charcot-Marie-Tooth disease Type 4A - (AR)<br>14.56: Charcot-Marie-Tooth neuropathy Type 2K - (AD, AR)<br>14.82: Charcot-Marie-Tooth disease, recessive intermediate, A - (AR)
*Gfpt1**	11.20: Myasthenia, congenital, 12, with tubular aggregates - (AR)
Gphn/Geph	N/A
Hspb1	12.18: Neuropathy, distal hereditary motor, type IIB - (AD)<br>14.52: Charcot-Marie-Tooth neuropathy Type 2F - (AD, AR)
Hspg2	6.8: Schwartz-Jampel syndrome - (AR)
Ighmbp2	12.5: Spinal muscular atrophy, distal autosomal recessive 1 (with respiratory distress) - (AR)<br>14.87: Charcot-Marie-Tooth disease, type 2S - (AR)
Itgb1	N/A
Kalrn	N/A
Ky	5.8: Myofibrillar myopathy 7 - (AR)
Lama4	10.71: Dilated cardiomyopathy, 1JJ - (AD)
*Lama5**	11.38: Presynaptic congenital myasthenic syndrome - (AR)
*Lamb2**	11.34: Congenital myasthenic syndrome with nephrotic syndrome - (AR)
Large1	2.34: Congenital muscular dystrophy with hypoglycosylation of dystroglycan - (AR)
Lgals1/Galectin-1	N/A
*Lrp4**	11.25: Myasthenic syndrome, congenital, 17 - (AR)
Lrsam1	14.60: Charcot-Marie-Tooth neuropathy Type 2P - (AD)
Madd/Rab3 GEP	N/A
Mapt	12.99: Lower motor neuron disease with respiratory failure related to MAPT - (AD)
Matr3	4.5: Vocal cord and pharyngeal distal myopathy (VCPDM) reclassified as ALS21 - (AD)<br>12.64: Amyotrophic lateral sclerosis 21 - (AD)
Mbnl1	N/A
Mbnl2	N/A
Met	16.23: Arthrogryposis and muscular dysplasia - (AD)
miR-1/206/133	N/A
miR-218-1/2	N/A
Mmp3/Stromelysin-1	N/A
Mnx1/Hb9	N/A
*MuSK**	11.17: Myasthenic syndrome, congenital, 9, associated with acetylcholine receptor deficiency - (AR)<br>16.26: Fetal akinesia deformation sequence 1 - (AR)
Nbea	N/A
Nefl	14.7: Charcot-Marie-Tooth neuropathy Type 1F - (AD)<br>14.19: Charcot-Marie-Tooth disease, dominant intermediate G - (AD)<br>14.51: Charcot-Marie-Tooth neuropathy Type 2E - (AD)
Nemf	N/A
Nes	N/A
Nmf67	N/A
Nrcam	N/A
Nrg1/ARIA	N/A
Pex10	N/A
Pfn1	12.61: Amyotrophic lateral sclerosis 18 - (AD)
Phr1/Mycbp2	N/A

Table S2. (*continued*)

Gene	Neuromuscular disease (Code: Disease – (Inheritance))
Plaa	N/A
Pls3	N/A
Prkcq/PKCϑ	N/A
Psmf1/PI31	N/A
Rab18	N/A
*Rapsn**	11.19: Myasthenic syndrome, congenital, 11, associated with acetylcholine receptor deficiency - (AR)<br>16.28: Fetal akinesia deformation sequence 2 - (AR)
Rer1	N/A
Ret	N/A
Rims1	N/A
Rims2	N/A
Rnd3/RhoE	N/A
Scn8a/Nav 1.6	N/A
Scyl1	13.76: Spinocerebellar ataxia, autosomal recessive 21 - (AR)
Setx	12.47: Amyotrophic lateral sclerosis 4 - (AD)<br>13.87: Spinocerebellar ataxia with axonal neuropathy type 2 - (AR)
Sh3tc2	14.25: Charcot-Marie-Tooth disease, Type 4C - (AR)
Skor2	N/A
Slc16a3/Mct4	N/A
*Slc18a3**	11.29: Myasthenic syndrome, congenital, 21, presynaptic - (AR)
Slc25a46	12.92: Pontocerebellar hypoplasia with spinal muscular atrophy - (AR)
*Slc5a7**	11.28: Myasthenic syndrome, congenital, 20, presynaptic - (AR)<br>12.26: Spinal muscular atrophy, distal, with vocal cord paralysis (Harper-Young) - (AD)
Slc6a5/Glyt2	N/A
Smn1	12.1: Spinal muscular atrophy , type I (Werdnig-Hoffman) - (AR)<br>12.2: Spinal muscular atrophy, type II (intermediate) - (AR)<br>12.3: Spinal muscular atrophy, type III (Kugelberg-Welander) - (AR)<br>12.4: Spinal muscular atrophy, type IV, adult form - (AR)
*Snap25**	11.26: Myasthenic syndrome, congenital, 18 - (AD)
Snca	N/A
Snta1	10.135: Long QT syndrome 12 - (AD)
Sntb1	N/A
Sntb2	N/A
Sod1	12.43: Amyotrophic lateral sclerosis 1 (dominant) - (AD)<br>12.44: Amyotrophic lateral sclerosis 1 (recessive) - (AR)
Spin1	N/A
Stac3	3.54: Myopathy, congenital, Baily-Bloch (Native American myopathy) - (AR)<br>3.55: Myopathy, congenital, with malignant hyperthermia susceptibility - (AR)
Stxbp5l/Tom2	N/A
Syne1	1.6: Emery-Dreifuss muscular dystrophy 4, autosomal dominant - (AD)<br>10.82: Dilated cardiomyopathy related to nesprin-1- (AD)<br>13.63: Autosomal recessive spinocerebellar ataxia, 8 - (AR)<br>16.21: Arthrogryposis multiplex congenita with nesprin-1 defect - (AR)
Syne2	1.7: Emery-Dreifuss muscular dystrophy 5, autosomal dominant - (AD)
*Syt2**	11.14: Myasthenic syndrome, congenital, 7, presynaptic - (AD)<br>11.15: Myasthenic syndrome, congenital, 7B, presynaptic - (AR)<br>12.37: Distal motor neuropathy related to SYT2 - (AD)
Tardbp	12.53: Amyotrophic lateral sclerosis 10, with or without frontotemporal dementia - (AD)

Table S2. (*continued*)

Gene	Neuromuscular disease (Code: Disease – (Inheritance))
Tmem106b	N/A
Tmem184b	N/A
Uchl1	15.67: Spastic paraplegia 79, autosomal recessive - (AR)
Usp14	N/A
Utp14b	N/A
Utrn	N/A
Vapb	12.35: Spinal muscular atrophy, late-onset, Finkel type - (AD)<br>12.51: Amyotrophic lateral sclerosis 8 - (AD)

Table S3. Novel candidate genes for NMJ-related diseases in humans based on phenotypes from mouse models registered at the Mouse Genome Informatics MGI database. Extended. All models are constitutive unless otherwise stated. Lethal phenotypes can have complete or incomplete penetrance. ACh: acetylcholine; AChR: acetylcholine receptor; ALS: Amyotrophic lateral sclerosis; cKO: constitutive KO; CT: connective tissue; DGC: dystrophin-associated glycoprotein complex; DM: Myotonic dystrophy; FSHD: Facioscapulohumeral muscular dystrophy; MN: motor neuron; NMJ: neuromuscular junction; PM: point-mutation; SC: Schwann cell; Skm: skeletal muscle; WT: Wild-type.

Gene	Model	Phenotype	Lethality[1]
**Presynaptic terminal**			
MN identity, function and survival			
Adarb1/Adar2	MN-specific KO (*VAChT-Cre*)	Degeneration of MN axons resulting in denervation of NMJs	PD
Clp1	Kinase-dead isoform	MN axonal degeneration leading to MN apoptosis and subsequent denervation of NMJs	SD: V, NL or PD
Gphn/Geph	cKO	Increased MN survival, increased number of NMJs and axon branching capacity, and reduced motor activity in limbs, where glycine has an inhibitory role. The opposite effects are observed in the diaphragm, where this neurotransmitter is excitatory. No changes in NMJ morphology or in the postsynaptic terminal in either case	SD: V or NL
Mnx1/Hb9	cKO	Defective innervation of the diaphragm -but not of limb muscles- due to misrouting of axons. In this muscle, which is thinner than normal, AChR clusters are dispersed, abnormally small and not correctly apposed to the presynaptic terminal because the lack of nerve-derived agrin in the absence of MNs, interferes with postsynaptic differentiation	PNL
Pex10	Chemically induced PM affecting protein function	SCs are reduced in number, abnormally shaped and not properly placed in the NMJ, causing a progressive loss of MN axon integrity. Reduced apposition of pre- and postsynaptic terminals, with many axons extending beyond the endplate. Some axons lack synaptic vesicles, while postsynaptic terminals are normal. Diminished synaptic transmission during development	PNL
Ret	cKO and cranial MN-specific KO (*Nkx6.2-Cre*)	NMJs in cKOs are less developed, as exemplified by presynaptic nerve sprouting and impaired postsynaptic AChR clustering. MN-specific KOs have a reduced number of NMJs but they are mostly morphologically normal	PNL (cKO)
Slc6a5/Glyt2	cKO by spontaneous retrotransposon insertion	Accelerated synapse elimination to achieve fiber monoinnervation, as well as accelerated switch from embryonic to adult AChR subunit types, concomitant with an increase in AChR transcription in the postsynaptic cell. There is a trend towards smaller but normally shaped NMJs	PL
Channel-related			
Cacna2d2	Spontaneous mutation leading to a shorter transcript (*Ducky* mice)	Small reduction of evoked ACh release, but rather than a consequence of reduced voltage-activated Ca2+ channel function, it could be attributable to the smaller size of ducky mice, which present reduced NMJ size as well as muscle fiber diameter	SD: V or PM
Cacng2	Hypomorph by spontaneous transposon insertion (*Stargazer* mice)	Smaller NMJ size	-

Table S3. (*continued*)

Gene	Model	Phenotype	Lethality[1]
Regulation of gene expression at the transcript and protein levels. Proteostasis and autophagy			
Borcs5	cKO	Neuroaxonal dystrophy. Normal NMJ morphology	PNL
Dnmt3a	Nervous system-specific KO (*Nes-Cre*)	Normal looking MNs in the spinal cord, with normal innervation, but high NMJ fragmentation. No signs of skeletal muscle degeneration	PD
Eef1a2	cKO by spontaneous PM (*Wasted* mice)	Aggressive, recessive and early-onset form of neurodegeneration characterized by reactive gliosis in spinal cord, followed by progressive retraction of MN axons from muscle and MN death. Extensive muscle wasting that cannot be solely explained by denervation, but overexpression of *Eef1a2* in muscle does not ameliorate the phenotype	PD
Nemf	cKO and chemically induced PM affecting protein function	The PM models show denervation and fragmentation of the postsynaptic terminals, progressive axonal degeneration, and specific abnormalities in skeletal muscle that depend on the model. cKO mice show more severe phenotypes	AD: V or PD
miR-218-1/2	cKO	Normal MN fate specification and development, but axons fail to form proper NMJs at the target muscles, as exemplified by reduced terminal branching and the lack of innervation of the majority of AChR clusters. Progressive MN loss is also observed	NL
Plaa	cKO and G23V PM recapitulating human PLAA-associated neurodevelopmental disorder	Homozygous mice for the G23V point mutation show swelling and/or sprouting of axon terminals, a reduced number of synaptic vesicles and the presence of enlarged endosomes and vacuoles, as well as muscle atrophy. These defects lead to altered synaptic transmission. Compound G23V/- mutants present a more severe phenotype while homozygous KOs die before birth	AD: PM or PNL
Psmf1/PI31	MN-specific KO (Mnx1-Cre2)	Presynaptic terminals appear fragmented, with accumulation of polyubiquitinated proteins and massive axonal sprouting, while muscles get atrophied. Phenotypes get progressively more severe with age	- (EL cKO)
Rab18	cKO	Disorganization of the cytoskeleton in presynaptic terminals, with randomly oriented filaments. No abnormalities in myelination, endplate area or in the morphology of postsynaptic terminals, although progressive muscle weakness is observed. No dying-back pathology. In essence, no hallmark features of neurodegeneration	PNL
Tmem106b	cKO	MN axons are slightly but significantly shorter, and contain vacuoles. There is a high percentage of partially or fully denervated NMJs	-
Tmem184b	cKO	Presynaptic terminals present large dystrophic swellings and an abnormal accumulation of autophagosomal structures. Postsynaptic AChRs are virtually absent in areas of presynaptic swelling, thus causing reduced apposition of the pre- and postsynaptic terminals. Muscle fibers occasionally accumulate autophagosomes/lysosomes as well. Neuroprotection is illustrated by a short but significant delay in axon degeneration after injury	-

Table S3. (*continued*)

Gene	Model	Phenotype	Lethality[1]
Usp14	Hypomorphs by chemically induced PM (nmf375 mice) and by spontaneous PM (Ataxia mice)	Specific phenotypes depend on the mouse strain and mutant allele. nmf375 mice show progressive defects in NMJs with a significant upregulation of AChRs subunits, altered synaptic transmission, abnormal accumulation of neurofilaments at presynaptic terminals and a reduction in the levels of monomeric ubiquitin. axJ mice exhibit more severe phenotypes, especially at the presynaptic terminals, with large neurofilament accumulations, terminal swelling and sprouting, poor terminal arborization, NMJ denervation, and reduced ACh release, concomitant with defective synaptic transmission. Additionally, muscle size is reduced and the distribution of AChRs reflects an immature stage	SD: V, PNL or PD
<u>Cytoskeleton dynamics</u>			
Basp1/Cap23	cKO	Defect in stimulus-induced nerve sprouting, expanded synaptic regions, and presence of tSC processes	PL
Clip3/CLIPR-59	cKO	Lower density of nerve terminals and reduced number of terminals capped by tSCs, leading to a defect in MN axon stability during late embryogenesis	PNL
Cpeb4	Dominant negative truncated protein	Defect in neurite outgrowth and consequently, in NMJ formation	NL
Fbxo45	cKO	Incomplete innervation of the diaphragm, excessive branching of axon terminals, sometimes extending across the endplate bands, which are narrower than in WT conditions	NL
Phr1/Mycbp2	cKO, MN-specific deletion (*Mnx1-Cre*[2]), C-terminal truncated protein (*Magellan* mice), mutant lacking exons 8-9, and PM affecting protein function	MN axon terminals show excessive sprouting and varicose endings, even extending extrasynaptically, due to defective growth cones that cause navigation errors. These defects lead to fewer axons reaching their targets in muscle and ultimately, to impaired synaptic transmission. No signs of increased MN death or axon degeneration. Depending on the model, either diffuse AChR clustering or densely packed and narrow endplate bands are found at the postsynaptic terminal	AD: V, PNL or NL
Rnd3/RhoE	cKO	Less number of MNs, immature NMJs and absence of the peroneal nerve, which results in the atrophy of the target muscles. The rest of the muscles are smaller, without signs of degeneration/regeneration	PD

Table S3. (*continued*)

Gene	Model	Phenotype	Lethality[1]
Defective signaling among tSCs, pre-, and post-synaptic terminals. Defective synaptic transmission			
Nrcam	cKO by spontaneous B2 insertion; compound *Nrcam/Gars* and *Nrcam/Sh3tc2* mutants	Single *Nrcam* mutants do not show an overt neuromuscular phenotype, similarly to *Sh3tc2* single mutants. Double KOs show the typical demyelinating effects of *Sh3tc2* mutants as well as NMJ fragmentation, nerve terminal sprouting, extrasynaptic AchR expression and defective action potential conduction at the presynaptic terminal. Similar phenotypes in double *Gars*$^{+/-}$*Nrcam*$^{-/-}$ mutants, with additional muscle atrophy due to *Gars* haploinsufficiency	PD (*Nrcam/ Sh3tc2* KOs)
Scn8a/Nav1.6	cKO by spontaneous LINE1 insertion (*Med* mice); cKO by spontaneous PM (*m10J* mice); compound *Scn8a/Gars* mutants	In *Med* KO mice, axons appear thick, swollen, with reduced branching and high content of vesicles, presumably due to impaired synaptic transmission. Reduction in the number of tSCs is the first observed cellular defect. Delayed maturation of NMJs: they are smaller and abnormally shaped. Increased presence of multi-innervated fibers. No denervation and close-to-normal apposition of pre- and postsynaptic terminals are observed. The NMJ morphological defects and the reduction in nerve conduction velocity characteristic of *Gars* mutants are exacerbated in the case of *Scn8a* haploinsuffiency	SD and AD: V or PL
Endocytosis, synaptic vesicle formation and neurotransmitter release			
Dnajc5/Csp-α	cKO	Immature postsynaptic terminals, reduced apposition of pre and post-synaptic terminals, vacuolization of presynaptic terminals and presence of multilamellar bodies, invagination of presynaptic terminals by tSCs and impairment of synaptic transmission	PD
Nbea	cKO by random integration that in addition disturbs GH secretion causing dwarfism; cKO by gene trap	Block of synaptic transmission, albeit with normal action potential conduction along MNs and normal muscle function. NMJ morphology appears unaltered	NL
Madd/Rab3 GEP	cKO	Normal nerve conduction at the presynaptic component, but 10-fold reduction in vesicle content, with many of the few existing vesicles being large, empty and/or mislocalized, causing impaired neuromuscular transmission. Axon terminals are enlarged and contain degenerated mitochondria. Normal NMJs and postsynaptic specializations	SD: V or NL
Pls3	Overexpression in SMA models (*Smn1*$^{-/-}$/*Tg(SMN2)*)	In mild SMA mouse models, *Pls3* overexpression stabilizes axons, and restores synaptic vesicle recycling and release processes at the presynaptic terminal. At the postsynaptic level, endplate and muscle fiber sizes are increased. All in all, NMJ functionality, motor function and animal survival are moderately ameliorated in these mice. In contrast, in severe SMA models only mild protection effects are observed, and they are insufficient to improve survival or motor function	PD

Table S3. (*continued*)

Gene	Model	Phenotype	Lethality[1]
Rims1 and *Rims2*	cKO	NMJs are mostly morphologically normal, although there is extensive nerve terminal branching that covers a broader than usual area in the diaphragm, possibly in response to the impaired synaptic transmission observed in the double mutants, which die immediately after birth. In contrast, single mutants are viable and thus, some functional compensation between paralogs presumably takes place, although there are no compensatory changes at protein level in either single model. Single *Rims1*$^{-/-}$, but not single *Rims2*$^{-/-}$ mice, show impaired synaptic transmission as well	NL
Stxbp5l/Tom2	cKO	No morphological defects in MNs, NMJs or muscle, but altered synaptic transmission: enhanced spontaneous ACh release and increased synaptic fatigue upon sustained stimulation	SD: V or PL
<u>Unknown</u>			
Slc16a3/Mct4	cKO	No impact in MN survival but axon diameter and length are increased in the *Mct4* mutants. Muscle-specific progressive degeneration of the NMJs is observed, with progressive fragmentation in the postsynaptic terminals. These defects are accompanied by abnormal synaptic transmission. Muscles have normal morphology and contractility even if they accumulate high amounts of intramuscular lactate	-
Snca	Constitutive overexpression of mouse WT or human PD-associated A53T mutant form	MN axons degenerate and retract from NMJs, thus leading to muscle denervation in both models, with mice overexpressing the human mutant A56T SNCA protein showing a more severe phenotype. In the case of the mouse WT Snca overexpression model, muscles show some signs of atrophy, and motor impairment appears later and in a much less pronounced manner than in the case of human WT SNCA overexpression. Pronounced ubiquitin immunopathology is also observed in the spinal cord	AD: V or PD
**Synaptic cleft**			
<u>Channel-related</u>			
Mmp3	cKO	Extensive remodeling of the postsynaptic membrane: increased density of AChR clusters, bigger endplate areas, increased number and size of junctional folds, as well as ectopic junctional folds. All these morphological changes have little electrophysiological impact	-
<u>Defective signaling among tSCs, pre-, and post-synaptic terminals. Defective synaptic transmission</u>			
Fgfbp1	cKO	Increased fragmentation of NMJs, suggesting accelerated ageing, but no signs of denervation. Increased expression of some AChR subunits as well as of atrogenes in the muscle terminal	-

Table S3. (*continued*)

Gene	Model	Phenotype	Lethality[1]
Adhesion			
Aplp2 and *App*	Skeletal muscle- (*Ckmm-Cre*), nervous system- (*Nes-Cre*), neuron-specific (*ChAT-Cre*) or constitutive *App* deletion in a *Aplp2* cKO background; humanized mutated and truncated *App* transgene in a *Aplp2* cKO background; constitutive deletion of the *Lrp4* transmembrane domain in an *App* cKO background	In double *App/Aplp2* KOs, NMJs show a diffuse patterning of the synaptic terminals with reduced apposition between them. There is excessive sprouting of the presynaptic terminal, which in addition contains a reduced number of synaptic vesicles. All these phenotypes are concomitant with defects in synaptic transmission	AD: V, PNL, PL or PD
Itgb1	Neural crest- (*PLAT-Cre*), nervous system- (*Nes-Cre*) or skeletal muscle-specific (*ACTA1-Cre*) KOs	In tSCs-specific *Itgb1* mutants, there is a nearly complete absence of tSCs at the presynaptic terminals, and defects in axon arborization and morphology are observed. NMJs appear immature, with abnormal ECM composition and organization. Failure of many axons to establish functional NMJs. Abnormal density of AChR clusters at the postsynaptic terminal, and progressive muscle atrophy. When *Itgb1* is absent from muscles, presynaptic terminal morphology is altered, synaptic contacts are not correctly established, muscles are smaller and mice die at birth	PD
Fat1	Skeletal muscle-specific (*Pax3-Cre*) hypomorph; skeletal muscle- (*Pax3-Cre*), MN- (*Olig2-Cre*), mesenchymal- except cranial; (*Prx1-Cre*), craniofacial mesenchymal-specific (*Wnt1-Cre*) or constitutive mutant lacking the transmembrane domain	Altered coupling of muscular and neuronal morphogenesis. In all constitutive, MN-, CT- or Skm-specific *Fat1* mutants, NMJs deteriorate and the migration of myogenic progenitors is compromised, albeit at various extents. Muscle defects are more severe in Skm-specific *Fat1* mice, which present abnormal shape and position of specific muscle groups, in particular, of those associated with FSHD. These mutant muscles exhibit misoriented fibers, necrosis, and reduced fiber size	AD: V, NL or PD

Table S3. (*continued*)

Gene	Model	Phenotype	Lethality[1]
**Postsynaptic terminal**			
Channel-related			
Csnk2b	Skeletal muscle-specific KO (*ACTA1-Cre*)	Fragmentation and disintegration of the AChR clusters at the postsynaptic terminal, disrupting the typical pretzel-like shape. Impairment of synaptic transmission by affecting sustained neurotransmitter release and reducing post-synaptic sensitivity to Ach	-
Nes	cKO	Increased number of AChR clusters, nerve length and endplate area. Reduced skeletal muscle mass due to frequent spontaneous regeneration.	-
Prkcq/PKCθ	cKO	Delayed activity-dependent synapse elimination process to achieve monoinnervation of NMJs during the early postnatal period by about 4 days. Delayed maturation of the presynaptic terminal. Beyond this point, NMJ morphology and function were normal. No other abnormalities	-
Rer1	Heterozygous cKO	Smaller normally-shaped NMJ size that precedes a reduction in muscle fiber size. The amount of fully assembled AChR receptors is reduced	- (EL cKO)
Regulation of gene expression at the transcript and protein levels. Proteostasis and autophagy			
Mbnl1 and *Mbnl2*	*Mbnl1*$^{-/-}$/*Mbnl2*$^{+/-}$	DM is caused by expansion of specific repeats in the *DMPK* or *CNBP* genes. These expansions sequester Mbnl proteins. In accordance, compound *Mbnl1/2* mutant recapitulate the DM-relevant features: NMJs appear immature and fragmented, while many muscle fibers are atrophied, split or contain central nuclei	PD (EL double KO)
miR-1/206/133	cKO	*miR-206* is required for efficient regeneration of NMJs after acute nerve injury in a mouse model for ALS, but not in *mdx* mice, a commonly used model for muscle regeneration. The absence of *miR-206* alone has no effect in NMJ formation during development. In contrast, in triple *miR-1/206/133* cKO mice, AChR clusters are dispersed and reduced in number and size, while axons extend to extrasynaptic areas, leading to defects in NMJ formation and maintenance. No effects in muscle development are observed in any model	PD
Spin1	Skeletal muscle-specific KO (*Myf5-Cre*)	Severe muscle deficiencies including sarcomere disorganization, fiber degeneration and necrosis, defective mitochondrial network and abnormal glycogen accumulation. The morphology of the presynaptic membrane is also altered, and nerve terminals contain vacuoles and a reduced number of vesicles	NL
Endocytosis, synaptic vesicle formation and neurotransmitter release			
Erbb2 and Erbb4	Skeletal muscle-specific single *Erbb2* or double *Erbb2/Erbb4* KOs (*ACTA1-Cre*)	Normal presynaptic terminals. However, postsynaptic terminals contain reduced levels of AChRs due to their migration to the perisynaptic membrane, thus causing postsynaptic fragmentation. Other scaffold proteins such as MuSK and rapsyn also relocate to perisynaptic areas. Synaptic transmission is mildly compromised	-
Gabpa	Skeletal muscle-specific KO (*ACTA1-Cre*)	Two different skeletal muscle specific models show different phenotypes: mild NMJ morphological and functional defects with altered expression of some AChR subunits in one model, no phenotype in the second model	-
Nrg1/ARIA	Heterozygous cKO	Reduced postsynaptic AChR density	- (EL cKO)

Table S3. (*continued*)

Gene	Model	Phenotype	Lethality[1]
The DGC			
Stnb1 and *Stnb2*	Single *Snta1*, *Sntb1*, or *Sntb2*, double *Snta1/Sntb2* or triple *Snta1/Sntb1/Sntb2* cKOs	Disturbed NMJs morphology in *Snta1-/-* single mutants that is exacerbated in the double *Snta1*$^{-/-}$/*Sntb2*$^{-/-}$ KOs, while single *Sntb2*$^{-/-}$ mice remain mostly unaltered. Thus, double mutants have few and aberrant functional folds, as well as reduced levels of AChRs, which in addition are abnormally distributed. Also, while dystrophin and dystrobrevin are still retained at the DGC complex, utrophin and nNOS are absent. The lack of nNOS could further contribute to postsynaptic membrane destabilization since it controls the expression of specific components of the integrin complex. Despite all these defects, synaptic transmission is mostly normal and no muscular dystrophy is observed. Triple *Snta1/Sntb1/Sntb2* KO mice have reduced protein expression and sarcolemmal targeting of dystrophin, and a much more severe outcome in muscle functional tests due to significant left ventricular cardiac hypertrophy	-
Utrn	Single Utrn, double Utrn/Dmd or triple Utrn/Dmd/Dnta cKOs	Single Utrn or Dmd mutants show reduced density of otherwise normally clustered AChR, and reduced number of junctional folds. The double Utrn/Dmd mutants exhibit abnormal AChR distribution and complete absence of postsynaptic folding. Only minimal electrophysiological changes in these three models. Defects in AChR distribution are due to the loss of α-dystrobrevin association to the DGC, suggesting that this protein maintains the crests and depths of functional folds as molecularly distinct domains. Its absence in single Dnta and triple Utrn/Dmd/Dnta KOs leads to instability of postsynaptic specializations and eventually to NMJ deterioration	AD: V or PD
Unknown			
Kalrn	cKO or nervous system-specific KO (*Nes-Cre*)	Morphological abnormalities in pre and postsynaptic terminals, with decreased number of junctional folds in the latter case. Altered NMJ shape and sarcomere length in the target muscle	SD: V or PD
**Unknown**			
Unknown			
Gas7	Truncated, lowly expressed and unstable hypomorph	Decreased number of MNs, which show reduced terminal sprouting in adult NMJs. Changes in muscle fiber type composition and size only of slow muscles due to either MN innervation/denervation or defects in postsynaptic specialization	-
Skor2	cKO	90% KO pups die within 48 hours of birth due to failure to nurse, defective NMJ function, and/or respiratory failure	PNL

[1] Key: AD: allele or model-dependent; EL: embryonic lethality; NL: neonatal lethality; PD: premature death; PNL: perinatal lethality; PL: postnatal lethality; SD: strain-dependent; V: viability.
[2] See section *2.10. Other considerations* in the main text for further information regarding this line.
